# LncRNA *HOTTIP* modulated by Hedgehog signaling drives colorectal cancer progression by promoting HUWE1-mediated ubiquitin‒proteasome degradation of p53

**DOI:** 10.1038/s41419-025-07817-4

**Published:** 2025-07-07

**Authors:** Hui Wang, Weiwei Jiao, Defu Li, Zhengping Yu, Huqiao Luo, Jie Zhang, Yuanbing Zhang, Hai Rao, Quqin Lu, Bing Zhao, Shiwen Luo

**Affiliations:** 1 Center for Experimental Medicine, The First Affiliated Hospital of Nanchang University; The MOE Basic Research and Innovation Center for the Targeted Therapeutics of Solid Tumors; Jiangxi Medical College, Nanchang University, Nanchang, Jiangxi China; 2 Department of Pathology and Institute of Molecular Pathology, Jiangxi Provincial Key Laboratory for Precision Pathology and Intelligent Diagnosis, The First Affiliated Hospital of Nanchang University, Nanchang, Jiangxi China; 3 State Key Laboratory of Reproductive Regulation and Breeding of Grassland Livestock, Institute of Biomedical Sciences, School of Life Sciences, Inner Mongolia University, Hohhot, China; 4 Department of Epidemiology and Biostatistics, Jiangxi Provincial Key Laboratory of Disease Prevention and Public Health, School of Public Health, Jiangxi Medical College, Nanchang University, Nanchang, Jiangxi China; 5 School of Medicine, Shanghai University, Shanghai, China; 6 Department of Biochemistry, School of Medicine, Southern University of Science and Technology, Shenzhen, China

**Keywords:** Mechanisms of disease, Colorectal cancer

## Abstract

The Hedgehog (Hh) pathway plays critical roles in regulating appropriate tissue morphogenesis and organ formation in the gastrointestinal tract, and its dysregulation has been closely linked to the malignant progression of colorectal cancer. However, the underlying mechanisms are poorly understood. Here, we report that the long noncoding RNA HOXA distal transcript antisense RNA (lncRNA *HOTTIP*) is a novel Hh target gene and a Hh pathway effector. Activation of Hh signaling induced the expression of the lncRNA *HOTTIP*. The overexpression of the lncRNA *HOTTIP* promoted colorectal cancer cell proliferation and tumor growth, whereas *HOTTIP* knockout exerted the opposite effects in vitro and in vivo. Compared with control mice, AOM/DSS-colorectal cancer model mice with lncRNA *Hottip* overexpression in Villin-Cre epithelial cells presented notable increases in tumor number and size. Moreover, lncRNA *HOTTIP* overexpression increased the proliferation and viability of organoids derived from colorectal cancer patients. Mechanistically, the lncRNA *HOTTIP* binds to HUWE1 and promotes its E3 ubiquitin ligase activity toward p53 and mediating HUWE1-dependent proteasomal turnover of p53, which decreases the level and activity of p53 and results in the overexpression of p53 pathway downstream target genes important for cell proliferation and survival. From a clinical perspective, high levels of the lncRNA *HOTTIP* were detected in colorectal cancer tissues and were strongly correlated with poor prognosis in colorectal cancer patients. Together, our findings demonstrate that the lncRNA *HOTTIP*, a novel effector of the Hh pathway, drives colorectal cancer progression by promoting HUWE1-dependent ubiquitin‒proteasome degradation of p53. These findings reveal critical roles of the Hh-*HOTTIP*-p53 signaling axis in colorectal cancer progression and suggest a potential therapeutic target for colorectal cancer.

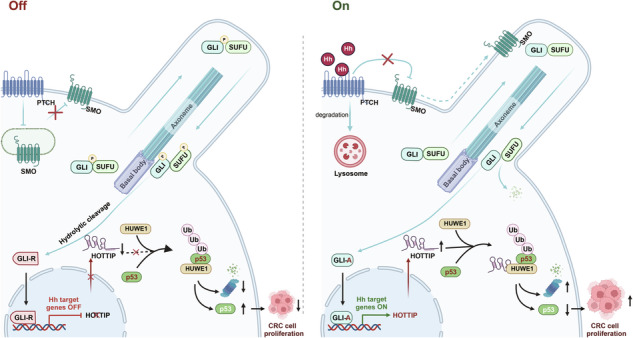

## Introduction

Colorectal cancer is one of the most common cancers worldwide and is characterized by significant morbidity and mortality rates [1, 2]. The tumorigenesis and progression of colorectal cancer are extremely complex processes involving the interaction of multiple factors, such as mutations in oncogenes and tumor suppressor genes, chronic inflammatory stimulation, and dysregulation of signaling pathways. However, the underlying mechanisms are still poorly understood. Therefore, it is important to identify colorectal cancer-related genes and elucidate the molecular mechanisms underlying the malignant behaviors of colorectal cancer cells.

The Hedgehog (Hh) signaling pathway is an important regulator of embryonic development and homeostasis in metazoans [3, 4]. Hh signaling is activated by Hh ligands, including Sonic, Indian, and Desert hedgehog, with subsequent activation of its downstream cascade, which comprises Patched (PTCH), Smoothened (SMO), Suppressor of Fused (SuFu), and the GLI family of zinc finger transcription factors GLI1, GLI2 and GLI3 [5]. In mammals, the GLI repressor (Gli^R^) function is performed mostly by GLI3, whereas the GLI activator (Gli^A^) function is performed mainly by GLI2 [6]. GLI1 is a direct transcriptional target of the Hh pathway and participates in a positive feedback loop to reinforce Gli^A^ function [7]. Among the three GLI homologs, GLI2 is thought to play a more critical role in the Hh pathway because the deficiency of GLI2, but not GLI1, in mice leads to embryonic lethality at later embryonic stages [8]. In germ cells, dysregulation of the Hh pathway leads to various congenital abnormalities [9–11]. However, aberrant activation of Hh signaling in somatic cells has been implicated in human cancers [12]. Dysregulation of Hh signaling promotes the expression of a series of oncogenes by activating GLI transcription factors [13]. These genes regulate cellular processes associated with tumorigenesis, including tumor cell survival/proliferation and metastasis, and cancer stem cell self-renewal [14, 15]. Indeed, numerous oncogenes, such as SOX2, c-Myc, BCL2, and FOXM1 [16–18], have been identified as Hh targets. The overexpression of these oncogenes due to dysregulation of Hh signaling plays critical roles in the initiation and maintenance of multiple types of tumors, such as melanoma [19], glioblastoma [20], hepatocellular carcinoma [17], and colorectal cancer [18, 21]. Interestingly, several long noncoding RNAs (lncRNAs), such as Tsix [22], Hilnc [23], and SOX2OT [24], have been linked to Hh signaling, but the roles of other potential lncRNAs in cell proliferation and tumorigenesis remain to be explored.

HOXA distal transcript antisense RNA (*HOTTIP*) is a HOXA locus-associated lncRNA transcribed from the 5’ tip of the HOXA gene. In various cancers, *HOTTIP* is frequently upregulated and plays a critical role in promoting tumor initiation and progression. The lncRNA *HOTTIP* functions as an epigenetic regulator that recruits the WDR5/MLL complex to coordinate active chromatin modifications and HOXA gene expression [25]. Transgenic overexpression of *Hottip* has been found to increase hematopoietic stem cell self-renewal by altering the HOXA topologically associated domain (TAD) and its transcription [26]. *HOTTIP*-mediated R-loop formation directly reinforces CTCF-mediated chromatin boundary activity and TAD integrity to drive oncogene transcription and leukemia development [27]. *HOTTIP* modulates cancer stem cell properties in human pancreatic cancer by regulating HOXA9 [28]. *HOTTIP* functions as an oncogene in small cell lung cancer by sponging miR-574-5p and positively regulating the expression of EZH1 [29]. *HOTTIP* binds to HOXA13 promoters and mediates gene activation through H3K4me3 via the WDR5-MLL1 complex in pancreatic ductal adenocarcinoma [30]. Additionally, *HOTTIP* can potentially accelerate the immune escape of ovarian cancer cells by increasing IL-6-dependent PD-L1 expression [31]. Furthermore, recent studies have shown that *HOTTIP* is specifically upregulated in small cell lung carcinoma [29], gastrointestinal cancer [32, 33], pancreatic cancer [28], hepatocellular carcinoma [34], osteosarcoma [35], and ovarian cancer [31]. In colorectal cancer, *HOTTIP* is highly expressed, promoting cell proliferation, migration, and invasion [36], while knockdown of *HOTTIP* inhibits these processes and induces apoptosis by targeting SGK1 [37]. While these findings highlight *HOTTIP* as an important player in colorectal cancer pathogenesis, the detailed molecular mechanisms through which *HOTTIP* contributes to colorectal cancer progression are still not fully understood.

p53, encoded by the TP53 gene, is vital for genomic integrity and orchestrates cellular responses to genomic stress, including cell cycle arrest, senescence, DNA repair, and apoptosis [38]. p53 functions by regulating numerous downstream targets, such as p21 [39], Bax [40], PUMA [41], and BTG2 [42]. Loss or mutation of p53 occurs in more than 50% of cancers, including colorectal cancer, where it is linked to hyperproliferation, apoptosis resistance, and stemness. Various posttranslational modifications, such as ubiquitination, phosphorylation, acetylation, and methylation, influence the role of p53 in tumorigenesis [43–46]. The ubiquitin‒proteasome pathway plays a crucial role in regulating the p53 protein level. MDM2, which functions as an E3 ubiquitin ligase, promotes p53 degradation. HUWE1 is another well-known E3 ligase that targets p53 and many other substrates involved in tumorigenesis [47]. In addition, lncRNAs have been implicated in the posttranslational regulation of p53. For example, the lncRNA NRON promotes tumorigenesis by enhancing MDM2 activity toward tumor suppressor substrates [48]. While lncRNAs are known to be involved in p53 pathways, the roles of lncRNAs in regulating the p53 protein itself remain largely unknown.

In this study, we identified the lncRNA *HOTTIP* as a novel Hh target gene. We further performed a set of experiments to elucidate the molecular mechanisms by which *HOTTIP* promotes the proliferation and malignant progression of colorectal cancer. Our findings indicate that by binding to the E3 ubiquitin ligase HUWE1, *HOTTIP* promotes HUWE1-dependent ubiquitination and proteasomal degradation of p53.

## Materials and methods

### Reagents, plasmids, and antibodies

The reagents utilized in this study, along with their sources, are listed in Table S1. The pCDH-CMV and pLVX-Myc vectors expressed human *HOTTIP* and GLI2, respectively, whereas the pGMLV and Tet-pLKO-puro vectors were used for their knockdown (see Table S2 for short hairpin RNA (shRNA) sequences). The antibodies and sources are listed in Table S3.

### Cell culture and transfection

The HEK-293T, HCT116, HT-29, SW620, SW480, DLD-1, and CCC-HIE-2 cell lines were obtained from ATCC and other sources. All the cell lines were authenticated using short tandem repeat profiling and were negative for mycoplasma contamination, as detected via a PCR-based assay. All the cells were cultured in appropriate media supplemented with 10% FBS and antibiotics. Transfection was performed using Lipofectamine 2000 (11668019, Thermo Fisher Scientific).

### qPCR and RNA-seq

Total RNA was extracted with TRIzol, and complementary DNA was synthesized using the PrimeScript® RT reagent Kit (DRR047A, Takara). qPCR was conducted on an ABI StepOnePlus™ system using SYBR® Premix Ex Taq II (DRR820A, Takara), and gene expression was quantified via the 2^−ΔΔCT^ method. The primer sequences utilized for the qPCR analysis are provided in Table S4.

### Immunoblotting and subcellular fractionation

The cells were lysed, and the proteins were separated by SDS‒PAGE, followed by transfer to nitrocellulose membranes for detection using the ECL system. Subcellular fractions were obtained using the PARIS™ Kit (AM1921, Thermo Fisher Scientific) and analyzed via Western blotting and qPCR.

### CRISPR/Cas9 knockout system

*HOTTIP*-knockout HT-29 cells were generated via CRISPR/Cas9 genome editing. In brief, single guide RNA (sgRNA) sequences targeting the *HOTTIP* gene were designed with an online CRISPR design tool (http://tools.genome-engineering.org) and subsequently cloned and inserted into the LScKO-4G vector. The sgRNAs used in this study to generate the *HOTTIP*-knockout HT-29 cells are listed in Table S5.

### ChIP and luciferase assays

ChIP experiments were carried out in HT-29 cells according to a standard protocol. The primers used are shown in Table S6. For the dual-luciferase reporter assays, the pcDNA3.1-myc-GLI2 plasmid was transfected into HEK-293T cells together with the wild-type or mutated pGL4.20-*HOTTIP* reporter and Renilla luciferase plasmids. The primers used to generate the luciferase reporter constructs are listed in Table S7.

### RNA pulldown assay and mass spectrometry

The binding of *HOTTIP* to proteins was detected via an RNA pulldown assay in DLD-1 and HT-29 cells, followed by mass spectrometry for identification. For the mass spectrometry data of the proteins identified in this study, see Table S8.

### RIP

The cells were lysed, and RNA was immunoprecipitated using anti-Flag antibodies. The enriched RNA was isolated and analyzed via qPCR.

### Cell proliferation assay

Cell viability was measured via a Cell Counting Kit-8 (CCK8) assay. A colony formation assay was also performed to evaluate the proliferative capacity of colorectal cancer cells. In addition, a Cell-Light EdU DNA Cell Proliferation Kit (GK10001, GLPBIO) was used following the manufacturer’s instructions to evaluate the cell proliferation potential.

### Flow cytometry

Flow cytometry was used to assess the cell cycle distribution and Ki-67 expression. For cell cycle analysis, the cells were harvested and fixed with 70% ethanol. After being washed with PBS, the cells were resuspended in 0.3 ml of PI/RNase Staining Buffer, stained for 15 min at room temperature, and then subjected to flow cytometric analysis. The expression level of Ki-67 in the colorectal cancer organoid lines was evaluated via flow cytometry (FACS Celesta, BD Biosciences), and the data were analyzed with FlowJo software.

### FISH and immunohistochemistry analysis

DIG-labeled sense (F, forward) and antisense (R, reverse) *HOTTIP* probes were synthesized using a DIG RNA labeling kit. For RNA FISH, cells were seeded on multichamber slides, fixed, and permeabilized. The slides were hybridized with the DIG-labeled RNA probe, followed by digestion with excess probe and washing. An HNPP Fluorescent Detection Set was used for detection per the manufacturer’s protocol.

For immunohistochemistry, paraffin sections (3 μm thick) of formalin-fixed tissue were dewaxed, rehydrated, and treated with 3% hydrogen peroxide for 10 min. Antigen retrieval was performed by heating the sections in EDTA buffer (pH 9.0) for 24 min. After blocking with 10% normal goat serum, the sections were incubated with the specified antibodies.

### Mouse models

For xenograft experiments, 2 × 10^7^ stably transduced HCT116 (Lv-Ctrl and Lv-*HOTTIP*) and HT-29 (Ctrl-KO and *HOTTIP*-KO) cells were injected subcutaneously into the bilateral inguinal region of 5-week-old female BALB/c-nu mice (SJA Laboratory Animal Co., Ltd, Hunan, China). Tumor size was monitored with Vernier calipers every 3 days. After 4 weeks, the xenografts were harvested for immunohistochemistry analysis, with six mice per group.

A *Hottip* knock-in mouse model was established via the CRISPR/Cas9 system by Beijing Biocytogen Co., Ltd (Beijing, China). B6. Tg^(Vil1-Cre)997Gum/J^ (Vil-Cre) mice were obtained from the Shanghai Model Organisms Center. F_1_ offspring carrying the mutant allele were identified by PCR via the strategy indicated in Table S9. *Hottip*^LSL/+^ mice were crossed with Vil-Cre mice to generate *Hottip*^LSL/+^; Vil-Cre mice, and mice lacking the Vil-Cre transgene were used as controls.

For the inflammation-dependent colorectal cancer model, mice were injected with 10 mg/kg azoxymethane (AOM) and administered a 2.5% dextran sulfate sodium (DSS) solution (5 ml/day) for 7 days, which was repeated three times over 16 weeks. This study was approved by the Institutional Animal Care and Use Committee of Nanchang University (Nanchang, China).

### Patient samples

One hundred and twenty pairs of colorectal cancer tissues and corresponding adjacent normal tissues were collected from the First Affiliated Hospital of Nanchang University. The collection of clinical samples in this study was carried out in accordance with the requirements of the Medical Ethics Committee of the First Affiliated Hospital of Nanchang University (Nanchang, China). Patients included in the study were diagnosed with primary, sporadic colorectal cancer, and all provided written informed consent. To ensure the relevance of our findings, we excluded patients with inflammatory bowel disease, such as Crohn’s disease or ulcerative colitis, as well as those with hereditary colorectal cancer syndromes, including Lynch syndrome and familial adenomatous polyposis. Additionally, patients who had received preoperative chemoradiotherapy were excluded, as treatment could alter the molecular characteristics of both tumor and normal tissues.

### Colorectal cancer organoid culture

To establish mouse colorectal cancer organoids, colon adenomas were isolated from mice with AOM/DSS-induced colorectal cancer. To establish patient-derived colorectal cancer organoids, fresh surgically resected colorectal cancer tissues were placed in primary tissue transport medium (Advanced DMEM/F-12 supplemented with penicillin/streptomycin and 10 μM Y27632) and transported to the laboratory.

### Single-cell RNA sequencing analysis

Single-cell RNA sequencing (scRNA-seq) data were obtained from GSE132465 dataset in the Gene Expression Omnibus (GEO) database. A total of 25,655 genes and 63,689 cells were obtained from 23 colorectal cancer patients with 23 primary colorectal cancer samples and 10 matched normal mucosa samples. Quality control filters were applied to remove low-quality cells expressing fewer than 200 genes or exhibiting a mitochondrial gene content exceeding 20%, as well as potential doublets with feature counts greater than 6000. Genes detected in fewer than 3 cells were removed from subsequent analysis. Data normalization was performed using the “NormalizeData” and “ScaleData” functions in the “Seurat” R package. Batch effects across samples were corrected using the “Harmony” R package [49]. This reduction was applied to both the “RunUMAP” and “FindNeighbors” functions for clustering. Clustering analysis was conducted based on the first 18 principal components with a resolution parameter of 0.5. The cell subpopulations were annotated on the basis of established cell markers from published literature [50]. The “DimPlot,” “DotPlot,” and “VlnPlot” functions from the “Seurat” R package were used for visualization. Furthermore, density plots for *HOTTIP* expression were generated using the “plot_density” function from the “Nebulosa” R package.

### Statistical analysis

Differences in quantitative data between the two groups for real-time PCR, the luciferase assay, in vitro assays (such as the EdU assay, CCK-8, cell colony formation, and cell cycle analysis), and in vivo tumor weight were analyzed with a two-tailed unpaired *t* test. The significance of continuous cell growth curves and in vivo, tumor volume differences between groups was assessed using one-way ANOVA. The *χ²* test was used to analyze the correlation between gene expression and clinicopathological characteristics. All the statistical analyses were conducted using GraphPad Prism (GraphPad Software, Version 9).

## Results

### *HOTTIP* is a direct target of GLI2

**Fig. 1:**
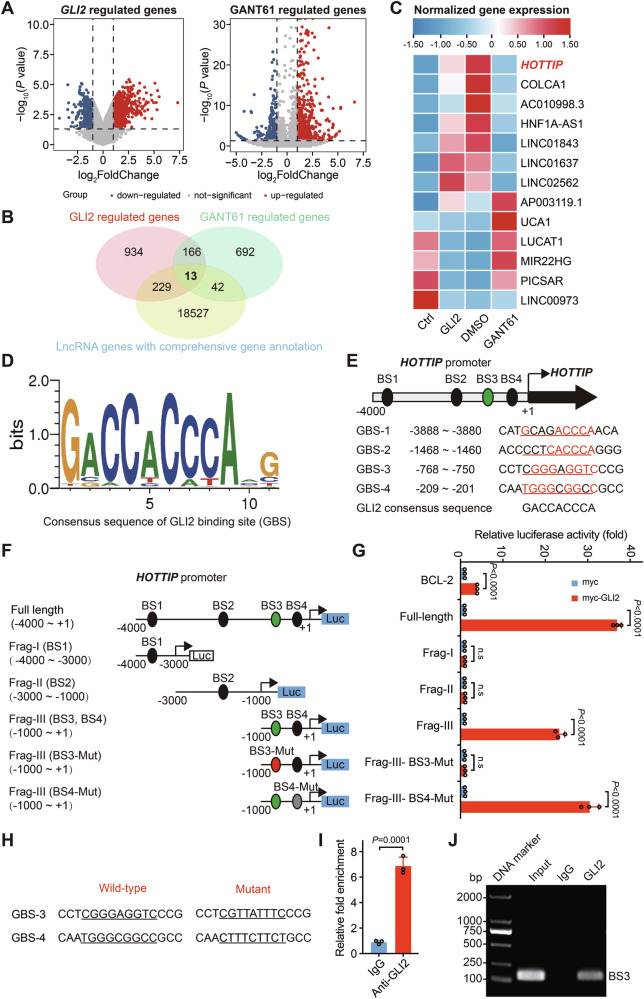
*HOTTIP* is a direct target of GLI2. **A** Volcano plots showing genes that were differentially expressed in HT-29 cells subjected to either GLI2 overexpression or GANT61 (10 μM) treatment for 48 h (fold change > 2 or < 0.5, adjusted *P* < 0.05). **B**, **C** Venn diagram and heatmap of differentially expressed lncRNAs in GLI2-overexpressing HT-29 cells and GANT61-treated HT-29 cells. **D** Schematic diagram showing the consensus sequence of the GLI2 binding site. **E** Schematic diagram of the *HOTTIP* promoter region with the conserved GLI2 binding site. The GBS core sequence is underlined, and the GBS consensus sequence is shown in red. **F** Schematic diagram showing the candidate GBSs within the *HOTTIP* promoter, the full-length luciferase reporter construct, and the corresponding fragments (Frag-I, II, III) with different GBSs. **G** A luciferase reporter vector containing the WT or mutated *HOTTIP* promoter was transfected in the presence or absence of GLI2 (*n* = 3 biologically independent samples). **H** Mutated GBS sequences in the Frag-III reporter constructs. **I**, **J** ChIP analysis results showing the binding of GLI2 to the *HOTTIP* promoter. Protein-bound chromatin was immunoprecipitated with an anti-GLI2 antibody, and IgG was used as a control. The immunoprecipitated DNA was analyzed via qPCR with primers targeting the *HOTTIP* promoter (**I**, *n* = 3 biologically independent samples), and the amplification products of the immunoprecipitated DNA were analyzed via agarose gel electrophoresis. The results were consistent with those of ChIP‒qPCR (**J**). The *P* values were calculated by a two-tailed unpaired *t* tests. The data are presented as the means ± SDs.

To identify lncRNAs regulated by Hh signaling, HT-29 colorectal cancer cells were treated with GANT61 (a GLI antagonist) or modified to overexpress GLI2. Deep RNA sequencing (RNA-seq) revealed 13 lncRNAs significantly regulated by GLI2 and GANT61 (Fig. 1A–C). Among these, *HOTTIP* was notable because of its conserved GLI-binding site (GBS). GLI2A overexpression increased *HOTTIP* expression, whereas GLI2 knockdown reduced it (Fig. S1A–D). Activating the Hh pathway with an SMO agonist (SAG) increased *HOTTIP* expression, whereas inhibiting cyclopamine or GANT61 reduced *HOTTIP* expression (Fig. S1E–J), indicating that *HOTTIP* is induced by Hh signaling.

To determine whether *HOTTIP* is a direct target gene of GLI2, we used JASPAR, which identified four potential GBSs in the promoter region of *HOTTIP* (Fig. 1D, E). These genomic sequences were subsequently cloned and inserted into the pGL4.20 basic vector for dual-luciferase assays, generating constructs encoding full-length, Frag-I, -II, and -III containing GBS 1, GBS 2, and GBS 3-4, respectively (Fig. 1F). In HEK-293T cells overexpressing GLI2A, the Frag-Full-length and Frag-III constructs exhibited significantly elevated luciferase activity (Fig. 1G). Frag-III contained both GBS 3 and GBS 4, and we individually mutated these sites and assessed luciferase activity (Fig. 1H). Since mutation of GBS 3 in Frag-III almost completely eliminates GLI2-induced luciferase activity, whereas mutation of GBS 4 does not, GBS 3 is likely essential for GLI2-mediated *HOTTIP* transcriptional activation (Fig. 1G). This observation was further supported by the results of a ChIP assay (Fig. 1I, J). These findings indicate that *HOTTIP* is an lncRNA that is induced by Hh signaling and is directly regulated by GLI2.

### *HOTTIP* overexpression promotes colorectal cancer cell proliferation

**Fig. 2:**
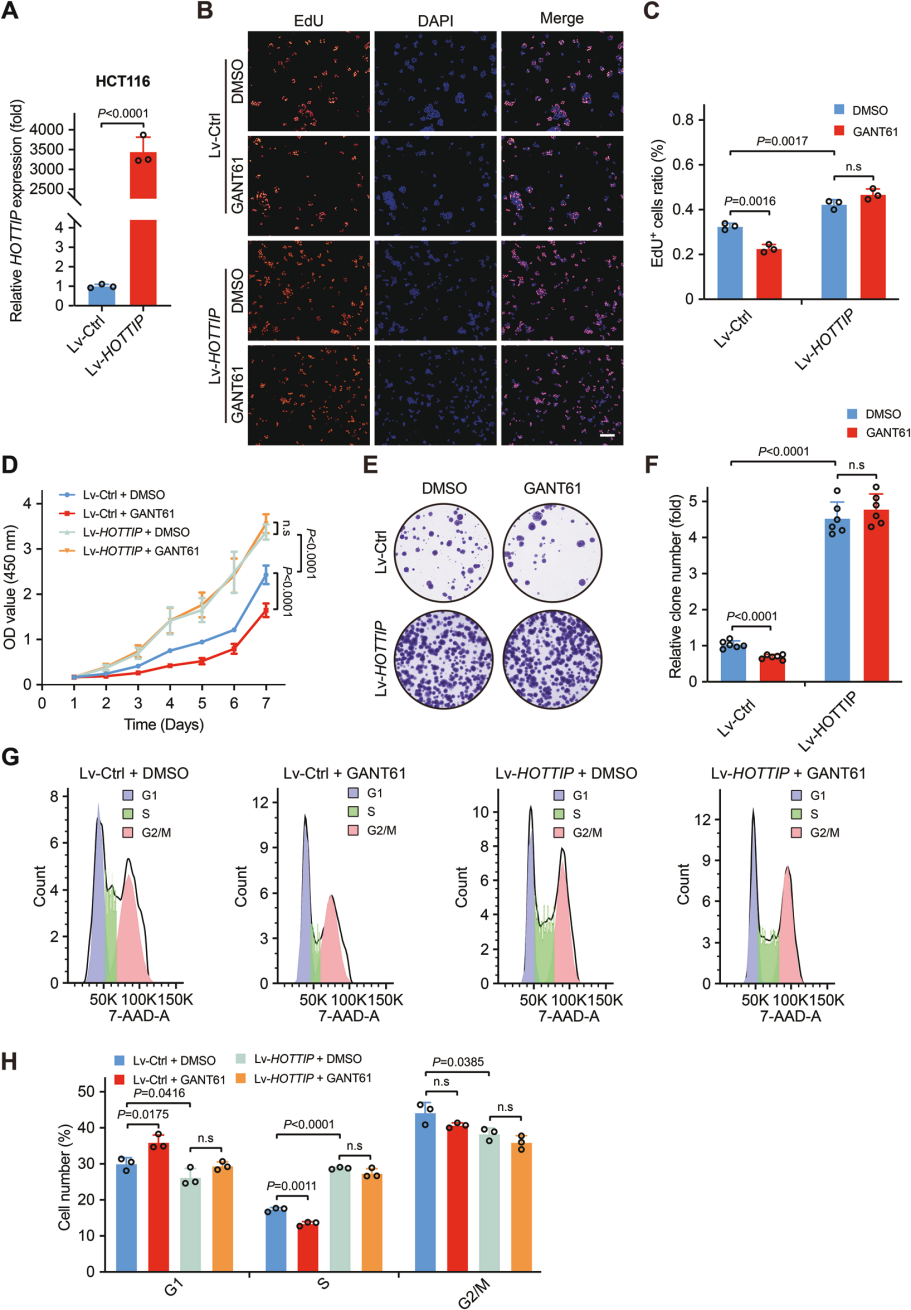
Overexpression of *HOTTIP* promotes colorectal cancer cell proliferation despite the inhibition of upstream Hh/GLI signaling. **A** qPCR analysis results showing the successful overexpression of *HOTTIP* in HCT116 cells. EdU incorporation assays of HCT116 cells in the Lv-Ctrl + DMSO, Lv-Ctrl + GANT61, Lv-*HOTTIP* + DMSO, and Lv-*HOTTIP* + GANT61 groups (**B**) and quantitative analysis of the proportion of EdU-positive cells (**C**, *n* = 3 biologically independent samples); scale bars, 100 μm. **D** CCK-8 assays of HCT116 cells in the Lv-Ctrl + DMSO, Lv-Ctrl + GANT61, Lv-*HOTTIP* + DMSO, and Lv-*HOTTIP* + GANT61 groups (*n* = 6 biologically independent samples). Colony formation assays of HCT116 cells in the Lv-Ctrl + DMSO, Lv-Ctrl + GANT61, Lv-*HOTTIP* + DMSO, and Lv-*HOTTIP* + GANT61 groups (**E**) and quantitative analysis of the cell colony number (**F**, *n* = 6 biologically independent samples). Cell cycle analysis of HCT116 cells in the Lv-Ctrl + DMSO, Lv-Ctrl + GANT61, Lv-*HOTTIP* + DMSO, and Lv-*HOTTIP* + GANT61 groups (**G**) and the fraction of cells in each phase (**H**, *n* = 3 biologically independent samples). The *P* values were calculated by ANOVA and two-tailed unpaired *t* tests. The data are presented as the means ± SDs.

*HOTTIP* has been identified as an oncogene that promotes cancer proliferation and tumorigenesis [29, 32]. To elucidate its biological effect on Hh/GLI signaling-dependent growth, HCT116 and SW620 cells were infected with lentiviruses for stable *HOTTIP* expression (Figs. 2A and S2A). Multiple assays, including EdU incorporation, CCK8, and colony formation assays, were used to test the effects of *HOTTIP* and Hh signaling on cell proliferation. Treatment with GANT61, a GLI antagonist, reduced the proportion of EdU-positive cells (Figs. 2B, C and S2B, C), viability (Figs. 2D and S2D), and colony formation ability (Figs. 2E, F and S2E, F), whereas *HOTTIP* overexpression accelerated cell growth (Figs. 2B–F and S2B–F). Notably, the inhibitory effects of GANT61 were significantly reduced with ectopic *HOTTIP* expression (Figs. 2B–F and S2B–F), suggesting that Hh signaling regulates cell proliferation through a *HOTTIP*-dependent pathway.

To investigate the effects of *HOTTIP* overexpression on cell cycle progression, we used employed imaging flow cytometry to analyze the cell cycle distribution. As shown in Figs. 2G, H and S2G, H, GANT61 treatment significantly increased the proportion of cells in the G1 phase and prevented cells from entering the S phase, thereby inhibiting cell proliferation. However, overexpression of *HOTTIP* significantly reduced the inhibitory effect of GANT61 on cell proliferation, with cells transitioning more rapidly from the G1 phase to the S phase. The number of S phase cells increased, which indicated a reduction in the cell cycle arrest induced by GANT61 and greater cell proliferation. These results indicate that *HOTTIP* plays a critical role in rescuing GANT61-induced proliferative defects and acts as a downstream effector of Hh/GLI signaling in colorectal cancer proliferation.

### Knockout or knockdown of *HOTTIP* inhibits Hh/GLI signaling-induced colorectal cancer cell proliferation

**Fig. 3:**
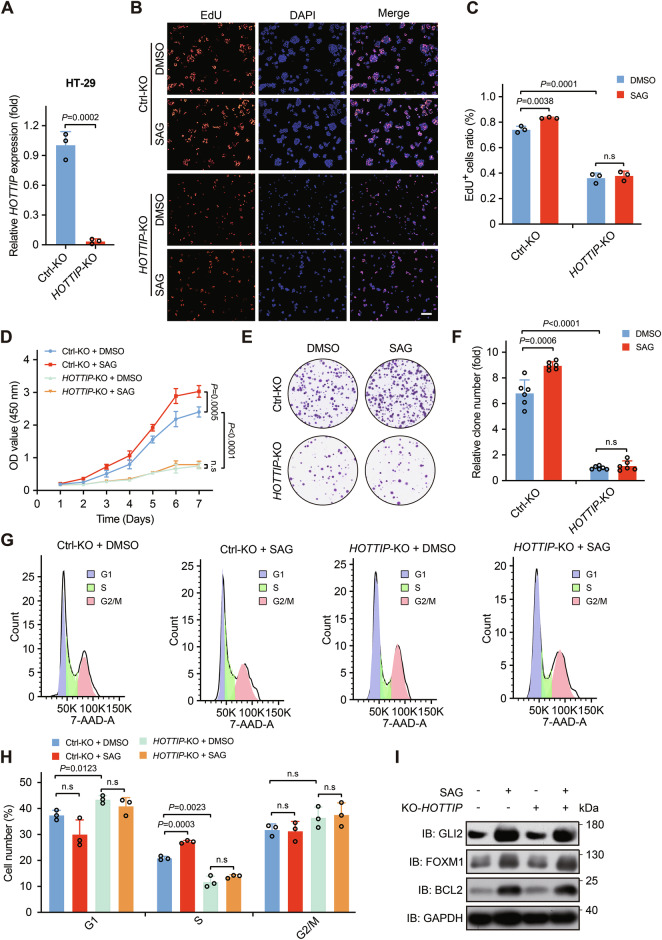
Knockout of *HOTTIP* inhibits Hh signaling-induced colorectal cancer cell proliferation. **A** qPCR analysis results showing the successful knockout of *HOTTIP* in HT-29 cells. EdU incorporation assays of HT-29 cells in the Ctrl-KO + DMSO, Ctrl-KO + SAG, *HOTTIP*-KO + DMSO, and *HOTTIP*-KO + SAG groups (**B**) and quantitative analysis of the proportion of EdU-positive cells (**C**, *n* = 3 biologically independent samples); scale bars, 100 μm. **D** CCK-8 assays of HT-29 cells in the Ctrl-KO + DMSO, Ctrl-KO + SAG, *HOTTIP*-KO + DMSO, and *HOTTIP*-KO + SAG groups (*n* = 6 biologically independent samples). Colony formation assays of HT-29 cells in the Ctrl-KO + DMSO, Ctrl-KO + SAG, *HOTTIP*-KO + DMSO, and *HOTTIP*-KO + SAG groups (**E**) and quantitative analysis of the cell colony number (**F**, *n* = 6 biologically independent samples). Cell cycle analysis of HT-29 cells in the Ctrl-KO + DMSO, Ctrl-KO + SAG, *HOTTIP*-KO + DMSO, and *HOTTIP*-KO + SAG groups (**G**) and the fraction of cells in each phase (**H**, *n* = 3 biologically independent samples). SAG promotes GLI2, FOXM1, and BCL2 expression, independent of *HOTTIP* knockout (**I**). The *P* values were calculated by ANOVA and two-tailed unpaired *t* tests. The data are presented as the means ± SDs.

Next, we investigated the effects of *HOTTIP* knockout and knockdown on Hh/GLI signaling-dependent colorectal cancer cell proliferation. *HOTTIP* knockout cells were generated via the transfection of CRISPR/Cas9 constructs into HT-29 cells, while DLD-1 cells were used for stable knockdown (Figs. 3A and S3A). *HOTTIP* knockout or knockdown decreased the proportion of EdU-positive cells (Figs. 3B, C and S3B, C) and reduced cell viability (Figs. 3D and S3D) and colony formation ability (Figs. 3E, F and S3E, F). The activation of Hh signaling by SAG promoted cell proliferation (Figs. 3B–F and S3B–F), whereas *HOTTIP* knockout or knockdown blocked this regulatory effect (Figs. 3B–F and S3B–F). Cell cycle assays indicated that SAG treatment significantly increased the proportion of cells in the S phase, reduced the number of cells in the G1 phase, and had no significant effect on the number of cells in the G2 phase, leading to increased cell proliferation. However, following *HOTTIP* knockout or knockdown, the proliferative effect of SAG on cell proliferation was weakened, with a significant reduction in the number of cells in the S phase, an increase in the number of cells in the G1 phase, and no significant change in the number of cells in the G2 phase (Figs. 3G, H and S3G, H). Additionally, while SAG promoted an increase in GLI2, FOXM1, and BCL2 protein levels, *HOTTIP* knockout did not affect their expression (Fig. 3I). Together, these results suggest that *HOTTIP* depletion significantly attenuates Hh/GLI signaling activation-mediated proliferation of colorectal cancer cells.

### *HOTTIP* represses p53 activity by promoting its HUWE1-induced proteasomal degradation

**Fig. 4:**
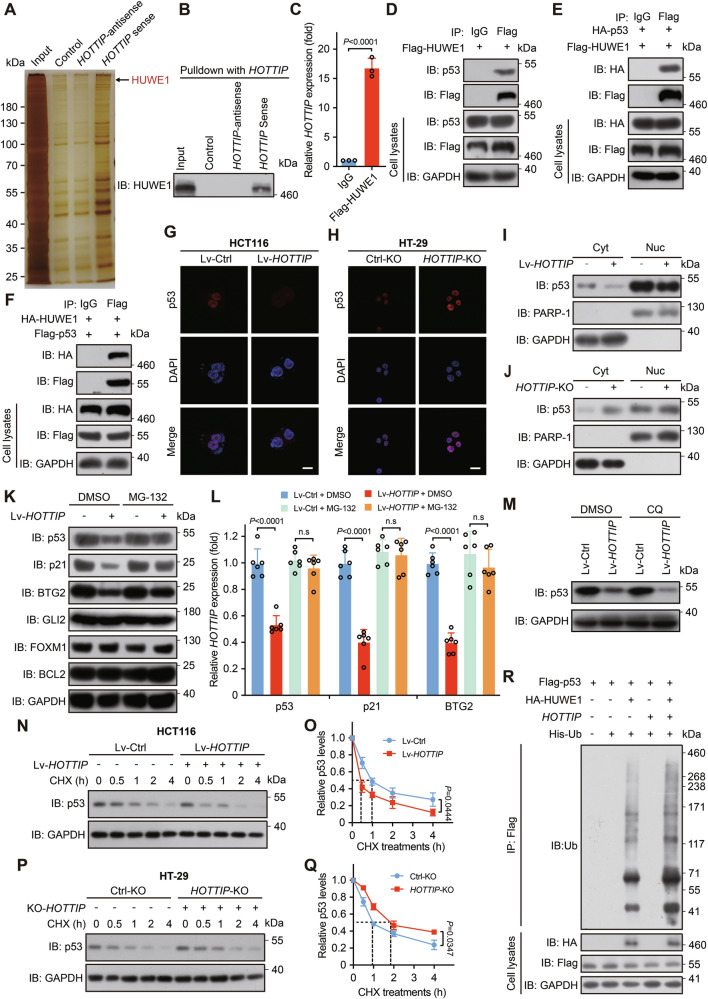
*HOTTIP* represses p53 activity by promoting HUWE1-induced proteasomal degradation of p53. **A** HUWE1 is a candidate interaction partner of *HOTTIP*. Biotinylated-sense *HOTTIP*, intronic control, and antisense *HOTTIP* probes were incubated with DLD-1 cell lysates, and the enriched proteins were eluted, separated via SDS‒PAGE, and detected by silver staining. **B** Flag-HUWE1 was pulled down with *HOTTIP*, and an RNA pulldown assay was then performed. **C** *HOTTIP* was enriched with Flag-HUWE1 in DLD-1 cell lysates, and a RIP assay was then performed. **D** Endogenous complexes of p53 with Flag-HUWE1. DLD-1 cells were transfected with a plasmid encoding Flag-HUWE1, and co-IP and Western blotting were performed. **E** Ectopically expressed p53 interacted with HUWE1. HEK-293T cells transfected with HA-p53 and Flag-HUWE1 plasmids were used for co-IP and Western blotting. **F** Ectopically expressed HUWE1 interacted with p53. HEK-293T cells transfected with HA-HUWE1 and Flag-p53 plasmids were used for co-IP and Western blotting assays. **G**, **I** Overexpression of *HOTTIP* reduced the p53 protein level in HCT116 cells; scale bars, 10 μm. **H**, **J** Knockout of *HOTTIP* increased the p53 protein level in HT-29 cells; scale bars, 10 μm. The proteasome inhibitor MG-132 but not the autophagy inhibitor CQ blocked *HOTTIP*-mediated p53 degradation in HCT116 cells. HCT116 cells were pretreated with MG-132 (**K**, **L**, 20 μM, 6 h) or CQ (**M**, 50 μM, 8 h) before being harvested for Western blotting. MG-132 treatment combined with *HOTTIP* overexpression altered p21 expression but did not affect the expression of GLI2, FOXM1, or BCL2. **N**, **O** The half-life of p53 was shortened upon *HOTTIP* overexpression. **P**, **Q** The half-life of p53 was extended upon *HOTTIP* depletion. HCT116 and HT-29 cells were treated with 100 μg/ml CHX and harvested at the indicated time points for Western blotting (**N**, **P**). **O**, **Q** p53/GAPDH ratio. **R** *HOTTIP* promoted HUWE1-mediated ubiquitination of p53. HEK-293T cells were transfected with combinations of plasmids encoding Flag-p53, HA-HUWE1, and *HOTTIP* as indicated and treated with MG-132 (20 μM, 6 h) before being harvested for in vivo ubiquitination assays. The *P* values were calculated by ANOVA and two-tailed unpaired *t* tests. The data are presented as the means ± SDs.

Fluorescence in situ hybridization (FISH) revealed that *HOTTIP* predominantly resides in the cytoplasm (Fig. S4). To elucidate the molecular mechanism of *HOTTIP* in colorectal cancer cells, we subsequently performed RNA pulldown with biotinylated *HOTTIP* in whole-cell lysates of DLD-1 cells to identify proteins that interact with *HOTTIP*. An intronic transcript and a *HOTTIP* antisense transcript were used as negative controls. The *HOTTIP*-associated proteins were then separated via gel electrophoresis and identified via mass spectrometry (MS) (Fig. 4A). Kyoto Encyclopedia of Genes and Genomes (KEGG) pathway analysis revealed that the significantly enriched pathways included ATP-dependent chromatin remodeling, the cell cycle, and ubiquitin-mediated proteolysis (Fig. S5A, B). We focused on five candidate proteins, including HUWE1, which mediates p53 ubiquitination (Fig. S5C). The binding between *HOTTIP* and HUWE1 was confirmed by RNA pulldown (Fig. 4B) and RNA immunoprecipitation (RIP) (Fig. 4C) assays. Because HUWE1 mediates p53 ubiquitination [47], we assessed whether *HOTTIP* influences p53 stability. Co-Immunoprecipitation (co-IP) assays verified the interaction between HUWE1 and p53 (Fig. 4D–F). The overexpression of *HOTTIP* decreased the mRNA expression of p53 target genes, including p21, BAX, PUMA, and BTG2 (Fig. S5D), whereas *HOTTIP* knockout in HT-29 cells increased the expression of these genes (Fig. S5E), indicating that *HOTTIP* modulates the posttranslational activity of p53. Ectopic expression of *HOTTIP* reduced p53 protein levels, whereas knockout increased p53 protein levels (Fig. 4G–J).

Further investigation revealed that *HOTTIP* promotes p53 degradation via the proteasomal pathway and that the proteasome inhibitor MG-132, but not the autophagy inhibitor CQ, blocks *HOTTIP*-mediated p53 degradation in HCT116 cells (Fig. 4K–M). A cycloheximide (CHX) chase assay revealed that ectopic expression of *HOTTIP* decreased but that knockout of *HOTTIP* extended the p53 protein half-life (Fig. 4N–Q). Additionally, *HOTTIP* overexpression reduced the expression of the p53 downstream proteins p21 and BTG2 but did not affect the key Hh signaling molecule GLI2 or its downstream target genes FOXM1 and BCL2 (Fig. 4K). Since HUWE1 is an E3 ubiquitin ligase for p53, we examined whether *HOTTIP* affects HUWE1-induced p53 ubiquitination. Although *HOTTIP* did not directly induce p53 ubiquitination, it promoted HUWE1-dependent ubiquitination and degradation (Fig. 4R). We also explored the role of MDM2 in the *HOTTIP*-HUWE1-p53 pathway in p53^−/−^ MDM2^−/−^ mouse embryonic fibroblasts (MEFs). Ectopic expression of HUWE1 and *HOTTIP* downregulated the expression level of p53 in p53^−/−^ MDM2^−/−^ MEFs (Fig. S5F), indicating that p53 can be degraded via the *HOTTIP*-HUWE1-p53 pathway independently of MDM2. Taken together, these results demonstrate that *HOTTIP* represses p53 activity by promoting its HUWE1-mediated proteasomal degradation.

### *HOTTIP* promotes colorectal cancer cell proliferation and tumor growth through inactivation of p53

**Fig. 5:**
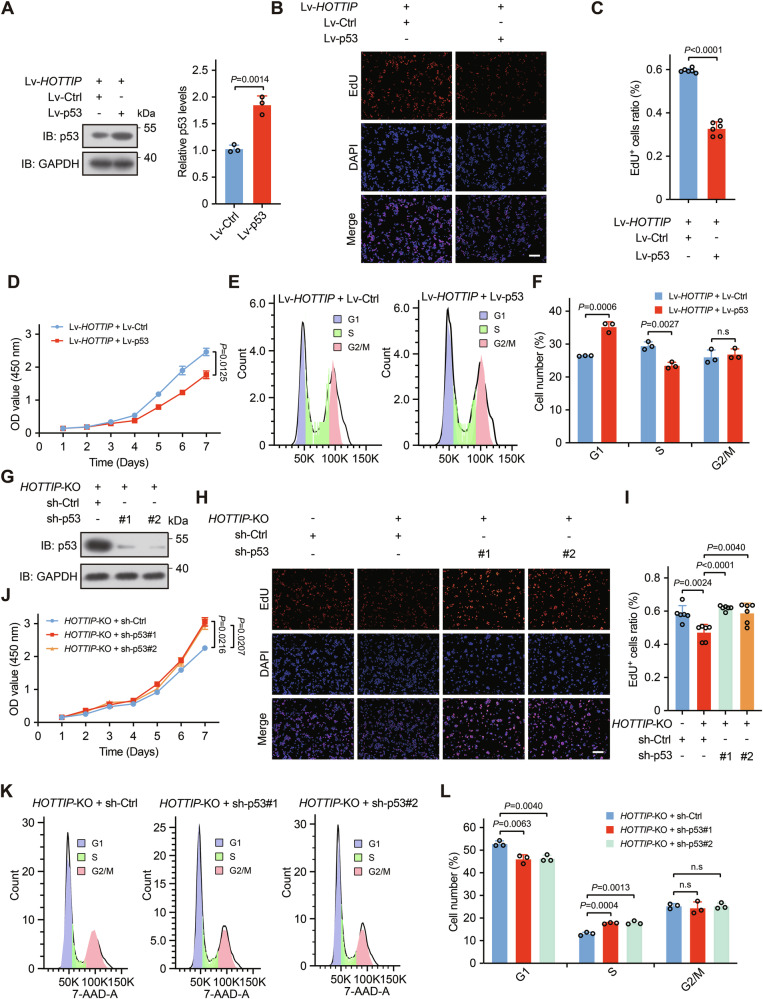
*HOTTIP* promotes colorectal cancer cell growth by inactivating p53. **A** Western blotting analysis results showing successful overexpression of p53 in HCT116 cells. EdU incorporation assays of Lv-*HOTTIP* + Lv-Ctrl and Lv-*HOTTIP* + Lv-p53 HCT116 cells (**B**) and quantitative analysis of the proportion of EdU-positive cells (**C**, *n* = 6 biologically independent samples); scale bars, 100 μm. **D** CCK-8 assays of Lv-*HOTTIP* + Lv-Ctrl and Lv-*HOTTIP* + Lv-p53 HCT116 cells (*n* = 3 biologically independent samples). Cell cycle analysis of Lv-*HOTTIP* + Lv-Ctrl, Lv-*HOTTIP* + Lv-p53 HCT116 cells (**E**) and the fraction of cells in each phase (**F**, *n* = 3 biologically independent samples). **G** Western blotting analysis results showing successful knockdown of p53 in HT-29 cells. **H**, **I** EdU incorporation assays of *HOTTIP*-KO + sh-Ctrl, *HOTTIP*-KO + sh-p53-1, and *HOTTIP*-KO + sh-p53-2 HT-29 cells and quantitative analysis of the proportion of EdU-positive cells (**I**, *n* = 6 biologically independent samples); scale bars, 100 μm. **J** CCK-8 assays of *HOTTIP*-KO + sh-Ctrl, *HOTTIP*-KO + sh-p53-1, and *HOTTIP*-KO + sh-p53-2 HT-29 cells (*n* = 3 biologically independent samples). Cell cycle analysis of *HOTTIP*-KO + sh-Ctrl, *HOTTIP*-KO + sh-p53-1, and *HOTTIP*-KO + sh-p53-2 HT-29 cells (**K**) and the fraction of cells in each phase (**L**, *n* = 3 biologically independent samples). The *P* values were calculated by ANOVA and two-tailed unpaired *t* tests. The data are presented as the means ± SDs.

To elucidate the epistatic relationship between *HOTTIP* and p53 in colorectal cancer proliferation, we overexpressed p53 in *HOTTIP*-overexpressing HCT116 cells (Fig. 5A) and conducted EdU incorporation assays, CCK8 assays, and cell cycle analysis. In *HOTTIP*-overexpressing cells, p53 overexpression decreased the proliferation driven by *HOTTIP* (Fig. 5B–D). In *HOTTIP*-overexpressing cells, p53 overexpression decreased the proliferation and S phase population driven by *HOTTIP*, leading to an increase in the G1 phase, a decrease in the S phase, and no change in the G2 phase, thereby reducing the positive effect of *HOTTIP* on cell proliferation and inhibiting cell proliferation (Fig. 5E, F). Conversely, p53 knockdown reversed the inhibitory effect of *HOTTIP* knockout on the proliferation of HT-29 cells (Fig. 5G–J). Conversely, when p53 was further downregulated in addition to *HOTTIP* downregulation, the number of G1 phase cells decreased, whereas the number of S phase cells increased, with no significant change in the G2 phase, thereby promoting cell proliferation (Fig. 5K, L).

To determine the biological function of *HOTTIP* in vivo, we established colorectal cancer xenograft models in mice using HCT116 cells with *HOTTIP* overexpression and HT-29 cells with *HOTTIP* knockout. The cells were injected subcutaneously into one flank of each nude mouse, and the tumor size was measured every three days. Ectopic *HOTTIP* expression resulted in significantly increased tumor volumes and weights, whereas *HOTTIP* knockout led to decreases in both tumor volumes and weights compared with those in the control groups (Fig. S6A–D). Next, the xenografts were harvested and collected for further analysis. *HOTTIP* overexpression was associated with the downregulation of both p53 and p21, whereas *HOTTIP* knockout increased their expression (Fig. S6E, F). Moreover, Ki-67 and PCNA staining revealed that ectopic expression of *HOTTIP* increased the levels of Ki-67 and PCNA, whereas *HOTTIP* knockout resulted in reduced Ki-67 and PCNA levels (Fig. S6G). These results indicate that *HOTTIP* may accelerate cell proliferation and tumor growth by regulating p53.

### *Hottip* promotes colorectal carcinogenesis in an AOM/DSS-induced mouse model

**Fig. 6:**
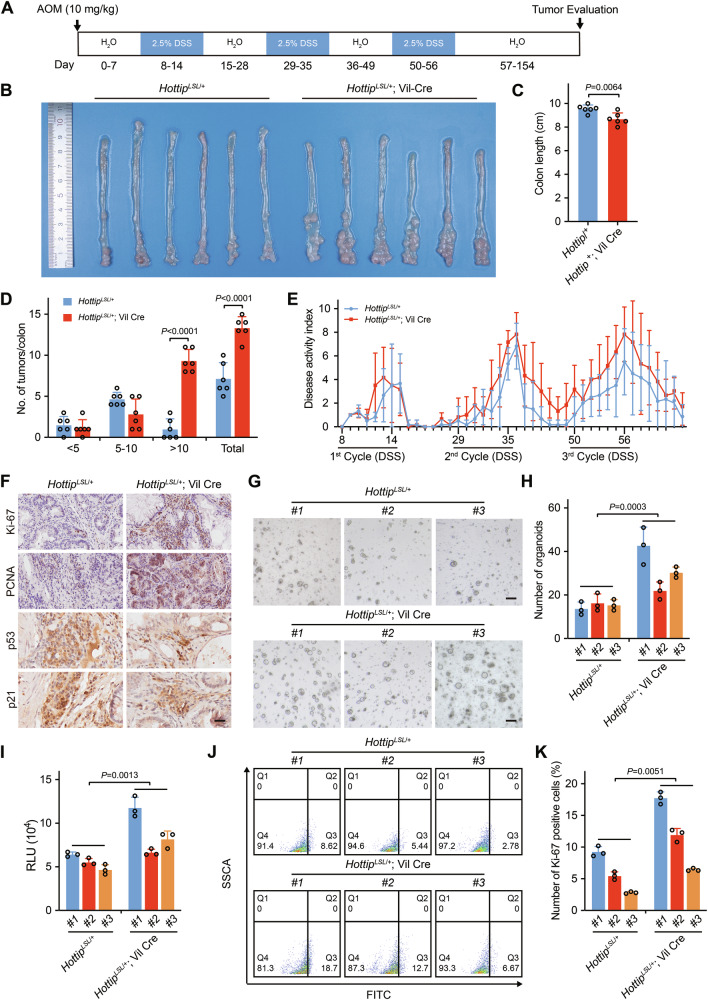
*Hottip* promotes colorectal carcinogenesis in an AOM/DSS-induced mouse model. **A** Schematic diagram of the experimental schedule. **B** Representative images of the colons and rectums from control (*Hottip*^*LSL*/+^) and *Hottip* KI (*Hottip*^*LSL*/+^; Vil-Cre) mice after AOM/DSS treatment, showing the extent of the tumor burden (*n* = 6 biologically independent animals). **C** The number and diameter of tumors in the entire colon and rectum of control (*Hottip*^*LSL*/+^) and *Hottip* KI (*Hottip*^*LSL*/+^; Vil-Cre) mice after AOM/DSS treatment were determined at the end of the study. **D** Average colon length in control (*Hottip*^*LSL*/+^) and *Hottip* KI (*Hottip*^*LSL*/+^; Vil-Cre) mice after AOM/DSS treatment. **E** DAI scores over the 3 cycles of DSS treatment. **F** Representative micrographs of immunochemical staining for Ki-67, PCNA, p53, and p21 in tumors from control (*Hottip*^*LSL*/+^) and *Hottip* KI (*Hottip*^*LSL*/+^; Vil-Cre) mice after AOM/DSS treatment; scale bars, 50 μm. Formation of organoids from crypts isolated from control (*Hottip*^*LSL*/+^) and *Hottip* KI (*Hottip*^*LSL*/+^; Vil-Cre) mice (**G**) and the number of crypt organoids formed (**H**, *n* = 3 biologically independent samples); scale bars, 200 μm. **I** The proliferation of organoids generated from crypts isolated from control (*Hottip*^*LSL*/+^) and *Hottip* KI (*Hottip*^*LSL*/+^; Vil-Cre) mice was evaluated by comparing the relative luminescence units (RLUs). The proliferation of organoids generated from crypts isolated from control (*Hottip*^*LSL*/+^) and *Hottip* KI (*Hottip*^*LSL*/+^; Vil-Cre) mice was evaluated via analysis of Ki-67 staining (**J**). Ki-67-positive cells were quantified (**K**, *n* = 3 biologically independent samples). The *P* values were calculated by a two-tailed unpaired *t* tests. The data are presented as the means ± SDs.

In mice, the *Hottip* gene is located on chromosome 6 (Fig. S7A). Interestingly, we found that *Hottip* is highly expressed in the large intestine (Fig. S7B). To investigate the role of the intestinal epithelial *Hottip*, we generated ROSA26 EGE-LYS-0150-A lncRNA knock-in mice via the EGE® system (Fig. S7C). Ectopic *Hottip* expression in the intestinal epithelium was driven by Villin-Cre (Vil-Cre), generating *Hottip*^LSL/+^; Vil-Cre mice, whereas mice lacking the Vil-Cre transgene served as controls (Fig. S7D–F). In this way, we established an AOM/DSS mouse model of colorectal cancer in which the administration of the mutagen AOM and the induction of chronic colitis with DSS may promote intestinal tumorigenesis (Fig. 6A). The AOM/DSS mouse model is an inflammation-driven colorectal cancer mouse model. Numerous studies have successfully used this model to elucidate key signaling pathways involved in colorectal cancer progression [51, 52]. After AOM/DSS treatment, the number and size of large intestinal tumors were markedly greater in *Hottip*^LSL/+^; Vil-Cre mice than in control mice, intriguingly, the colon length in *Hottip*^LSL/+^; Vil-Cre mice was shorter than that in control mice (Fig. 6B–D). The AOM/DSS-treated model mice presented obvious clinical characteristics of chronic colitis similar to those observed in humans with inflammatory bowel disease, such as body weight loss, diarrhea, and bloody stool, with a significant increase in the disease activity index (DAI) score over the 3 cycles of DSS treatment (Fig. 6E). *Hottip*^LSL/+^; Vil-Cre mice presented greater weight loss and more severe diarrhea and bloody stool, as indicated by their significantly higher DAI scores (Fig. 6E). Consistent with the findings in the xenograft model, Ki-67 and PCNA protein levels were increased in *Hottip*-expressing mice (Fig. 6F). Ectopic expression of *Hottip* led to reduced p53 and p21 protein levels in the tumor tissues (Fig. 6F). In addition, compared with those in the control group, we observed reduced expression of E-cadherin, increased expression of vimentin, β-catenin, elevated levels of VEGF, and an increase in the number of CD31^+^ microvessels in tumor tissues (Fig. S7G, H).

To further investigate the effect of *Hottip* on the proliferation of intestinal stem cells, we used an ex vivo culture system to assess the ability of isolated intestinal crypts to form organoids in 3D culture. Intestinal crypts were isolated from the large intestines of *Hottip*^LSL/+^ and *Hottip*^LSL/+^; Vil-Cre mice. A representative image of organoids generated from crypts isolated from 6 *Hottip*^LSL/+^ mice and *Hottip*^LSL/+^; Vil-Cre mice is shown (Fig. 6G). Compared with those from *Hottip*^LSL/+^ controls, the number and viability of organoids generated from *Hottip*^LSL/+^; Vil-Cre mice were greater (Fig. 6H, I). Flow cytometry analysis confirmed a marked increase in Ki-67-positive cells in organoids from *Hottip*^LSL/+^; Vil-Cre mice (Fig. 6J, K). Taken together, these results indicate that *Hottip* may promote colorectal cancer cell proliferation and tumor growth.

### *HOTTIP* promotes the growth of patient-derived tumor organoids

**Fig. 7:**
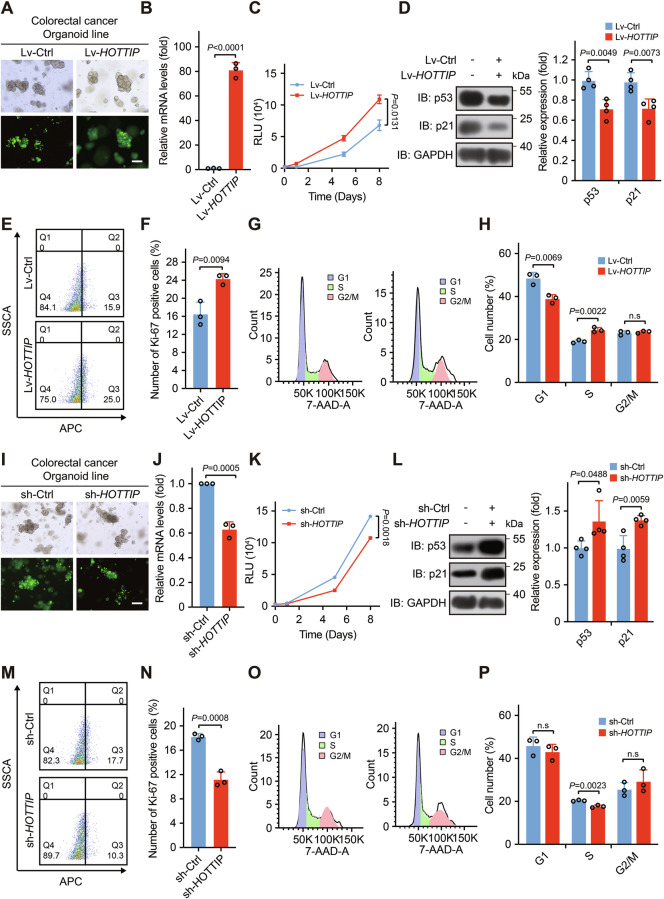
*HOTTIP* promotes the growth of patient-derived colorectal cancer organoids. **A** Bright-field images and immunofluorescence images of colorectal cancer organoids with stable *HOTTIP* overexpression; scale bars, 200 μm. **B** qPCR analysis of colorectal cancer organoids with stable *HOTTIP* overexpression. **C** Evaluation of the proliferation rate of colorectal cancer organoids stably expressing *HOTTIP*. **D** Overexpression of *HOTTIP* decreases p53 and p21 levels in colorectal cancer organoids. The proliferation of colorectal cancer organoids stably expressing *HOTTIP* was evaluated via analysis of Ki-67 staining (**E**), and Ki-67-positive cells were quantified (**F**, *n* = 3 biologically independent samples). Cell cycle analysis of colorectal cancer organoids stably expressing *HOTTIP* (**G**) and the fraction of cells in each phase (**H**, *n* = 3 biologically independent samples). **I** Bright field images and immunofluorescence images of colorectal cancer organoids with stable *HOTTIP* knockdown; scale bars, 200 μm. **J** qPCR analysis of *HOTTIP* knockdown in colorectal cancer cells. **K** Evaluation of the proliferation efficiency of colorectal cancer organoids upon *HOTTIP* knockdown. **L** Knockdown of *HOTTIP* increased p53 and p21 levels in colorectal cancer organoids. Proliferation of colorectal cancer organoids with stable *HOTTIP* knockdown via Ki-67 staining (**M**) and the proportion of Ki-67-positive cells (**N**, *n* = 3 biologically independent samples). Cell cycle analysis of colorectal cancer organoids with stable *HOTTIP* knockdown (**O**) and the fraction of cells in each phase (**P**, *n* = 3 biologically independent samples). The *P* values were calculated by a two-tailed unpaired *t* tests. The data are presented as the means ± SDs.

Patient-derived organoids, as emerging in vitro microphysiological systems, have demonstrated significant potential in biomedical research and precision medicine [53]. To perform the ex vivo studies of human colorectal cancer, we generated patient-derived organoids from tumor samples. We transduced organoids with a *HOTTIP*-overexpressing lentivirus (Fig. 7A), qPCR analysis confirmed successful *HOTTIP* overexpression (Fig. 7B). We assessed the viability of the colorectal cancer organoids via an ATP assay and found a dramatic increase in the viability of the *HOTTIP*-overexpressing colorectal cancer organoids (Fig. 7C). Given that *HOTTIP* promotes cancer cell growth by inhibiting the p53/p21 pathway, we evaluated the expression of p53 and p21, finding that *HOTTIP* overexpression reduced their levels (Fig. 7D). Ki-67 staining revealed an increased proportion of Ki-67-positive cells in *HOTTIP*-overexpressing organoids (Fig. 7E, F). Cell cycle analysis revealed that, with *HOTTIP* overexpression, the number of cells in the G1 phase decreased, the proportion of cells in the S phase increased, and there was no change in the number of cells in the G2 phase, thereby promoting cell proliferation (Fig. 7G, H).

Organoids were transduced with lentivirus expressing shRNA targeting *HOTTIP* (Fig. 7I), with knockdown efficiency confirmed by qPCR (Fig. 7J). ATP assays showed significantly decreased viability in *HOTTIP*-knockdown organoids (Fig. 7K). In contrast to overexpression results, *HOTTIP* knockdown significantly increased p53 and p21 expression (Fig. 7L). Ki-67 staining revealed decreased proportions of Ki-67-positive cells after knockdown (Fig. 7M–N). Cell cycle analysis indicated that *HOTTIP* knockdown significantly reduced S phase cells (with little effect on G1 and G2 phases), ultimately inhibiting proliferation (Fig. 7O–P). Together, these results suggest that *HOTTIP* promotes the growth of patient-derived tumor organoids.

### *HOTTIP* is highly expressed in human colorectal cancer tissues and predicts a poor clinical outcome

**Fig. 8:**
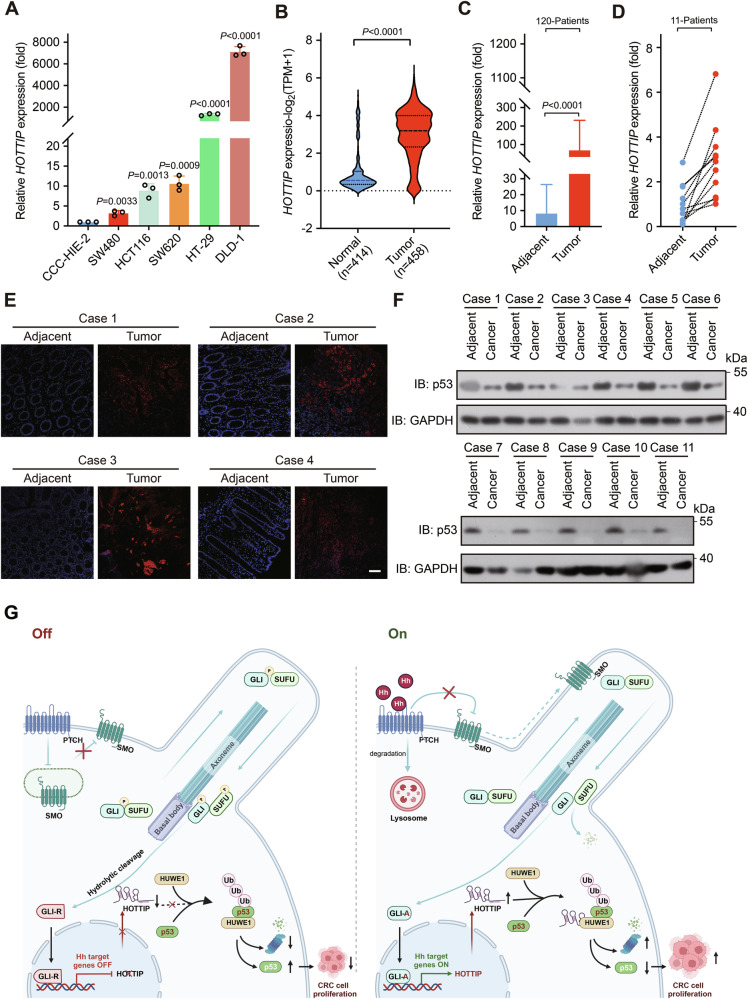
*HOTTIP* expression is high in colorectal cancer tumors, and high *HOTTIP* expression is correlated with poor survival in patients with colorectal cancer. **A** *HOTTIP* expression in colorectal cancer cell lines was measured by qPCR. A normal human colon epithelial cell line was used as the control. **B** *HOTTIP* expression levels in colorectal cancer clinical samples and normal tissue samples were evaluated by analysis of publicly available datasets (The Cancer Genome Atlas + Genotype-Tissue Expression colon cohort). **C** *HOTTIP* expression was higher in colorectal cancer tissues (*n* = 120) than in adjacent normal tissues. **D** *HOTTIP* expression was higher in colorectal cancer tissues (*n* = 11) than in matched adjacent normal tissues. **E** *HOTTIP* expression was greater in cancerous tissues than in adjacent normal tissues in a colorectal cancer tissue array, as determined by RNA in FISH; scale bars, 200 μm. **F** p53 protein expression was increased in colorectal cancer samples compared with adjacent normal tissues. Eleven pairs of tissues were analyzed via Western blotting. **G** Schematic diagram showing how lncRNA *HOTTIP* activation mediated by Hh signaling drives colorectal cancer malignant progression by promoting HUWE1-mediated ubiquitin‒proteasome degradation of p53. The *P* values were calculated by a two-tailed unpaired *t* tests. The data are presented as the means ± SDs.

We subsequently evaluated the expression of *HOTTIP* in 120 colorectal cancer samples and 11 randomly matched adjacent normal tissues. Compared with the human embryonic intestinal mucosa-derived cell line CCC-HIE-2, all five tested colorectal cancer cell lines (SW480, HCT116, SW620, HT-29, and DLD-1) presented increased expression of *HOTTIP* (Fig. 8A). In line with these results, analysis of The Cancer Genome Atlas and Genotype-Tissue Expression datasets revealed that *HOTTIP* expression was increased in 458 colorectal cancer samples compared with 414 normal colon samples (Fig. 8B). Consistent with the above findings, qPCR revealed significantly increased *HOTTIP* levels in cancer tissues (Fig. 8C). This difference was pronounced in the 11 random samples (Fig. 8D). To validate these results, we used a colorectal cancer tissue array and performed RNA FISH, confirming elevated *HOTTIP* levels in cancerous tissues compared with adjacent normal tissues (Fig. 8E). In the same 11 paired samples, *HOTTIP* expression was elevated, whereas p53 expression was reduced in the cancer tissues of all 11 pairs (Fig. 8F).

Furthermore, we analyzed the correlations between the expression of *HOTTIP* and the pathological features of colorectal cancer tissues. As shown in Table S10, the overexpression of *HOTTIP* was associated with poor differentiation, high TNM stage (III-IV), advanced T stage (T3-T4), advanced N stage (N1-N3), and vascular invasion. To further explore the implications of *HOTTIP* expression, we stratified the clinical data by stage, differentiation, and vascular invasion. The results revealed that in early TNM stages, *HOTTIP* expression was associated with tumor differentiation and vascular invasion (Table S11). In patients with well-differentiated and moderately differentiated tumors, *HOTTIP* expression was correlated with TNM stage and vascular invasion (Table S12). In patients without vascular invasion, *HOTTIP* expression was linked to TNM stage and tumor differentiation (Table S13). Additionally, we examined the correlation between *HOTTIP* and GLI2 expression and the pathological features of colorectal cancer tissues. The overexpression of GLI2 was linked to poor differentiation, advanced N stage (N1-N3), and vascular invasion (Table S14). Next, we analyzed the correlation between GLI2 and *HOTTIP* expression levels and found that, in all tumor patients, high GLI2 expression was associated with high *HOTTIP* expression. Further analysis by stratified analysis revealed that, in patients with TNM stages I-II across all differentiation grades and in those without vascular invasion, high GLI2 expression was correlated with high *HOTTIP* expression (Table S15). Finally, analysis of a colorectal cancer single-cell dataset (GSE132465) revealed high *HOTTIP* expression in the epithelial cells of tumor tissues, further supporting its role in cancer progression (Fig. S8). Moreover, VEGF, p53, and p21 were prominently detected in epithelial cells, whereas the endothelial marker CD31 was confined to endothelial cells. Notably, although GLI2 was clearly present in epithelial cells, it was more abundantly enriched in stromal cells (Fig. S8). Therefore, a comprehensive analysis of the spatial distribution and functional differences of the Hh signaling pathway across different cellular compartments and cell types in the intestine is crucial for understanding its biological role. Collectively, these results underscore the important role of *HOTTIP* in colorectal cancer progression and suggest that *HOTTIP* may serve as an independent prognostic biomarker.

## Discussion

In this study, we report that *HOTTIP* is a novel target of GLI transcription factors in response to Hh signaling activation. Our functional analyses of *HOTTIP* revealed that the overexpression of *HOTTIP* promoted cell proliferation and tumorigenesis in both a mouse model and a patient-derived organoid model. From a clinical perspective, elevated levels of *HOTTIP* were detected in colorectal cancer tissues and were strongly associated with an unfavorable prognosis in patients with colorectal cancer. On the basis of our results, we propose that *HOTTIP* acts as a positive regulator of Hh signaling. Mechanistically, in response to Hh signaling activation, *HOTTIP* binds to HUWE1 and promotes its E3 ubiquitin ligase activity toward p53, mediating HUWE1-dependent proteasomal turnover of p53, which decreases the level and activity of p53 and results in the expression of p53 pathway downstream target genes important for cell proliferation and survival (Fig. 8G).

To date, only a few lncRNAs have been reported to be related to the Hh signaling pathway. For example, h-Hilnc is regulated by Hh signaling and GLI1 and influences lipid accumulation [23]. Aberrant Hh signaling induces the expression of Yap1 and H19 during the development of osteoblastic osteosarcoma [54]. *LncHDAC2* recruits the NuRD complex to the promoter of the Ptch1 gene, which blocks Ptch1 expression and consequently activates Hh signaling to initiate liver tumorigenesis [55]. Our study also revealed that Hh signaling regulates the expression of *HOTTIP* by directly targeting the GLI2-binding site in the *HOTTIP* promoter region. Thus, the diverse array of transcription factor-binding sites in the regulatory regions of lncRNAs may play important roles in linking lncRNA expression to signaling activity.

Recent studies have shown that lncRNAs participate in cancer initiation and progression [56–58]. Mechanistically, lncRNAs can act as signal RNAs to regulate transcription in response to various stimuli; they can act as decoy RNAs to limit the availability of regulatory factors; they can act as scaffold RNAs to provide platforms for the assembly of protein complexes for transcription; they can act as guide RNAs for the proper localization of ribonucleoproteins; and they can act as enhancer RNAs for chromatin interactions [59]. Recently, aberrant upregulation of *HOTTIP* has been observed in various types of cancer and found to be correlated with poor survival in cancer patients [28, 29]. Because we found that Hh signaling activates *HOTTIP*, which in turn induces colorectal cancer cell proliferation (Figs. 2–3 and S2–3), we hypothesize that *HOTTIP* may also promote colorectal cancer progression. Our analyses of an array containing 120 pairs of primary colorectal cancer tissues and data from patients clearly demonstrated that the overexpression of *HOTTIP* in colorectal cancer patients was associated with poor differentiation, a high TNM stage (III-IV), an advanced T stage (T3-T4), and the presence of vascular invasion (Table S10). The viability of colorectal cancer organoids with *HOTTIP* overexpression was dramatically increased (Fig. 7). Furthermore, single-cell RNA sequencing analysis revealed significantly elevated *HOTTIP* expression in tumor epithelial cells, further supporting its protumorigenic role (Fig. S8). Our data presented herein suggest that *HOTTIP* may be a diagnostic and prognostic biomarker for colorectal cancer.

p53 is an important tumor suppressor gene. Activated p53 drives a broad transcriptional program that induces cell cycle arrest, promotes the activity of repair pathways, and, in response to severe stress, activates apoptosis. Under physiological conditions, maintaining a low intracellular level of p53 is critical, and this is achieved through the rapid proteasomal degradation of p53. This degradation is mediated via both ubiquitin-dependent and ubiquitin-independent mechanisms and can be modulated by various posttranslational modifications. Among these, ubiquitination is the most crucial, with the E3 ligase MDM2 serving as the primary negative regulator of p53. Additionally, E3 Ub ligases such as Cop1, TRIM32, and HUWE1 have been shown to target p53 for degradation through ubiquitination [47, 60, 61]. Accumulating evidence suggests that lncRNAs function as protein scaffolds, forming ribonucleoproteins and bringing proteins into physical proximity. For example, the lncRNA NRON scaffolds the E3 ubiquitin ligases MDM2 and MDMX via two different stem loops, thereby promoting the E3 ligase activity of MDM2 toward its tumor-suppressing substrates, including p53, RB1, and NFAT1 [48]. However, few scaffolding lncRNAs have been characterized, and the prevalence of this function remains unknown. Through RNA pulldown and mass spectrometry analysis, we found that *HOTTIP* functions as a scaffold for HUWE1, promoting its E3 ligase activity toward p53. Our studies revealed three new factors, *HOTTIP*, HUWE1, and p53, as important positive regulators of the Hh signaling pathway.

Previous studies have shown that sporadic colorectal cancer is caused by the accumulation of mutations in oncogenes and tumor suppressor genes; some of these mutations lead to aberrant activation of β-catenin. Mutations in APC, β-catenin, or other components of this pathway mediate the transition of single preneoplastic cells to aberrant crypt foci and then to adenoma and colorectal carcinoma. Chronic inflammation, which leads to colitis-associated cancer, is characterized by the production of proinflammatory cytokines that can induce mutations in oncogenes and tumor suppressor genes (APC, p53, and KRAS) and genomic instability via various mechanisms. Persistent inflammation facilitates tumor promotion by activating the proliferation and antiapoptotic properties of premalignant cells, as well as tumor progression and metastasis. There is considerable overlap in the mechanisms of sporadic colorectal cancer and colitis-associated cancer pathogenesis. Interestingly, APC, KRAS, and p53 mutations have been associated with colitis-related colorectal cancer, albeit less frequently than sporadic colorectal cancer [62, 63]. The AOM/DSS mouse model is an inflammation-driven colorectal cancer mouse model. The treatment of mice with DSS has been used to mimic chronic inflammation in the colon. Multiple rounds of DSS in drinking water induce sessile lesions and rates of dysplasia and adenocarcinoma similar to those observed in ulcerative colitis patients. AOM, a carcinogen, was added to this model to improve the efficiency of dysplasia and adenocarcinoma development. AOM with DSS induces stabilization and nuclear translocation of β-catenin by mutating exon 3 of the Ctnnb1 gene [64, 65], as well as by causing common molecular changes in colorectal cancer, such as upregulation of COX-2 and iNOS and loss of p53 [64]. While the AOM/DSS model is instrumental in studying the role of inflammation in colorectal cancer, it has limitations in fully recapitulating the genetic landscape and etiology of sporadic colorectal cancer. Additionally, it lacks the complete adenoma‒carcinoma sequence found in sporadic colorectal cancer.

Another mouse model of colorectal cancer utilizes mutations in the APC gene. APC sequesters β-catenin in the cytoplasm, thus preventing the nuclear translocation of β-catenin and the transcription of proproliferative genes in the Wnt signaling pathway, such as CCND1 and MYC [66]. Mutations in APC are also commonly found in sporadic colorectal cancers [67]. Interestingly, these mice presented a small intestinal phenotype, whereas humans with APC mutations develop adenomas in the colon. In this study, we investigated the role and molecular mechanism of the Hh signaling pathway in the carcinogenesis of colorectal cancer via an AOM/DSS mouse model. The relevance of this model was further enhanced by integrating a patient-derived colorectal cancer organoid model and clinical data, which helped address the model’s limitations and improved the overall applicability of the study to colorectal cancer pathogenesis (Fig. 7 and Tables S10–15). Therefore, our studies have revealed a novel molecular mechanism by which Hh signaling regulates the malignant progression of colorectal cancer *via* the Hh-*HOTTIP*-p53 signaling axis.

Previous studies have shown that activation of the Hh pathway is a key factor in maintaining the regeneration and repair of intestinal mucosal epithelial cells and the growth and metastasis of colorectal cancer [68, 69]. The activation of the Hh pathway has a tumor-promoting effect on colorectal cancer and may be involved in the maintenance of tumor stem cells [21, 70]. It has also been reported that stroma-specific Hh activation reduces the tumor load and blocks the progression of advanced neoplasms via the modulation of BMP signaling and restriction of the colonic stem cell signature [71]. Additionally, Hh signaling inhibits inflammatory damage in the intestine by inducing the expression of IL-10 in colorectal stromal cells [72]. Therefore, it will be necessary to clarify the different roles of Hh signaling in the spatial relationships between different cell compartments and different cell types in the intestine. Single-cell sequencing methods, spatial transcriptome techniques, and multiple immunofluorescence techniques will provide opportunities for future research.

In summary, our comprehensive basic and clinical studies described here reveal three new factors, *HOTTIP*, HUWE1, and p53, in the Hh signaling pathway. From a biochemical perspective, *HOTTIP* functions as a novel Hh target and effector, binding to the E3 ligase HUWE1 to mediate the ubiquitination and degradation of p53. From a biological perspective, high expression of *HOTTIP* can promote colorectal cancer cell proliferation and survival as well as tumorigenesis. From a clinical perspective, high levels of *HOTTIP* are strongly correlated with poor prognosis in patients with colorectal cancer. These findings reveal a critical role of the Hh-*HOTTIP*-p53 signaling axis in tumor progression and suggest a potential therapeutic target for colorectal cancer.

## Supplementary information

Supplementary Information.

Supplemental Material

## Data Availability

The full-length *HOTTIP* promoter sequence and the predicted binding sites of GLI2 were obtained from the JASPAR database [https://jaspar.elixir.no/]. The RNA-seq data generated in this study are available at the Sequence Read Archive with accession number PRJNA1132365. One published dataset was reanalyzed in the study (accession no. PRJNA623247) [73]. The raw microarray data are available at GEO (GSE132465). Other data are available in the manuscript or the supplementary materials.
