# Supplementary material for: LncRNA HOTTIP modulated by Hedgehog signaling drives colorectal cancer progression by promoting HUWE1-mediated ubiquitin‒proteasome degradation of p53
Source: Cell Death Dis. 2025 Jul 7;16(1):502. doi: 10.1038/s41419-025-07817-4 (PMC12234969; doi:10.1038/s41419-025-07817-4)
Supplement: Supplementary file 1 — Supplementary Information. [file 41419_2025_7817_MOESM1_ESM.pdf]

Supplementary Information

LncRNA *HOTTIP* modulated by Hedgehog signaling drives colorectal cancer progression by promoting HUWE1-mediated ubiquitin–proteasome degradation of p53

Hui Wang[1,2,#], Weiwei Jiao[3,#], Defu Li[4], Zhengping Yu[1,2], Huqiao Luo[5], Jie Zhang[1,2], Yuanbing Zhang[1,2], Hai Rao[6], Quqin Lu[4,*] & Bing Zhao[1,*] & Shiwen Luo[1,2,*]

[1] Center for Experimental Medicine, The First Affiliated Hospital of Nanchang University; The MOE Basic Research and Innovation Center for the Targeted Therapeutics of Solid Tumors; Jiangxi Medical College, Nanchang University, Nanchang, Jiangxi 330006, China.

[2] Department of Pathology and Institute of Molecular Pathology, Jiangxi Provincial Key Laboratory for Precision Pathology and Intelligent Diagnosis, The First Affiliated Hospital of Nanchang University, Nanchang, Jiangxi 330006, China.

[3] State Key Laboratory of Reproductive Regulation and Breeding of Grassland Livestock, Institute of Biomedical Sciences, School of Life Sciences, Inner Mongolia University, Hohhot, China.

[4] Department of Epidemiology and Biostatistics, Jiangxi Provincial Key Laboratory of Disease Prevention and Public Health, School of Public Health, Jiangxi Medical College, Nanchang University, Nanchang, Jiangxi 330006, China.

[5] School of Medicine, Shanghai University, Shanghai 200444, China.

[6] Department of Biochemistry, School of Medicine, Southern University of Science and Technology, Shenzhen 518055, China.

[#] These authors contributed equally: Hui Wang, Weiwei Jiao.

* Corresponding author: shiwenluo@ncu.edu.cn
bingzhao@biogenous.cn
quqinlu@ncu.edu.cn

Supplementary Figures

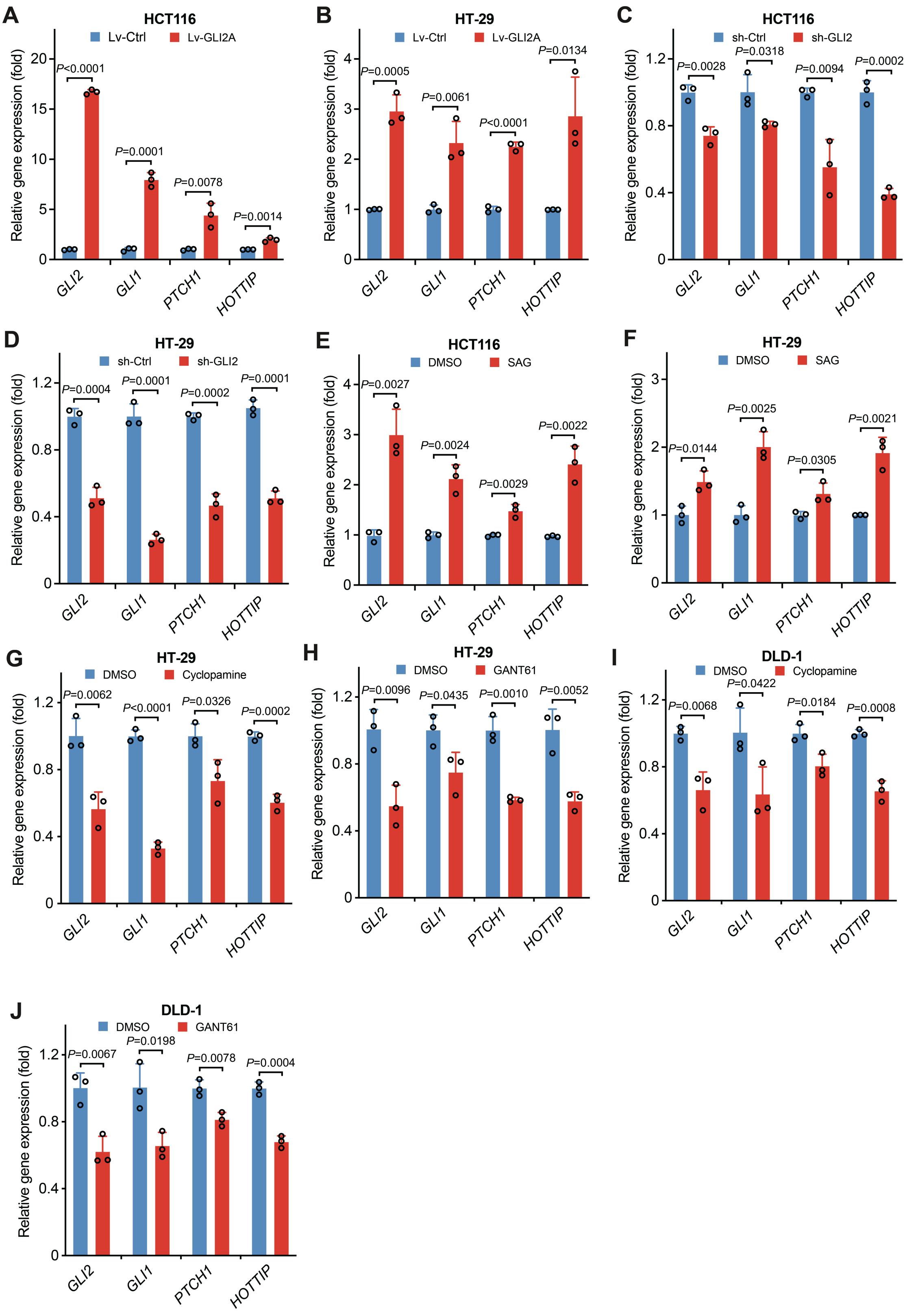

Supplementary Fig. 1 *HOTTIP* expression is regulated by the Hh signaling pathway. A Relative expression of GLI2, GLI1, PTCH1 and *HOTTIP* in HCT116 cells overexpressing GLI2 (*n* = 3 independent experiments). **B** Relative expression of GLI2, GLI1, PTCH1 and *HOTTIP* in HT-29 cells overexpressing GLI2 (*n* = 3 independent experiments). **C** Relative expression of GLI2, GLI1, PTCH1 and *HOTTIP* in GLI2-knockdown HCT116 cells (*n* = 3 independent experiments). **D** Relative expression of GLI2, GLI1, PTCH1 and *HOTTIP* in HT-29 cells with GLI2 knockdown (*n* = 3 independent experiments). **E** Relative expression of GLI2, GLI1, PTCH1 and *HOTTIP* in HCT116 cells after SAG (200 nM) treatment (*n* = 3 independent experiments). **F** Relative expression of GLI2, GLI1, PTCH1 and *HOTTIP* in HT-29 cells after SAG treatment (*n* = 3 independent experiments). **G** Relative expression of GLI2, GLI1, PTCH1 and *HOTTIP* in HT-29 cells after cyclopamine (20 μM) treatment (*n* = 3 independent experiments). **H** Relative expression of GLI2, GLI1, PTCH1 and *HOTTIP* in HT-29 cells after GANT61 treatment (*n* = 3 independent experiments). **I** Relative expression of GLI2, GLI1, PTCH1 and *HOTTIP* in DLD-1 cells after cyclopamine treatment (*n* = 3 independent experiments). **J** Relative expression of GLI2, GLI1, PTCH1 and *HOTTIP* in DLD-1 cells after GANT61 treatment (*n* = 3 independent experiments). The *P* values were calculated by a two-tailed unpaired *t* tests. The data are presented as the means ± SDs.

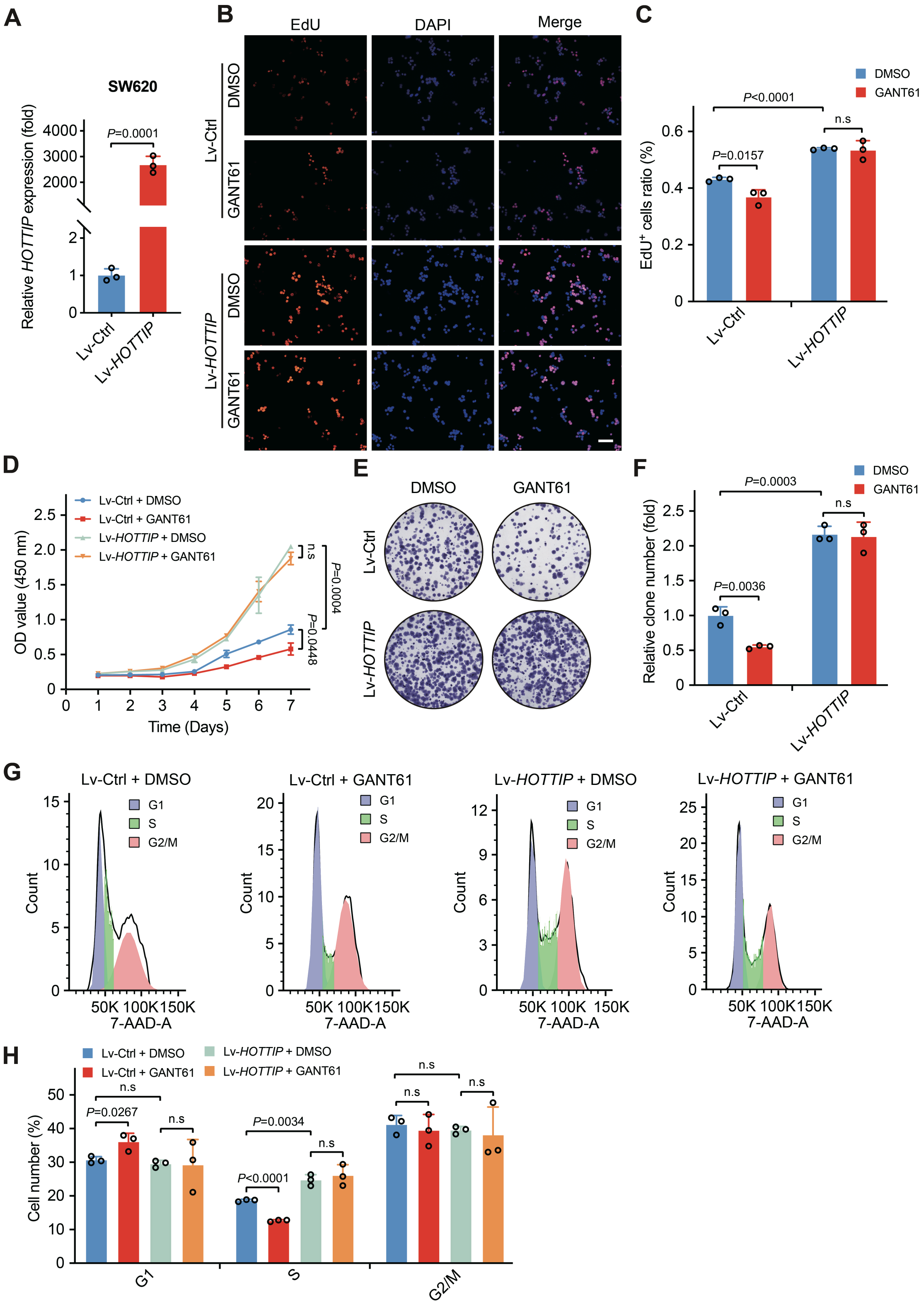

Supplementary Fig. 2 *HOTTIP* overexpression promotes colorectal cancer cell proliferation despite the inhibition of upstream Hh/GLI signaling. A

qPCR analysis results showing the successful overexpression of *HOTTIP* in SW620 cells. **B**, **C** EdU incorporation assays of SW620 cells in the Lv-Ctrl + DMSO, Lv-Ctrl + GANT61, Lv-*HOTTIP* + DMSO, and Lv-*HOTTIP* + GANT61 groups (**B**) and quantitative analysis of the proportion of EdU-positive cells (**C**, *n* = 3 biologically independent samples); scale bars, 100 μm. **D** CCK-8 assays of SW620 cells in the Lv-Ctrl + DMSO, Lv-Ctrl + GANT61, Lv-*HOTTIP* + DMSO, and Lv-*HOTTIP* + GANT61 groups (*n* = 3 biologically independent samples). **E**, **F** Colony formation assays of SW620 cells in the Lv-Ctrl + DMSO, Lv-Ctrl + GANT61, Lv-*HOTTIP* + DMSO, and Lv-*HOTTIP* + GANT61 groups (**E**) and quantitative analysis of the cell colony number (**F**, *n* = 3 biologically independent samples). **G**, **H** Cell cycle analysis of SW620 cells in the Lv-Ctrl + DMSO, Lv-Ctrl + GANT61, Lv-*HOTTIP* + DMSO, and Lv-*HOTTIP* + GANT61 groups (**G**) and the fraction of cells in each phase (**H**, *n* = 3 biologically independent samples). The *P* values were calculated by ANOVA and two-tailed unpaired *t* tests. The data are presented as the means ± SDs.

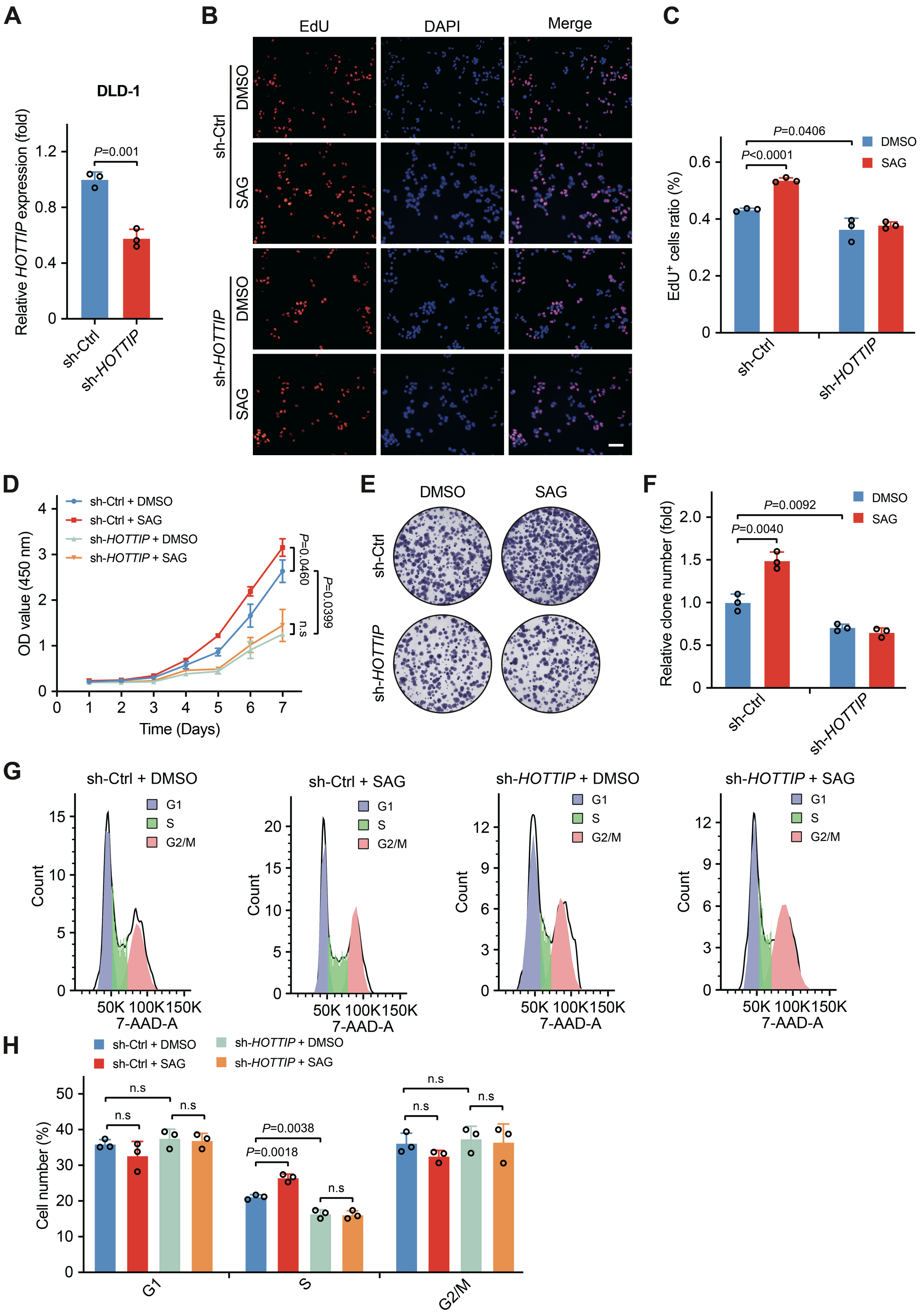

Supplementary Fig. 3 Knockdown of *HOTTIP* inhibits Hh signaling-induced colorectal cancer cell proliferation. A qPCR analysis results

showing the successful knockdown of *HOTTIP* in DLD-1 cells. **B**, **C** EdU incorporation assays of DLD-1 cells in the sh-Ctrl + DMSO, sh-Ctrl + SAG, sh-*HOTTIP* + DMSO, and sh-*HOTTIP* + SAG groups (**B**) and quantitative analysis of the proportion of EdU-positive cells (**C**, *n* = 3 biologically independent samples); scale bars, 100 μm. **D** CCK-8 assays of DLD-1 cells in the sh-Ctrl + DMSO, sh-Ctrl + SAG, sh-*HOTTIP* + DMSO, and sh-*HOTTIP* + SAG groups (*n* = 3 biologically independent samples). **E**, **F** Colony formation assays of DLD-1 cells in the sh-Ctrl + DMSO, sh-Ctrl + SAG, sh-*HOTTIP* + DMSO, and sh-*HOTTIP* + SAG groups (**E**) and quantitative analysis of the cell colony number (**F**, *n* = 3 biologically independent samples). **G**, **H** Cell cycle analysis of DLD-1 cells in the sh-Ctrl + DMSO, sh-Ctrl + SAG, sh-*HOTTIP* + DMSO, and sh-*HOTTIP* + SAG DLD-1 groups (**G**) and the fraction of cells in each phase (**H**, *n* = 3 biologically independent samples). The *P* values were calculated by ANOVA and two-tailed unpaired *t* tests. The data are presented as the means ± SDs.

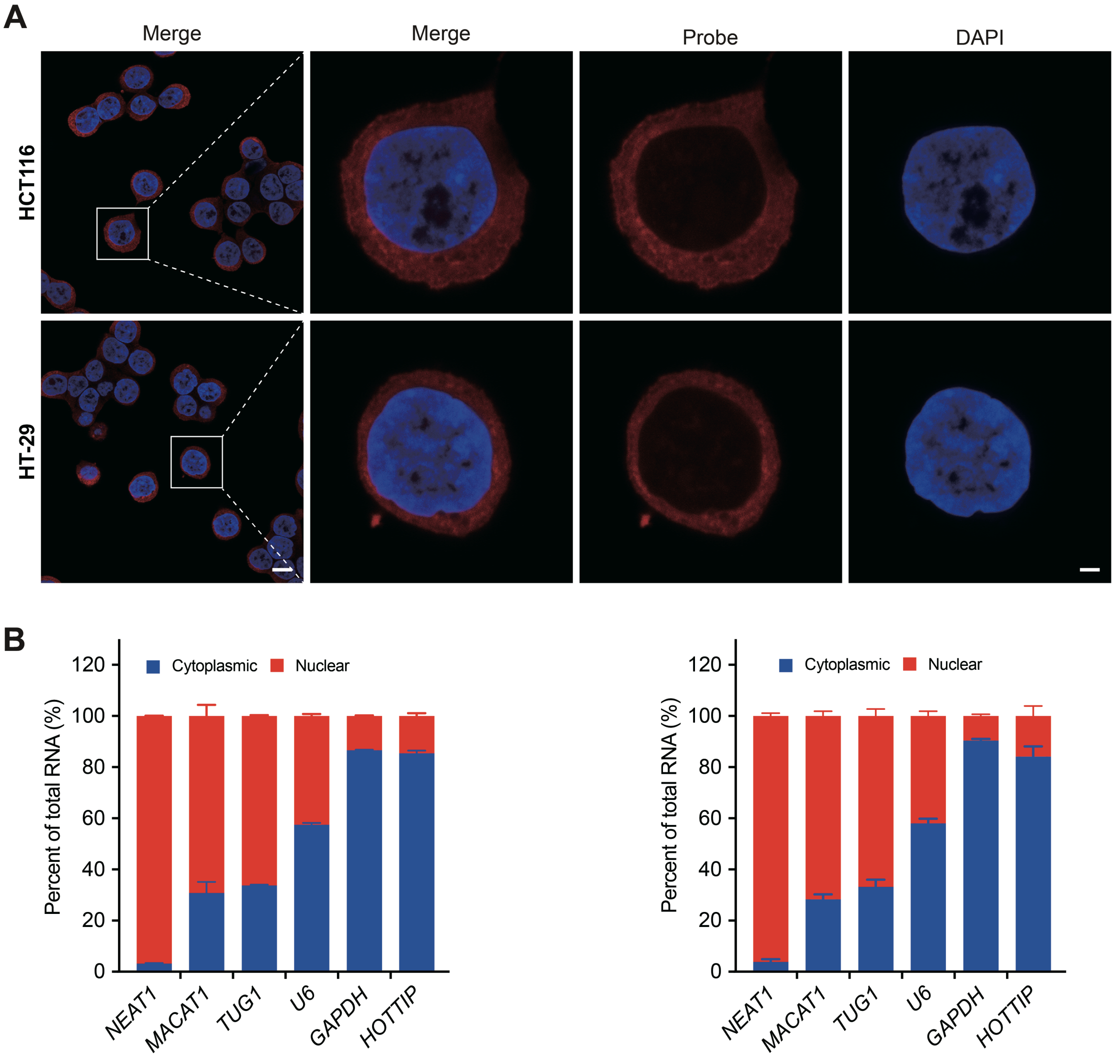

Supplementary Fig. 4 *HOTTIP* is localized primarily in the cytoplasm. A FISH analysis of *HOTTIP* in HCT116 and HT-29 cells. (Nuclei were stained with DAPI). scale bars, 50 μm (left), 10 μm (right). **B** Nuclear/cytoplasmic fractionation and qPCR were used to determine the cellular distribution of *HOTTIP* in HCT116 and HT-29 cells (n = 3 biologically independent samples). NEAT1, TUG1, MALAT1, BIRC5, U6 and GAPDH were used as fractionation quality standards and endogenous controls. The P values were calculated by a two-tailed unpaired t tests. The data are presented as the means ± SDs.

A B

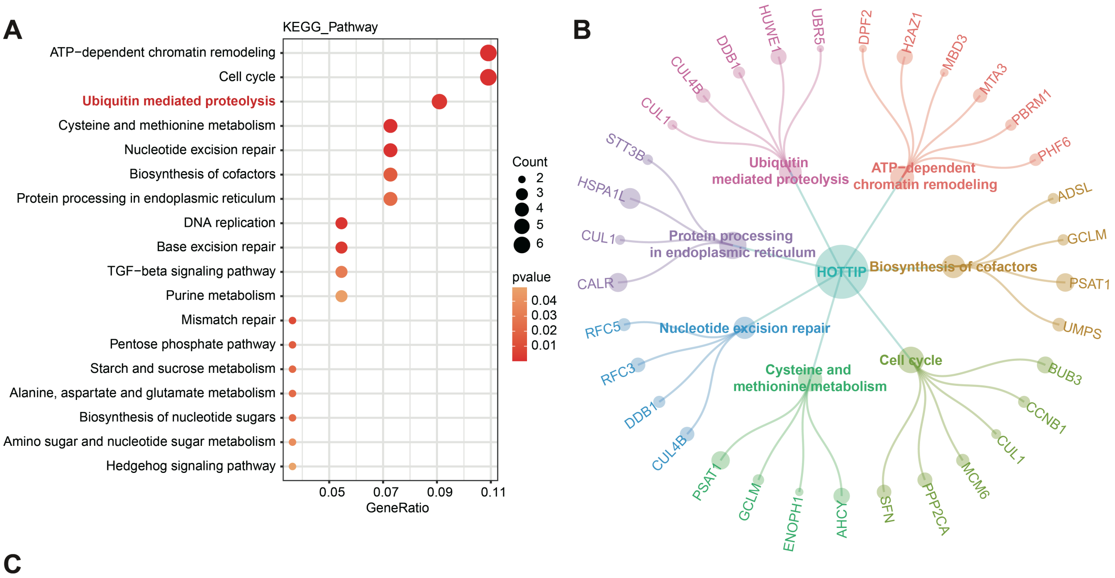

C

Protein Name	Score	Coverage	#Unique Peptides	#Peptides	#PSMs	#AAs	MW [kDa]	calc.pI
HUWE1	66.28	3	7	7	25	4373	481.6	5.22
CUL4B	49.42	10	3	7	25	913	103.9	7.37
DDB1	29.51	10	8	8	12	1140	126.9	5.26
CUL1	18.07	9	4	4	8	776	89.6	8
UBR5	13.08	4	5	5	6	2799	309.2	5.85

D E F

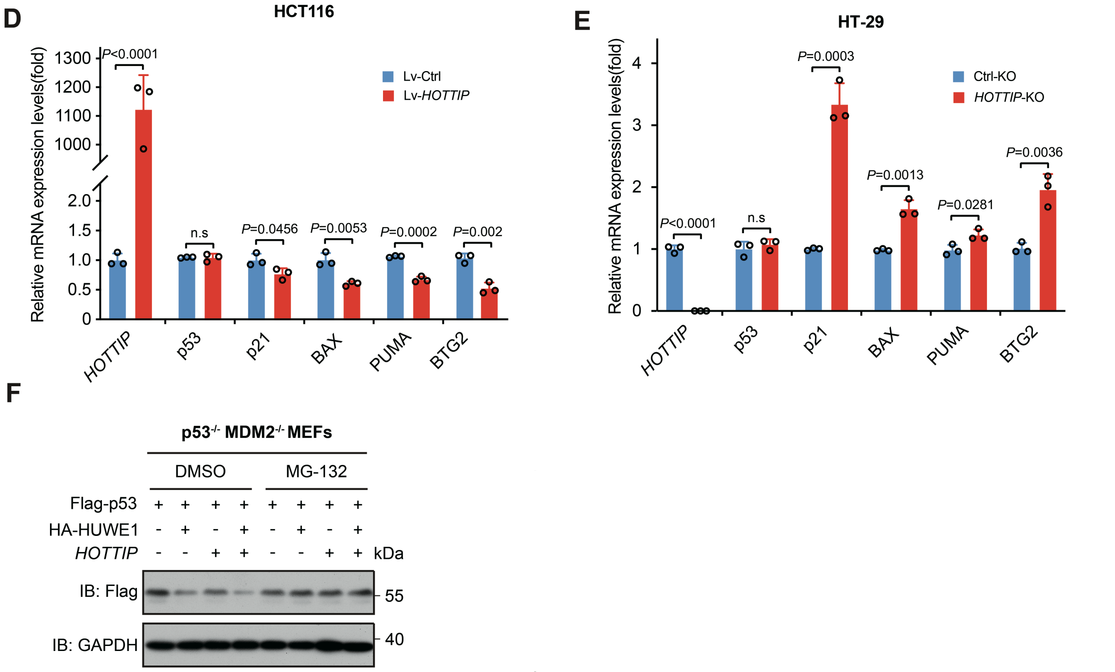

Supplementary Fig. 5 *HOTTIP* represses p53 activity by promoting HUWE1-mediated proteasomal degradation of p53. A Kyoto Encyclopedia

of Genes and Genomes (KEGG) enrichment analysis of differentially expressed proteins. **B** Heatmap showing the differentially expressed genes that exhibited enrichment in various downregulated KEGG pathways. **C** List of the proteins in the ubiquitin-mediated proteolysis signaling pathway with the top 5 scores determined by liquid chromatography-tandem mass spectrometry analysis of the proteins precipitated with HOTTIP. **D** Overexpression of *HOTTIP* decreases p53 target gene expression in HCT116 cells (n = 3 biologically independent samples). **E** Knockout of *HOTTIP* increases p53 target gene expression in HT-29 cells (n = 3 biologically independent samples). **F** *HOTTIP* suppresses HUWE1-mediated stabilization of p53 independent of MDM2. p53$^{-/-}$ MDM2$^{-/-}$ MEFs were transfected with Flag-p53, HA-HUWE1, or *HOTTIP* for 48 h and treated with MG-132 (20 μM, 6 h) before harvesting. The P values were calculated by a two-tailed unpaired t tests. The data are presented as the means ± SDs.

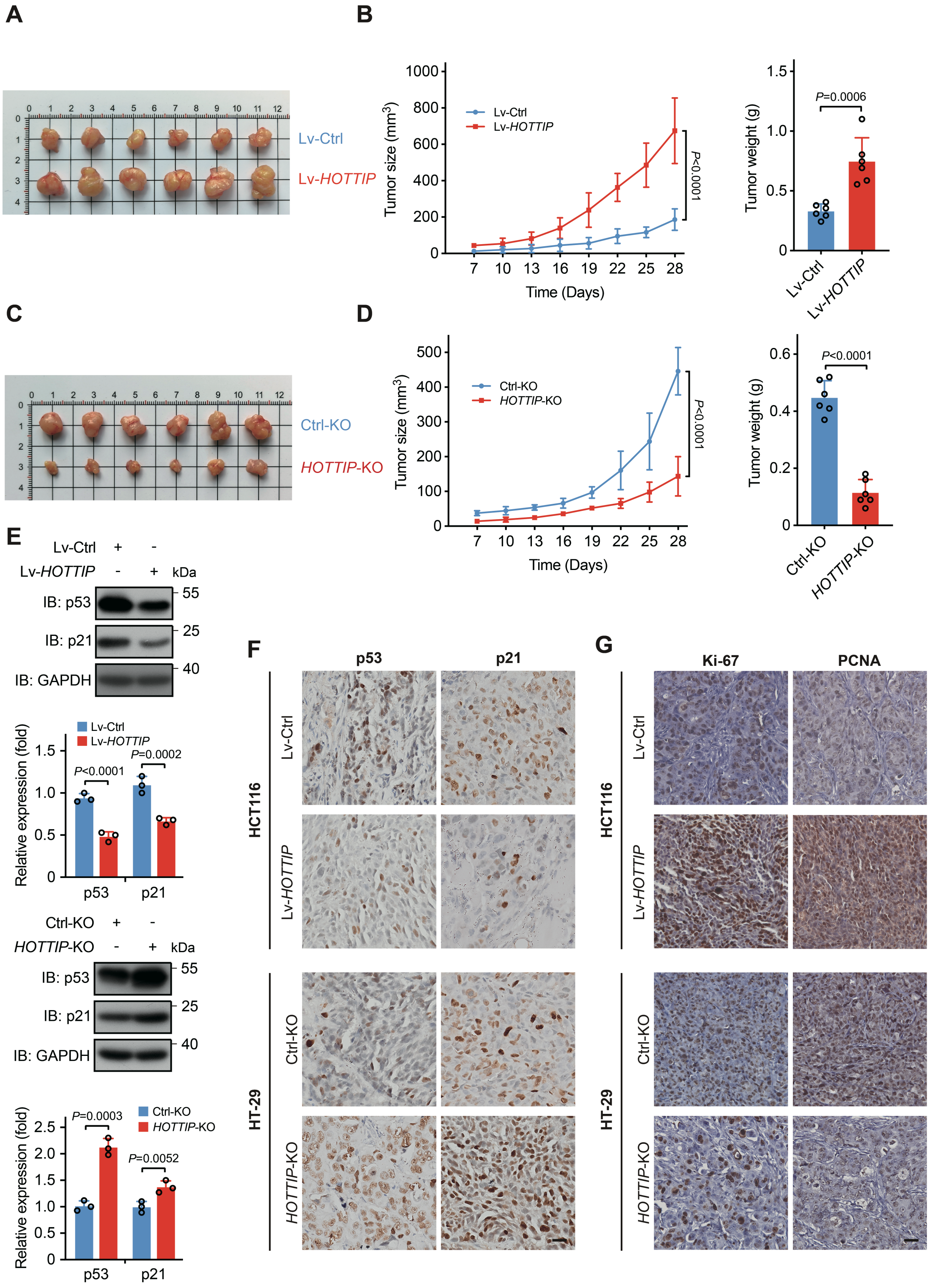

Supplementary Fig. 6 ***HOTTIP*** **promotes colorectal cancer cell proliferation and tumor growth in vivo. A** Representative images of

xenograft tumors formed by HCT116-Lv-Ctrl or HCT116-Lv-*HOTTIP* cells in nude mice. **B** Growth curves of xenograft tumors formed by HCT116 Lv-Ctrl or HCT116 Lv-*HOTTIP* cells in nude mice (left) and the weights of these tumors (right) (n = 6 biologically independent animals). **C** Representative images of xenograft tumors formed by HT-29 Ctrl-KO or HT-29 *HOTTIP*-KO cells in nude mice. **D** Growth curves of xenograft tumors formed by HT-29 Ctrl-KO or HT-29 *HOTTIP*-KO cells in nude mice (left) and the weights of these tumors (right) (n = 6 biologically independent animals). **E** p53 and p21 protein expression in tumors formed by HCT116 Lv-Ctrl, HCT116 Lv-*HOTTIP*, HT-29 Ctrl-KO or HT-29 *HOTTIP*-KO cells in nude mice. **F** Representative micrographs of immunochemical staining for p53 and p21 in tumors formed by HCT116 Lv-Ctrl, HCT116 Lv-*HOTTIP*, HT-29 Ctrl-KO or HT-29 *HOTTIP*-KO cells in nude mice; scale bars, 50 μm. **G** Representative micrographs of immunochemical staining for Ki-67 and PCNA in tumors formed by HCT116 Lv-Ctrl, HCT116 Lv-*HOTTIP*, HT-29 Ctrl-KO or HT-29 *HOTTIP*-KO cells in nude mice; scale bars, 50 μm. The P values were calculated by ANOVA and two-tailed unpaired t tests. The data are presented as the means ± SDs.

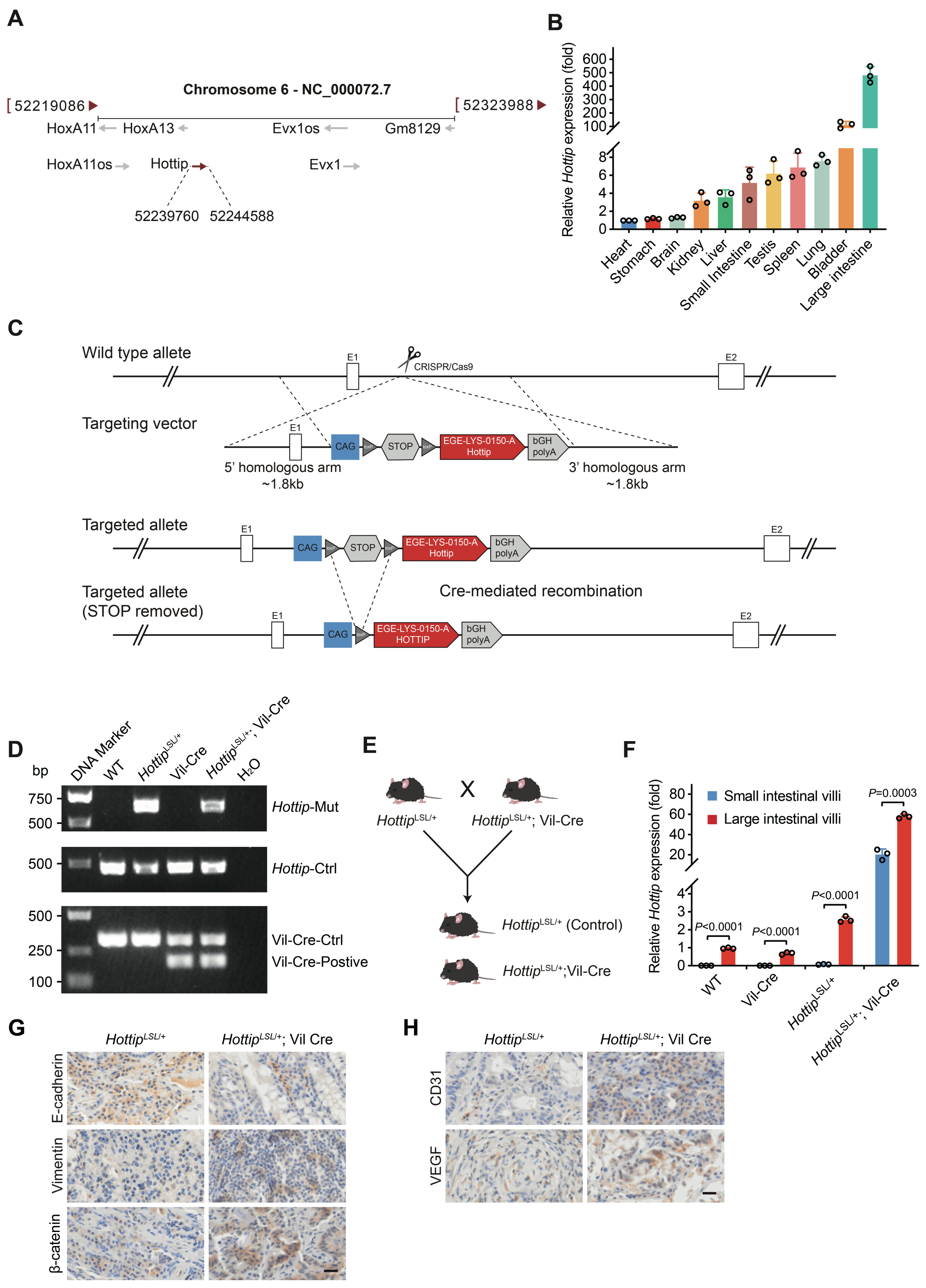

Supplementary Fig. 7 Generation and phenotype of *Hottip*$^{LSL/+}$ mice. A Depiction of the *Hottip* locus and its nearby coding genes. **B** The relative

expression of *Hottip* in different mouse tissues was measured via qPCR. **C** Generation of *Hottip*$^{LSL/+}$ mice. **D** PCR analysis of samples from 4-week-old mice revealed the presence of the WT, *Hottip*$^{LSL/+}$, and *Hottip*$^{LSL/LSL}$ alleles, as expected. **E** *Hottip*$^{LSL/+}$ mice were crossed with *Hottip*$^{LSL/+}$; Vil-Cre mice to generate *Hottip*$^{LSL/+}$ and *Hottip*$^{LSL/+}$; Vil-Cre progeny. **F** Relative expression of *Hottip* in the small intestinal villi and large intestinal villi of WT, Vil-Cre, *Hottip*/+, *Hottip*$^{LSL/+}$ and *Hottip*$^{LSL/+}$; Vil-Cre mice. **G** Representative micrographs of immunochemical staining for E-cadherin, Vimentin and β-catenin in tumors from control (*Hottip*$^{LSL/+}$) and *Hottip* KI (*Hottip*$^{LSL/+}$; Vil-Cre) mice after AOM/DSS treatment; scale bars, 50 μm. **H** Representative micrographs of immunochemical staining for VEGF and CD31 in tumors from control (*Hottip*$^{LSL/+}$) and *Hottip* KI (*Hottip*$^{LSL/+}$; Vil-Cre) mice after AOM/DSS treatment; scale bars, 50 μm. The *P* values were calculated by a two-tailed unpaired *t* tests. The data are presented as the means ± SDs.

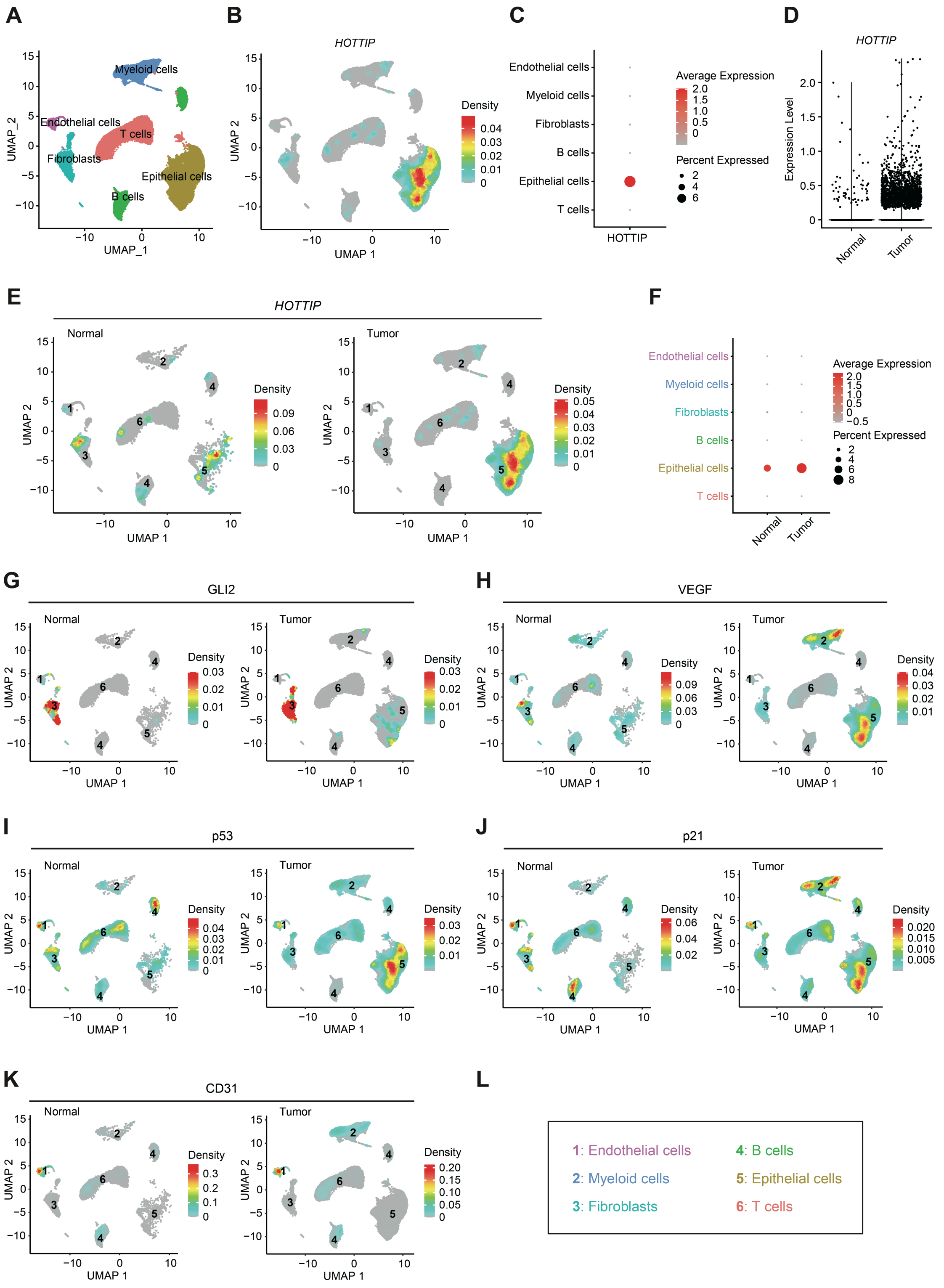

Supplementary Fig. 8 *HOTTIP* expression is high in colorectal cancer tumors. A UMAP plot of high-quality cells from all samples, colored by major cell types. **B** Density plot showing the cellular subpopulation localization of

HOTTIP across all samples. **C** Dot plot showing *HOTTIP* expression levels in major cell types across all samples. **D** Scatter plot showing the difference in *HOTTIP* expression levels and number of expressed cells between normal and tumor samples. **E**, **F** Density plot showing the cellular subpopulation localization of *HOTTIP* in normal and tumor samples, respectively. **G** Density plot showing the cellular subpopulation localization of GLI2 in normal and tumor samples. **H** Density plot showing the cellular subpopulation localization of VEGF in normal and tumor samples. **I** Density plot showing the cellular subpopulation localization of p53 in normal and tumor samples. **J** Density plot showing the cellular subpopulation localization of p21 in normal and tumor samples. **K** Density plot showing the cellular subpopulation localization of CD31 in normal and tumor samples. **L** Classification of six major cell types: (1) Endothelial cells, (2) Myeloid cells, (3) Fibroblasts, (4) B cells, (5) Epithelial cells, and (6) T cells.

Supplementary Tables

Supplementary Table 1 Reagents used in this study.

Assays	Reagents	Catalog No.	Vendors
Solvent for drug delivery	DMSO	D2650	Sigma
Hedgehog pathway inhibitor	GANT61	G9048	Sigma
Hedgehog pathway inhibitor	Tomatidine	T2909	Sigma
Hedgehog pathway inhibitor	Cyclopamine	S1146	Selleck Chemicals
Cell lysis	Cell Lysis Buffer	9803	Cell Signaling Technology
Protein analysis	Protein marker	LC5699	Thermo Fisher Scientific
Protein analysis	Protein marker	26616	Thermo Fisher Scientific
Transfection	Lipofectamine 2000 transfection	11668019	Thermo Fisher Scientific
Transfection	Polybrene	TR-1003	Sigma
Selection	Puromycin	P9620	Sigma
Carcinogen	Azoxymethane	A5486	Sigma
Colitis induction	Dextran sulfate sodium	9011-18-1	Affymetrix eBioscience
RNA isolation	TRIzol™	15596018CN	Thermo Fisher Scientific
Protein stabilization	Protease inhibitor cocktail	P8340	Sigma
RNA stabilization	Protector RNase Inhibitor	3335399001	Sigma
Proteasome inhibitor	MG-132	474791	Sigma
Autophagy inhibitor	Chloroquine	C6628	Sigma
Protein synthesis inhibitor	Cycloheximide	C7698	Sigma
Cell proliferation assay	Cell-Light EdU Apollo567 In Vitro Kit	C10310-1	RiboBio
Cell viability assay	Cell Counting Kit-8	GK10001	GLPBIO
RNA labeling	DIG RNA Labeling Kit	SP6/T7	Roche
Reverse transcription	PrimeScript® RT reagent Kit with gDNA Eraser	DRR047A	Takara

qPCR	SYBR® Premix Ex Taq II	DRR820A	Takara
Reporter assay	Dual-luciferase Reporter Assay Kit	E1910	Promega
RNA isolation	PARIS kit	AM1921	Thermo Fisher Scientific
DNA purification	QIAquick PCR Purification Kit	28106	QIAGEN
Organoid culture	G27	S223152	bioGenous
	Y-27632	S6390	Selleck Chemicals
	Organoid Culture ECM	M315066	bioGenous
	Tumor Tissue Digestion Solution	K601003	bioGenous
	Advanced DMEM/F-12	11320033	Thermo Fisher Scientific
	GlutaMAX	35050061	Thermo Fisher Scientific
	HEPES	15630130	Thermo Fisher Scientific
	penicillin-streptomycin	15140122	Thermo Fisher Scientific
	Primocin	ant-pm-1	Invitrogen
	N-acetylcysteine	A0737	Sigma
	R-Spondin1	861-RS1-1000	bioGenous
	Noggin	807-NOG-1000	bioGenous
	EGF	568-EGF-1000	bioGenous
	PGE2	S3003	Selleck Chemicals
	SB202190	S1077	Selleck Chemicals
	nicotinamide	S1899	Selleck Chemicals
	A-83-01	S7692	Selleck Chemicals
	Gastrin I	3006	R&D system

Supplementary Table 2 shRNAs used for knockdown of specific genes.

ID	Target sequence (5' to 3')
sh-*HOTTIP* (#1)	GCAGTTCTGGGTAAATATTGA
sh-*HOTTIP* (#2)	GGTGAAATACAAGCATCATAC
sh-GLI2 (#1)	CCGCTTCAGATGACAGATGTT
sh-GLI2 (#2)	GTTCCTGAACATGATGACCTA
sh-p53 (#1)	GTCCAGATGAAGCTCCCAGAA
sh-p53 (#2)	GAGGGATGTTTGGGAGATGTA

Supplementary Table 3 Antibodies used in this study.

Assays	Antibodies	Catalog No.	Vendors
ChIP + Immunoprecipitation	GLI2	ab26056	Abcam
	goat anti-rabbit IgG	31460	Thermo Fisher Scientific
	goat anti-mouse IgG	31430	Thermo Fisher Scientific
	IgG control		Thermo Fisher Scientific
Immunohistochemistry	Ki-67	12202S	Cell Signaling Technology
	PCNA	sc-56	Santa Cruz
	E-cadherin	14472s	Cell Signaling Technology
	vimentin	ab8979	Abcam
	β-catenin	610154	BD Biosciences
	VEGF	9698s	Cell Signaling Technology
	CD31	ab9498	Abcam
Flow cytometry	7-AAD	KGA219	KeyGen Biotech
	Ki-67	550609	BD Biosciences
Western blotting	GAPDH	MAB374	Millipore
	Flag	F3165	Sigma
	HA	3724s	Cell Signaling Technology
	Myc	M4439	Sigma
	p53	sc-126	Santa Cruz

	P21	2947	Cell Signaling Technology
	BTG2	ab197362	Abcam
	GLI2	ab26056	Abcam
	FOXM1	sc-376471	Santa Cruz
	BCL2	2876	Cell Signaling Technology
	PARP-1	sc-7150	Santa Cruz

Supplementary Table 4 Primers used for qPCR analyses.

**Gene**		**Sequence (5' to 3')**
HOTTIP	Forward	AAGGGTCTCAGCTCCACAGA
	Reverse	CTGCCGTCTTTTCTGAGTCC
GLI1	Forward	AGCGTGAGCCTGAATCTGTG
	Reverse	CAGCATGTACTGGGCTTTGAA
GLI2	Forward	CAGAATCGCACCCACTCCAACG
	Reverse	CGTGGACCGTTTTCACATGCTTCC
PTCH1	Forward	ACTCCCAAGCAAATGTACGAG
	Reverse	TTGAGTGGAGTTCTGTGCG
GAPDH	Forward	CACCAGGGCTGCTTTTAACTCTG
	Reverse	GATTTTGGAGGGATCTCGCTCCTG
U6	Forward	CTCGCTTCGGCAGCACATATACT
	Reverse	ATTTGCGTGTCATCCTTGCGCA
TUG1	Forward	TCCTTGTTTAGTGCATCTTTGCC
	Reverse	TGAGTGGTTATTCTGATAGCCTGC
NEAT1	Forward	GCATACGCAGCAGATCAGCAT
	Reverse	CCCACAATATAGGCATTTACAAGG
MALAT1	Forward	CCTAACCAGGCATAACACAGAAT
	Reverse	CGAATGGCTTTGTCTCCGAA
p53	Forward	CCCAAGCAATGGATGATTTGA
	Reverse	GGCATTCTGGGAGCTTCATCT

p21	Forward	CTGGACTGTTTTCTCTCGGCTC
	Reverse	TGTATATTCAGCATTGTGGGAGGA
BAX	Forward	CTGCAGAGGGATGATTGCCGCCG
	Reverse	TCCGGCACCTTGGTGCACAG
PUMA	Forward	ACAGTACGAGCGGCGGAGACAA
	Reverse	GGCGGGTGCAGGCACCTAATT
BTG2	Forward	CCAGGAGGCACTCACAGAGCA
	Reverse	ACCCACAGGGTCAGCTCGCT
Hottip	Forward	ATGCTCTGGGAAACGAAGCA
	Reverse	GCTTCCGTACCCAGCTACTC
Gapdh	Forward	TGTGTCCGTCGTGGATCTGA
	Reverse	TTGCTGTTGAAGTCGCAGGAG

Supplementary Table 5 sgRNA used for knockout of *HOTTIP.*

ID	Target sequence (5' to 3')
HOTTIP KO	CGCCGGCTTCGGAGTCACTTAGG

Supplementary Table 6 Primers used for ChIP analysis.

Gene	Target sequence (5' to 3')
HOTTIP -BS1-1-Forward	AGTGAAGAGCGCGCGGCGCGG
HOTTIP -BS1-1-Reverse	CACCGTGCAGAGCTGGAGTCG
HOTTIP -BS2-1-Forward	TGTCGTAGAGAAACATGACGG
HOTTIP -BS2-1-Reverse	CCGCGCGCTCTTCACTTCTTG
HOTTIP -BS3-1-Forward	CCGCCGCCCCTTCCATGTTCT
HOTTIP -BS3-1-Reverse	GTCTTCTCCATGCGGCTCGGG
HOTTIP -BS4-1-Forward	CCATCAGGTTGCGGCACTGGT
HOTTIP -BS4-1-Reverse	CGGCGGCAGGGGGCAACTTCT

Supplementary Table 7 Primers used for construction of luciferase reporter plasmids.

Promoters	Range	Sequence (5' to 3')
Frag-full-length-Forward	-4000~0	AGCCTCGAGGACCAGCTAAGGCCAATTCATGAGCTG
Frag-full-length-Reverse	-4000~0	GCCAAGCTTGCCCACTGGGATAAAGGAAGGGAAGG
Frag-I-Forward	-4000~-3000	AGCCTCGAGGACCAGCTAAGGCCAATTCATGAGCTG
Frag-I-Reverse	-4000~-3000	GCCAAGCTTGGTGAGATCTCCCGAGAGTTTCCATCCC
Frag-II-Forward	-3000~-1000	AGCCTCGAGGAGGGATGGAAACTCTCGGGAGATCTCAC
Frag-II-Reverse	-3000~-1000	GCCAAGCTTGGCCGGGCCTTACCACCACC
Frag-III-Forward	-1000~-0	AGCCTCGAGGAGGTGGTGGTAAGGCCCGGC
Frag-III-Reverse	-1000~-0	GCCAAGCTTGCCCACTGGGATAAAGGAAGGGAAGG

Supplementary Table 8 Identification of the binding proteins of *HOTTIP* was carried out using mass spectrometry analysis.

Protein	Unique peptides	MS score	Protein	Unique peptides	MS score	Protein	Unique peptides	MS score
RNH1	38	1886.23	STT3B	5	20.74	PIN4	3	24.77
CDC5L	20	165.61	PPP2CA	5	54.19	CCNB1	3	28.78
UBR4	14	67.06	CMSS1	5	31.32	CUL4B	3	49.42
DDX24	14	122.74	SIN3A	5	13.28	PCMT1	3	10.44
HELLS	13	105.05	ZNF598	5	14.62	ILVBL	3	29.19
ADD3	11	157.37	PNKP	5	24.65	APMAP	3	16.65
NCAPD3	11	111.55	SNX9	5	10.84	FAT1	3	37.02
PSAT1	11	99.46	EIF3F	5	50.43	PURB	3	18.11
VRK1	11	87.00	BOP1	4	44.75	OGFOD1	3	31.88
GTPBP4	10	29.06	GCLM	4	25.05	GTF3C2	3	16.92
SRPRB	10	107.77	OAS3	4	24.63	POTEF	2	181.08
MAP4	10	151.38	DHX30	4	22.09	HSPA1L	2	140.49
NAA16	9	86.15	CUL1	4	18.07	ALKBH5	2	51.08

ASS1	9	66.40	PUS1	4	11.15	MTA3	2	46.95
STRAP	9	40.29	H2AZ1	4	68.45	ERLIN1	2	33.23
COPB2	8	50.50	NUDT16	4	46.06	AGAP1	2	20.45
GPI	8	115.54	PBRM1	4	25.80	ARL1	2	18.68
DDB1	8	29.51	PHF6	4	32.40	SLC35E1	2	15.91
CNP	8	102.66	NEMF	4	17.09	MBD3	2	14.33
CALR	8	108.47	PDCD6	4	12.98	ENOPH1	2	13.93
NOP2	7	41.35	RTF2	4	74.43	GSTCD	2	17.79
IGF2BP2	7	83.66	SPAST	4	40.83	SRPK2	2	60.28
NCAPG2	7	30.37	EIF6	4	25.56	PATL1	2	33.22
AHCY	7	71.39	ZMPSTE24	4	27.00	PTRH2	2	40.96
HUWE1	7	66.28	XPNPEP1	4	18.39	NOB1	2	16.35
UMPS	6	42.13	TBL2	3	51.45	BAG5	2	10.92
MCM6	6	37.23	LPCAT1	3	44.61	SRSF3	2	45.32
GALNT2	6	53.29	USP5	3	36.55	ATE1	2	14.19
RFC3	6	51.74	STK10	3	29.98	SFN	2	50.80
BUB3	6	77.26	RNMT	3	25.41	NUFIP2	2	10.57
ARRB1	6	60.14	XPO5	3	25.12	CDK9	2	18.28
KPNA3	6	96.47	ASCC2	3	21.92	LSM14B	2	11.17
RFC5	6	35.94	CSTB	3	10.27	MYH14	2	119.52
LMCD1	5	32.12	PURA	3	43.08	PGM2	2	23.30
ARMT1	5	26.53	ADSL	3	23.16	REPIN1	2	17.23
UBR5	5	13.08	DPF2	3	13.35	OVCA2	2	12.66
RNF20	5	45.70	DDX54	3	16.20	GSDMA	2	21.30
AGR2	5	95.06	BLVRA	3	13.25			

Supplementary Table 9 Primers for the mouse model.

Gene		Sequence (5' to 3')
LSL-*Hottip*-Mutant	Forward	CTCGACTGTGCCTTCTAGTTGCCAG
	Reverse	TGAGCATGTCTTTAATCTACCTCGATG
LSL-*Hottip*-WT	Forward	AGTCGCTCTGAGTTGTTATCAG
	Reverse	TGAGCATGTCTTTAATCTACCTCGATG
Vil-Cre	Forward	TTCTCCTCTAGGCTCGTCCA
	Reverse	CATGTCCATCAGGTTCTTGC
Vil-Positive Con	Forward	CTAGGCCACAGAATTGAAAGATCT
	Reverse	GTAGGTGGAAATTCTAGCATCATCC

Supplementary Table 10 Association of *HOTTIP* expression with different clinical characteristics in colorectal cancer.

Characteristics		HOTTIP expression		Total	χ^2 value	*P* value
		Low expression (%)	High expression (%)			
**Age**	≤ 60	28 (54.9)	23 (45.1)	51	0.853	0.356
	> 60	32 (46.4)	37 (53.6)	69		
**Gender**	Male	32 (53.3)	28 (46.7)	60	0.533	0.465
	Female	28 (46.7)	32 (53.3)	60		
**CEA level, ng/ml**	≤ 5	40 (52.6)	36 (47.4)	76	0.574	0.449
	> 5	20 (45.5)	24 (54.5)	44		
**AFP level, ng/ml**	≤ 8.1	52 (48.6)	55 (51.4)	107	0.776	0.378
	> 8.1	8 (61.5)	5 (38.5)	13		
**CA19-9 level, U/ml**	≤ 37	47 (52.8)	42 (47.2)	89	1.087	0.297
	> 37	13 (41.9)	18 (58.1)	31		
**CA125 level, U/ml**	≤ 35	50 (51.5)	47 (48.5)	97	0.137	0.711
	> 35	3 (60.0)	2 (40.0)	5		
	Unkonwn	7 (38.9)	11 (61.1)	18		
**General type**	Ulcerative type	38 (51.4)	36 (48.6)	74	1.101	0.577
	Protruding type	21 (50.0)	21 (50.0)	42		
	Infiltrative type	1 (25.0)	3 (75.0)	4		
**Tumor location**	Left hemicolon	13 (48.1)	14 (51.9)	27	0.564	0.754
	Right hemicolon	19 (46.3)	22 (53.7)	41		
	Rectum	28 (53.8)	24 (46.2)	52		
**Primary tumor size, cm**	< 4	28 (63.6)	16 (36.4)	44	5.234	0.073
	4 ~ 6	22 (43.1)	29 (56.9)	51		
	> 6	10 (40.0)	15 (60.0)	25		
**Differentiation degree**	Well and moderate	53 (55.8)	42 (44.2)	95	6.114	0.013*
	Poor	7 (28.0)	18 (72.0)	25		
**TNM Stage**	I ~ II	38 (69.1)	17 (30.9)	55	14.803	<0.001***
	III ~ IV	22 (33.8)	43 (66.2)	65		
**T stage**	T1 ~ T2	16 (76.2)	5 (23.8)	21	6.984	0.008**
	T3 ~ T4	44 (44.4)	55 (55.6)	99		
**N stage**	N0	36 (66.7)	18 (33.3)	54	10.909	<0.001***
	N1 ~ N3	24 (36.4)	42 (63.6)	66		
**M stage**	M0	56 (51.9)	52 (48.1)	108	1.481	0.224
	M1	4 (33.3)	8 (66.7)	12		
**Vascular invasion**	Negative	45 (57.0)	34 (43.0)	79	4.483	0.034*
	Positive	15 (36.6)	26 (63.4)	41		
**Nerve invasion**	Negative	47 (55.3)	38 (44.7)	85	3.267	0.071
	Positive	13 (37.1)	22 (62.9)	35		

Note: The χ^2 test was used to analyze the correlation between gene expression and clinicopathological characteristics.

Supplementary Table 11 Table Association between *HOTTIP* expression and clinical features in colorectal cancer patients with different TNM stages.

Characteristics		TNM Stage I ~ II		Total	χ^2 value	*P* value	TNM Stage III ~ IV		Total	χ^2 value	*P* value
		Low HOTTIP expression (%)	High HOTTIP expression (%)				Low HOTTIP expression (%)	High HOTTIP expression (%)			
**Age**	≤ 60	21 (80.8)	5 (19.2)	26	3.149	0.076	7 (28.0)	18 (72.0)	25	0.620	0.431
	> 60	17 (58.6)	12 (41.4)	29			15 (37.5)	25 (62.5)	40		
**Gender**	Male	19 (76.0)	6 (24.0)	25	1.025	0.311	13 (37.1)	22 (62.9)	35	0.368	0.544
	Female	19 (63.3)	11 (36.7)	30			9 (30.0)	21 (70.0)	30		
**CEA level, ng/ml**	≤ 5	27 (69.2)	12 (30.8)	39	0.001	0.972	13 (35.1)	24 (64.9)	37	0.064	0.801
	> 5	11 (68.8)	5 (31.2)	16			9 (32.1)	19 (67.9)	28		
**AFP level, ng/ml**	≤ 8.1	33 (71.7)	13 (28.3)	46	0.321	0.571	19 (31.1)	42 (68.9)	61	1.563	0.211
	> 8.1	5 (55.6)	4 (44.4)	9			3 (75.0)	1 (25.0)	4		
**CA19-9 level, U/ml**	≤ 37	32 (68.1)	15 (31.9)	47	0.153	0.696	15 (35.7)	27 (64.3)	42	0.185	0.667
	> 37	6 (75.0)	2 (25.0)	8			7 (30.4)	16 (69.6)	23		
**CA125 level, U/ml**	≤ 35	31 (72.1)	12 (27.9)	43	1.274	0.259	19 (35.2)	35 (64.8)	54	0.004	0.948
	> 35	2 (100.0)	0 (0.0)	2			1 (33.3)	2 (66.7)	3		
	Unkonwn	5 (50.0)	5 (50.0)	10			2 (25.0)	6 (75.0)	8		
**General type**	Ulcerative type	18 (66.7)	9 (33.3)	27	0.834	0.659	20 (42.6)	27 (57.4)	47	5.945	0.051
	Protruding type	19 (70.4)	8 (29.6)	27			2 (13.3)	13 (86.7)	15		
	Infiltrative type	1 (100.0)	0 (0.0)	1			0 (0.0)	3 (100.0)	3		
**Tumor location**	Left hemicolon	9 (75.0)	3 (25.0)	12	0.260	0.878	4 (26.7)	11 (73.3)	15	1.038	0.595
	Right hemicolon	12 (66.7)	6 (33.3)	18			7 (30.4)	16 (69.6)	23		

	Rectum	17 (68.0)	8 (32.0)	25			11 (40.7)	16 (59.3)	27		
**Primary tumor size, cm**	< 4	18 (75.0)	6 (25.0)	24	5.171	0.072	10 (50.0)	10 (50.0)	20	4.532	0.104
	4 ~ 6	15 (78.9)	4 (21.1)	19			7 (21.9)	25 (78.1)	32		
	> 6	5 (41.7)	7 (58.3)	12			5 (38.5)	8 (61.5)	13		
**Differentiation degree**	Well and moderate	38 (76.0)	12 (24.0)	50	12.294	< 0.001***	15 (33.3)	30 (66.7)	45	0.017	0.896
	Poor	0 (0.0)	5 (100.0)	5			7 (35.0)	13 (65.0)	20		
**T stage**	T1 ~ T2	14 (73.7)	5 (26.3)	19	0.287	0.592	2 (100.0)	0 (0.0)	2	4.459	0.035*
	T3 ~ T4	24 (66.7)	12 (33.3)	36			20 (31.7)	43 (68.3)	63		
**N stage**	N0	35 (68.6)	16 (31.4)	51	0.073	0.787	1 (33.3)	2 (66.7)	3	0.001	0.985
	N1 ~ N3	3 (75.0)	1 (25.0)	4			21 (33.9)	41 (66.1)	62		
**M stage**	M0	-	-	-	-	-	18 (34.0)	35 (66.0)	53	0.002	0.967
	M1	38 (69.1)	17 (30.9)	55			4 (33.3)	8 (66.7)	12		
**Vascular invasion**	Negative	38 (73.1)	14 (26.9)	52	7.442	0.006**	7 (25.9)	20 (74.1)	27	1.294	0.255
	Positive	0 (0.0)	3 (100.0)	3			15 (39.5)	23 (60.5)	38		
**Nerve invasion**	Negative	32 (69.6)	14 (30.4)	46	0.029	0.864	15 (38.5)	24 (61.5)	39	0.928	0.335
	Positive	6 (66.7)	3 (33.3)	9			7 (26.9)	19 (73.1)	26		

Note: The χ^2 test was used to analyze the correlation between gene expression and clinicopathological characteristics.

Supplementary Table 12 Association between *HOTTIP* expression and clinical features in colorectal cancer patients with different differentiation degree.

Characteristics		Differentiation degree - Well and moderate		Total	χ^2 value	*P* value	Differentiation degree - Poor		Total	χ^2 value	*P* value
		Low HOTTIP expression (%)	High HOTTIP expression (%)				Low HOTTIP expression (%)	High HOTTIP expression (%)			
**Age**	≤ 60	27 (61.4)	17 (38.6)	44	1.032	0.310	1 (14.3)	6 (85.7)	7	0.208	0.648
	> 60	26 (51.0)	25 (49.0)	51			6 (33.3)	12 (66.7)	18		
**Gender**	Male	28 (57.1)	21 (42.9)	49	0.075	0.784	4 (36.4)	7 (63.6)	11	0.679	0.410
	Female	25 (54.3)	21 (45.7)	46			3 (21.4)	11 (78.6)	14		
**CEA level, ng/ml**	≤ 5	37 (59.7)	25 (40.3)	62	1.094	0.296	3 (21.4)	11 (78.6)	14	0.679	0.410
	> 5	16 (48.5)	17 (51.5)	33			4 (36.4)	7 (63.6)	11		
**AFP level, ng/ml**	≤ 8.1	46 (54.1)	39 (45.9)	85	0.384	0.535	6 (27.3)	16 (72.7)	22	0.047	0.829
	> 8.1	7 (70.0)	3 (30.0)	10			1 (33.3)	2 (66.7)	3		
**CA19-9 level, U/ml**	≤ 37	43 (58.1)	31 (41.9)	74	0.730	0.393	4 (26.7)	11 (73.3)	15	0.033	0.856
	> 37	10 (47.6)	11 (52.4)	21			3 (30.0)	7 (10.0)	10		
**CA125 level, U/ml**	≤ 35	44 (55.0)	36 (45.0)	80	3.498	0.061	6 (35.3)	11 (64.7)	17	1.624	0.202
	> 35	3 (100.0)	0 (0.0)	3			0 (0.0)	2 (100.0)	2		
	Unkonwn	6 (50.0)	6 (50.0)	12			1 (16.7)	5 (83.3)	6		
**General type**	Ulcerative type	31 (55.4)	25 (44.6)	56	0.045	0.978	7 (38.9)	11 (61.1)	18	5.591	0.061
	Protruding type	21 (56.8)	16 (43.2)	37			0 (0.0)	5 (100.0)	5		
	Infiltrative type	1 (50.0)	1 (50.0)	2			0 (0.0)	2 (100.0)	2		
**Tumor location**	Left hemicolon	12 (54.5)	10 (45.5)	22	0.231	0.891	1 (20.0)	4 (80.0)	5	0.688	0.709
	Right	17 (53.1)	15 (46.9)	32			2 (22.2)	7 (77.8)	9		

	hemicolon										
	Rectum	24 (58.5)	17 (41.5)	41			4 (36.4)	7 (63.6)	11		
**Primary tumor size, cm**	< 4	26 (68.4)	12 (31.6)	38	4.975	0.083	2 (33.3)	4 (66.7)	6	3.143	0.208
	4 ~ 6	21 (51.2)	20 (48.8)	41			1 (10.0)	9 (90.0)	10		
	> 6	6 (37.5)	10 (62.5)	16			4 (44.4)	5 (55.6)	9		
**TNM Stage**	I ~ II	38 (76.0)	12 (24.0)	50	17.481	< 0.001***	0 (0.0)	5 (100.0)	5	3.750	0.053
	III ~ IV	15 (33.3)	30 (66.7)	45			7 (35.0)	13 (65.0)	20		
**T stage**	T1 ~ T2	16 (84.2)	3 (15.8)	19	7.778	0.005**	0 (0.0)	2 (100.0)	2	1.381	0.240
	T3 ~ T4	37 (48.7)	39 (51.3)	76			7 (30.4)	16 (69.6)	23		
**N stage**	N0	36 (75.0)	12 (25.0)	48	14.517	< 0.001***	0 (0.0)	6 (100.0)	6	4.639	0.031*
	N1 ~ N3	17 (36.2)	30 (63.8)	47			7 (36.8)	12 (63.2)	19		
**M stage**	M0	50 (58.8)	35 (41.2)	85	1.958	0.162	6 (26.1)	17 (73.9)	23	0.473	0.492
	M1	3 (30.0)	7 (70.0)	10			1 (50.0)	1 (50.0)	2		
**Vascular invasion**	Negative	43 (63.2)	25 (36.8)	68	5.378	0.020*	2 (18.2)	9 (81.8)	11	0.967	0.325
	Positive	10 (37.0)	17 (63.0)	27			5 (35.7)	9 (64.3)	14		
**Nerve invasion**	Negative	43 (61.4)	27 (38.6)	70	3.429	0.064	4 (26.7)	11 (73.3)	15	0.033	0.856
	Positive	10 (40.0)	15 (60.0)	25			3 (30.0)	7 (70.0)	10		

Note: The χ^2 test was used to analyze the correlation between gene expression and clinicopathological characteristics.

Supplementary Table 13 Association between *HOTTIP* expression and clinical features in colorectal cancer patients with and without vascular invasion.

Characteristics		Vascular invasion - Negative		Total	χ^2 value	*P* value	Vascular invasion - Positive		Total	χ^2 value	*P* value
		Low HOTTIP expression (%)	High HOTTIP expression (%)				Low HOTTIP expression (%)	High HOTTIP expression (%)			
**Age**	≤ 60	24 (63.2)	14 (36.8)	38	1.147	0.284	4 (30.8)	9 (69.2)	13	0.032	0.858
	> 60	21 (51.2)	20 (48.8)	41			11 (39.3)	17 (60.7)	28		
**Gender**	Male	24 (60.0)	16 (40.0)	40	0.305	0.581	8 (40.0)	12 (60.0)	20	0.196	0.658
	Female	21 (53.8)	18 (46.2)	39			7 (33.3)	14 (66.7)	21		
**CEA level, ng/ml**	≤ 5	31 (58.5)	22 (41.5)	53	0.153	0.695	9 (39.1)	14 (60.9)	23	0.146	0.702
	> 5	14 (53.8)	12 (46.2)	26			6 (33.3)	12 (66.7)	18		
**AFP level, ng/ml**	≤ 8.1	38 (55.1)	31 (44.9)	69	0.302	0.583	14 (36.8)	24 (63.2)	38	0.015	0.903
	> 8.1	7 (70.0)	3 (30.0)	10			1 (33.3)	2 (66.7)	3		
**CA19-9 level, U/ml**	≤ 37	36 (56.3)	28 (43.8)	64	0.070	0.792	11 (44.0)	14 (56.0)	25	1.518	0.218
	> 37	9 (60.0)	6 (40.0)	15			4 (25.0)	12 (75.0)	16		
**CA125 level, U/ml**	≤ 35	36 (57.1)	27 (42.9)	63	0.522	0.470	14 (41.2)	20 (58.8)	34	1.041	0.308
	> 35	3 (75.0)	1 (25.0)	4			0 (0.0)	1 (100.0)	1		
	Unkonwn	6 (50.0)	6 (50.0)	12			1 (16.7)	5 (83.3)	6		
**General type**	Ulcerative type	24 (52.2)	22 (47.8)	46	2.266	0.322	14 (50.0)	14 (50.0)	28	6.877	0.032*
	Protruding type	20 (66.7)	10 (33.3)	30			1 (8.3)	11 (91.7)	12		
	Infiltrative type	1 (33.3)	2 (66.7)	3			0 (0.0)	1 (100.0)	1		
**Tumor location**	Left hemicolon	9 (50.0)	9 (50.0)	18	0.555	0.758	4 (44.4)	5 (55.6)	9	0.321	0.852
	Right hemicolon	13 (56.5)	10 (43.5)	23			6 (33.3)	12 (66.7)	18		

	Rectum	23 (60.5)	15 (39.5)	38			5 (35.7)	9 (64.3)	14		
**Primary tumor size, cm**	< 4	20 (64.5)	11 (35.5)	31	1.867	0.393	8 (61.5)	5 (38.5)	13		
	4 ~ 6	18 (56.3)	14 (43.8)	32			4 (21.1)	15 (78.9)	19		
	> 6	7 (43.8)	9 (56.3)	16			3 (33.3)	6 (66.7)	9		
**Differentiation degree**	Well and moderate	43 (63.2)	25 (36.8)	68	6.110	0.013*	10 (37.0)	17 (63.0)	27	0.007	0.934
	Poor	2 (18.2)	9 (81.8)	11			5 (35.7)	9 (64.3)	14		
**TNM Stage**	I ~ II	38 (73.1)	14 (26.9)	52	16.117	< 0.001***	0 (0.0)	3 (100.0)	3	2.868	0.090
	III ~ IV	7 (25.9)	20 (74.1)	27			15 (39.5)	23 (60.5)	38		
**T stage**	T1 ~ T2	14 (82.4)	3 (17.6)	17	5.696	0.017*	2 (50.0)	2 (50.0)	4	0.333	0.564
	T3 ~ T4	31 (50.0)	31 (50.0)	62			13 (35.1)	24 (64.9)	37		
**N stage**	N0	36 (70.6)	15 (29.4)	51	10.898	0.001**	0 (0.0)	3 (100.0)	3	2.868	0.090
	N1 ~ N3	9 (32.1)	19 (67.9)	28			15 (39.5)	23 (60.5)	38		
**M stage**	M0	43 (60.6)	28 (39.4)	71	3.749	0.053	13 (35.1)	24 (64.9)	37	0.333	0.564
	M1	2 (25.0)	6 (75.0)	8			2 (50.0)	2 (50.0)	4		
**Nerve invasion**	Negative	38 (59.4)	26 (40.6)	64	0.801	0.371	9 (42.9)	12 (57.1)	21	0.730	0.393
	Positive	7 (46.7)	8 (53.3)	15			6 (30.0)	14 (70.0)	20		

Note: The χ^2 test was used to analyze the correlation between gene expression and clinicopathological characteristics.

Supplementary Table 14 Association of GLI2 expression with different clinical characteristics in colorectal cancer.

Characteristics		GLI2 expression		Total	χ^2 value	*P* value
		Low expression (%)	High expression (%)			
**Age**	≤ 60	29 (56.9)	22 (43.1)	51	1.671	0.196
	> 60	31 (44.9)	38 (55.1)	69		
**Gender**	Male	32 (53.3)	28 (46.7)	60	0.533	0.465
	Female	28 (46.7)	32 (53.3)	60		
**CEA level, ng/ml**	≤ 5	41 (53.9)	35 (46.1)	76	1.292	0.256
	> 5	19 (43.2)	25 (56.8)	44		
**AFP level, ng/ml**	≤ 8.1	53 (49.5)	54 (50.5)	107	0.086	0.769
	> 8.1	7 (53.8)	6 (46.2)	13		
**CA19-9 level, U/ml**	≤ 37	49 (55.1)	40 (44.9)	89	3.523	0.061
	> 37	11 (35.5)	20 (64.5)	31		
**CA125 level, U/ml**	≤ 35	50 (51.5)	47 (48.5)	97	0.255	0.614
	> 35	2 (40.0)	3 (60.0)	5		
	Unkonwn	8 (44.4)	10 (55.6)	18		
**General type**	Ulcerative type	33 (44.6)	41 (55.4)	74	2.400	0.301
	Protruding type	25 (59.5)	17 (40.5)	42		
	Infiltrative type	2 (50.0)	2 (50.0)	4		
**Tumor location**	Left hemicolon	16 (59.3)	11 (40.7)	27	2.198	0.333
	Right hemicolon	17 (41.5)	24 (58.5)	41		
	Rectum	27 (51.9)	25 (48.1)	52		
**Primary tumor size, cm**	< 4	26 (59.1)	18 (40.9)	44	2.631	0.268
	4 ~ 6	24 (47.1)	27 (52.9)	51		
	> 6	10 (40.0)	15 (60.0)	25		
**Differentiation degree**	Well and moderate	57 (60.0)	38 (40.0)	95	18.240	< 0.001***
	Poor	3 (12.0)	22 (88.0)	25		
**TNM Stage**	I ~ II	37 (67.3)	18 (32.7)	55	12.117	< 0.001***
	III ~ IV	23 (35.4)	42 (64.6)	65		
**T stage**	T1 ~ T2	14 (66.7)	7 (33.3)	21	2.828	0.093
	T3 ~ T4	46 (46.5)	53 (53.5)	99		

**N stage**	N0	35 (64.8)	19 (35.2)	54	8.620	0.003**
	N1 ~ N3	25 (37.9)	41 (62.1)	66		
**M stage**	M0	55 (50.9)	53 (49.1)	108	0.370	0.543
	M1	5 (41.7)	7 (58.3)	12		
**Vascular invasion**	Negative	46 (58.2)	33 (41.8)	79	6.261	0.012*
	Positive	14 (34.1)	27 (65.9)	41		
**Nerve invasion**	Negative	46 (54.1)	39 (45.9)	85	1.976	0.160
	Positive	14 (40.0)	21 (60.0)	35		

Note: The χ^2 test was used to analyze the correlation between gene expression and clinicopathological characteristics.

Supplementary Table 15 Association between GLI2 expression and *HOTTIP* expression in colorectal cancer patients with different clinicopathologic characteristics.

Characteristics		GLI2 expression	HOTTIP expression: Low expression (%)	HOTTIP expression: High expression (%)	Total	χ^2 value	*P* value
**TNM Stage**	I ~ II	Low expression	31 (81.6)	7 (18.4)	38	11.429	<0.001***
		High expression	6 (35.3)	11 (64.7)	17		
	III ~ IV	Low expression	9 (40.9)	13 (59.1)	22	0.444	0.505
		High expression	14 (32.6)	29 (67.4)	43		
**Differentiation degree**	Well and moderate	Low expression	37 (69.8)	16 (30.2)	53	4.808	0.028*
		High expression	20 (47.6)	22 (52.4)	42		
	Poor	Low expression	3 (42.9)	4 (57.1)	7	8.786	0.003**
		High expression	0 (0.0)	18 (100.0)	18		
**Vascular invasion**	Negative	Low expression	33 (73.3)	12 (26.7)	45	9.809	0.002**
		High expression	13 (38.2)	21 (61.8)	34		
	Positive	Low expression	7 (46.7)	8 (53.3)	15	1.649	0.199
		High expression	7 (26.9)	19 (73.1)	26		
**Total**		Low expression	40 (66.7)	20 (33.3)	60	13.333	< 0.001***
		High expression	20 (33.3)	40 (66.7)	60		

Note: The χ^2 test was used to analyze the correlation between gene expression and clinicopathological characteristics.
