# Supplementary material for: LncRNA HOTTIP modulated by Hedgehog signaling drives colorectal cancer progression by promoting HUWE1-mediated ubiquitin‒proteasome degradation of p53
Source: Cell Death Dis. 2025 Jul 7;16(1):502. doi: 10.1038/s41419-025-07817-4 (PMC12234969; doi:10.1038/s41419-025-07817-4)

Fig. 1J

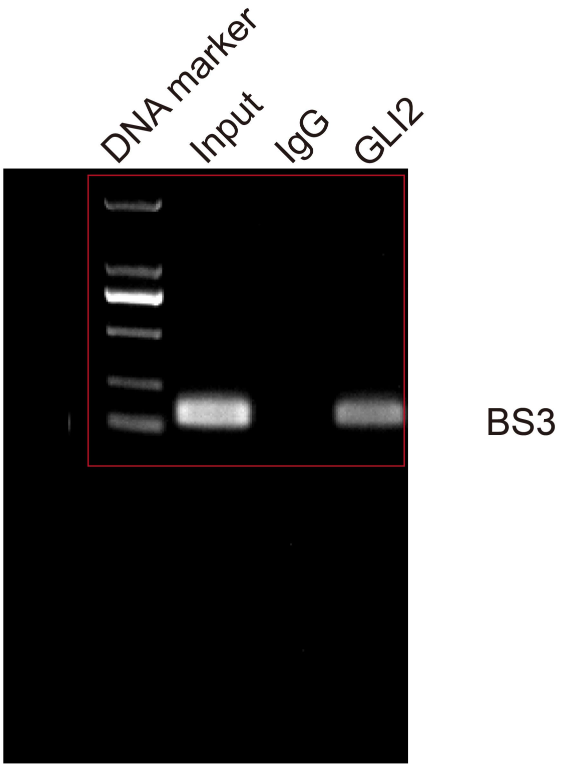

Fig. 3l

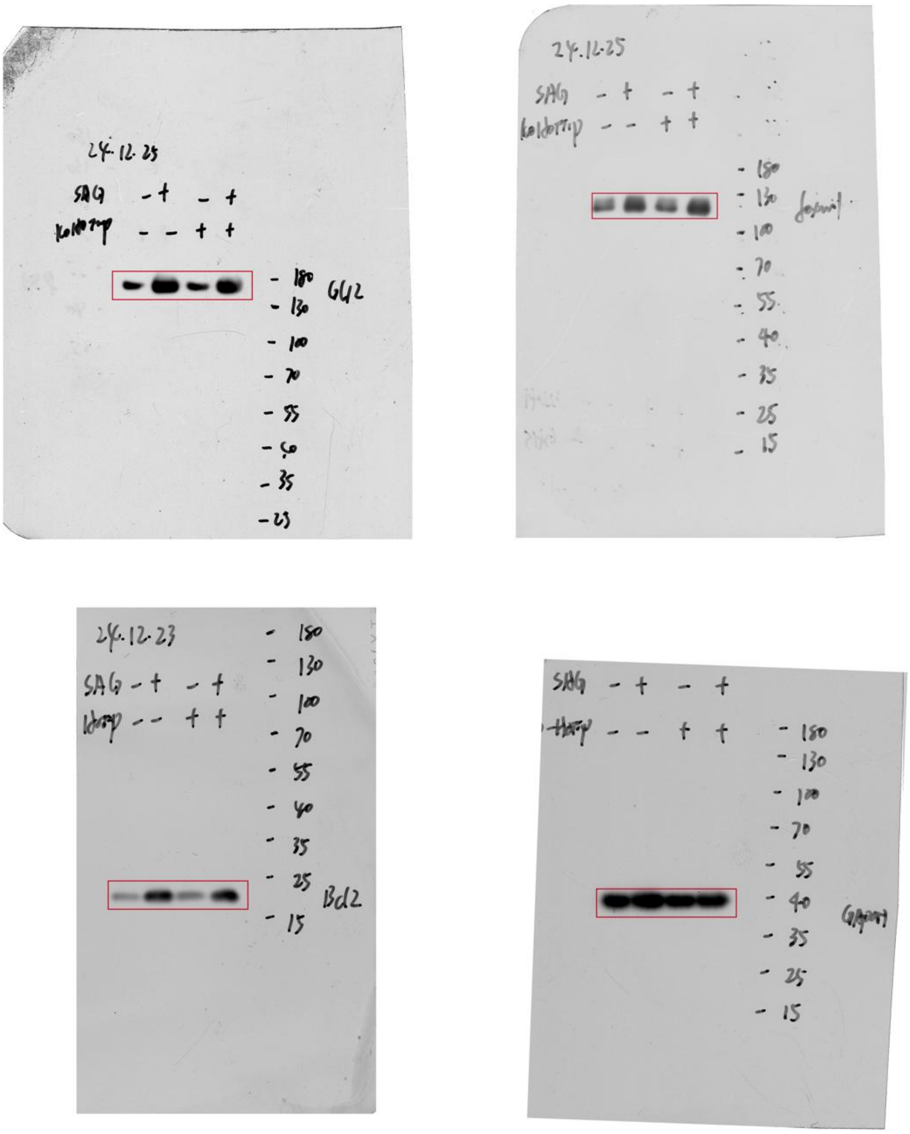

Fig. 4A

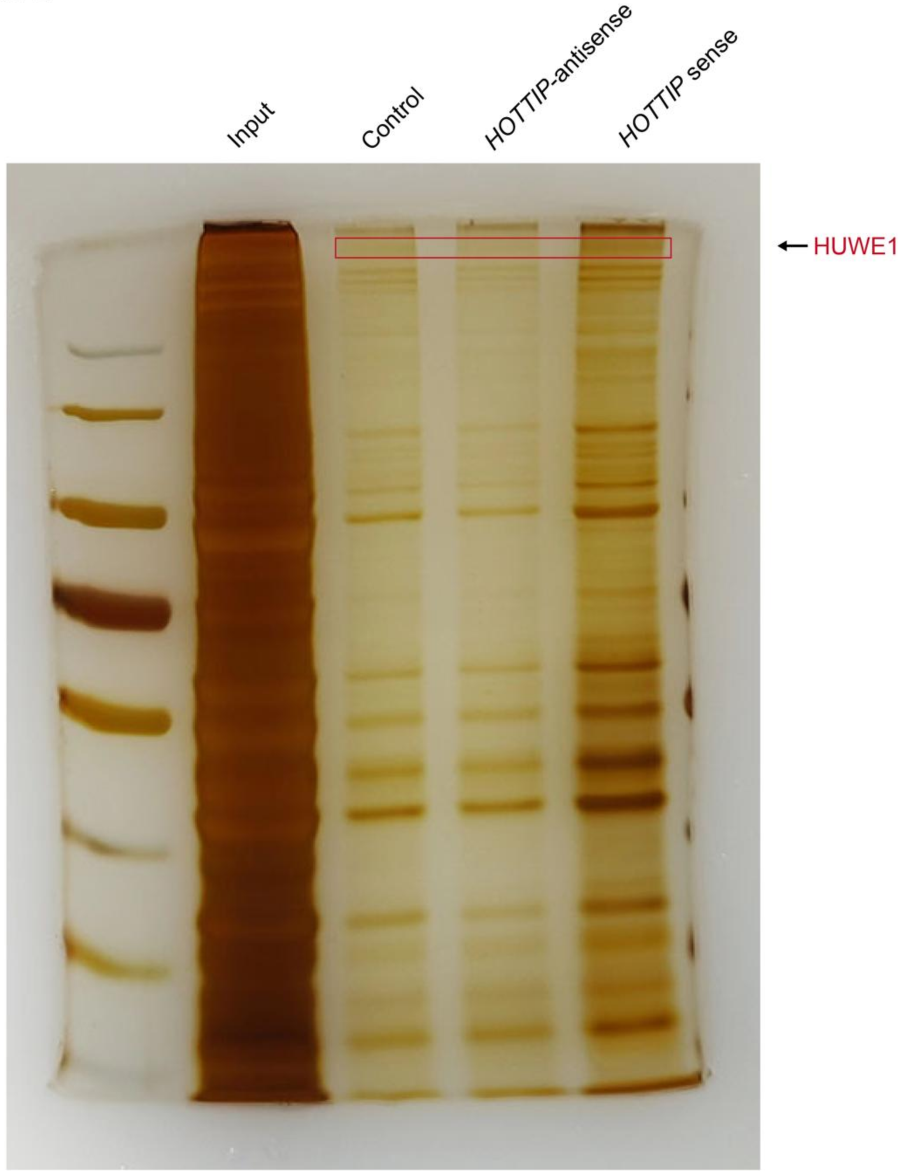

Fig. 4B

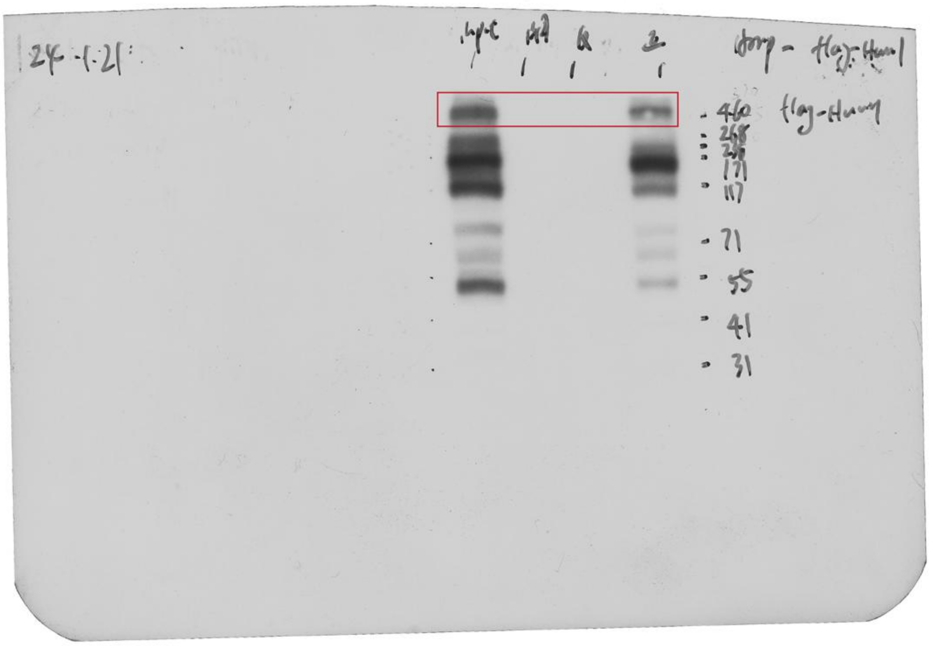

Fig. 4D

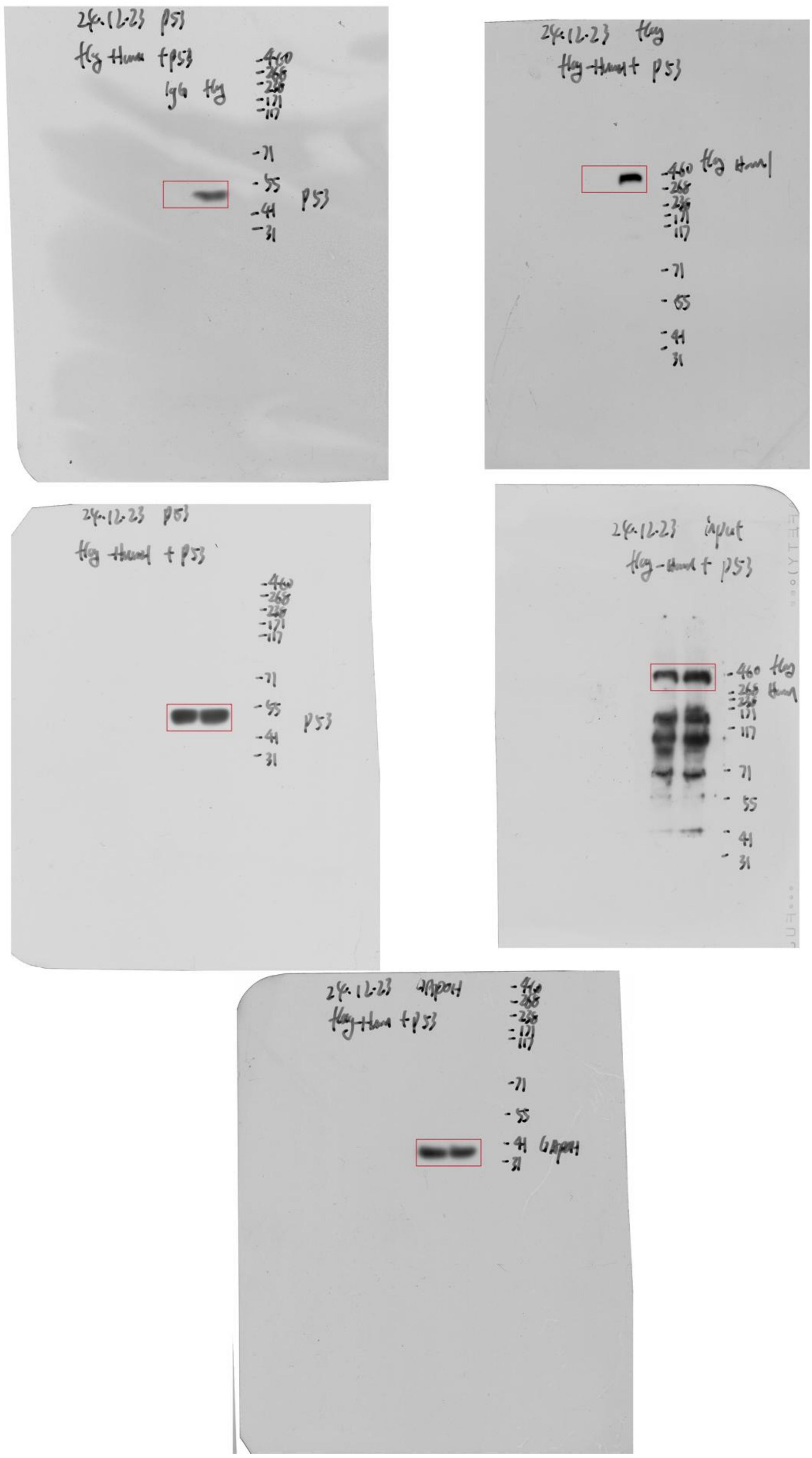

Fig. 4E

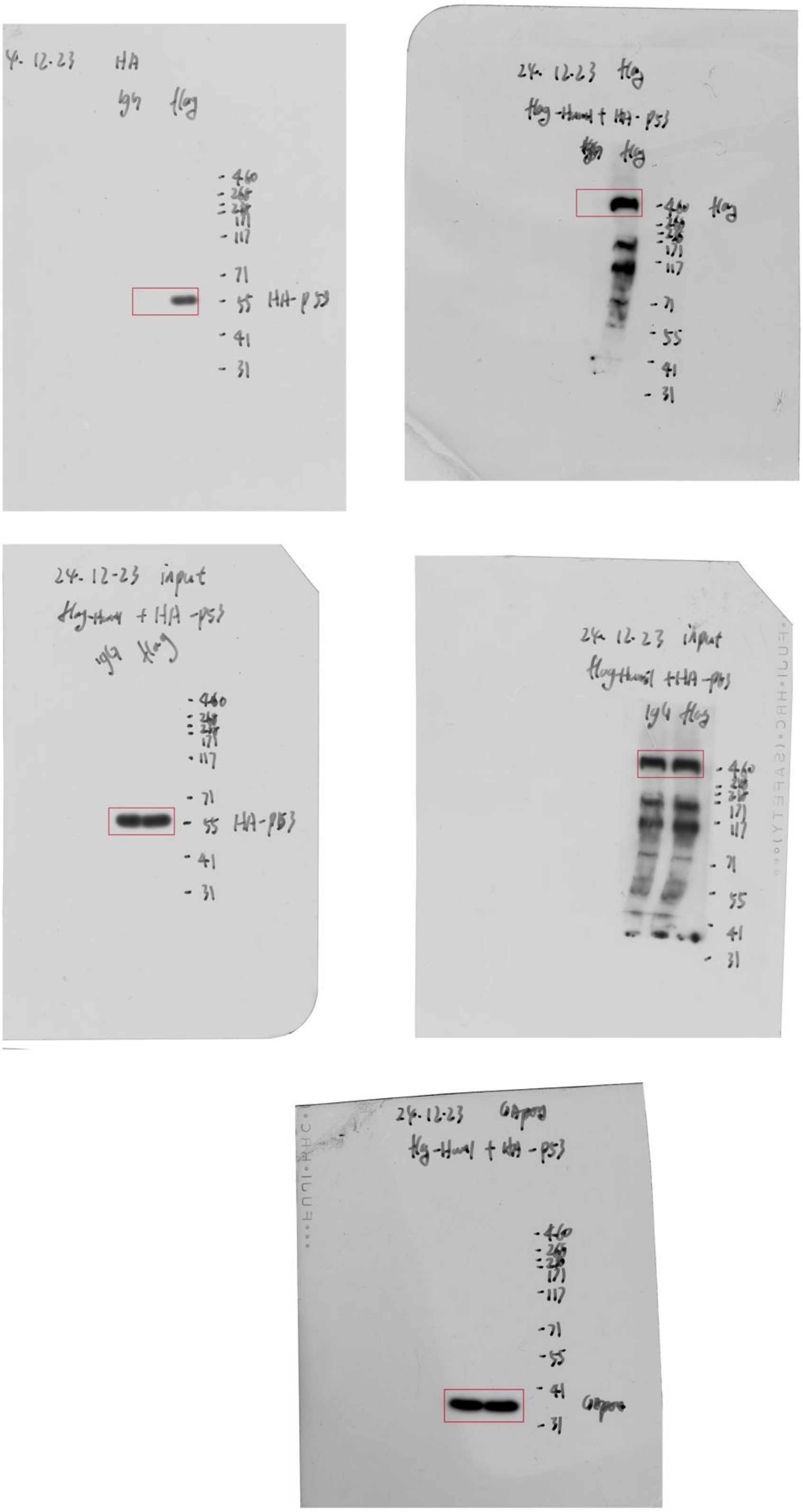

Fig. 4F

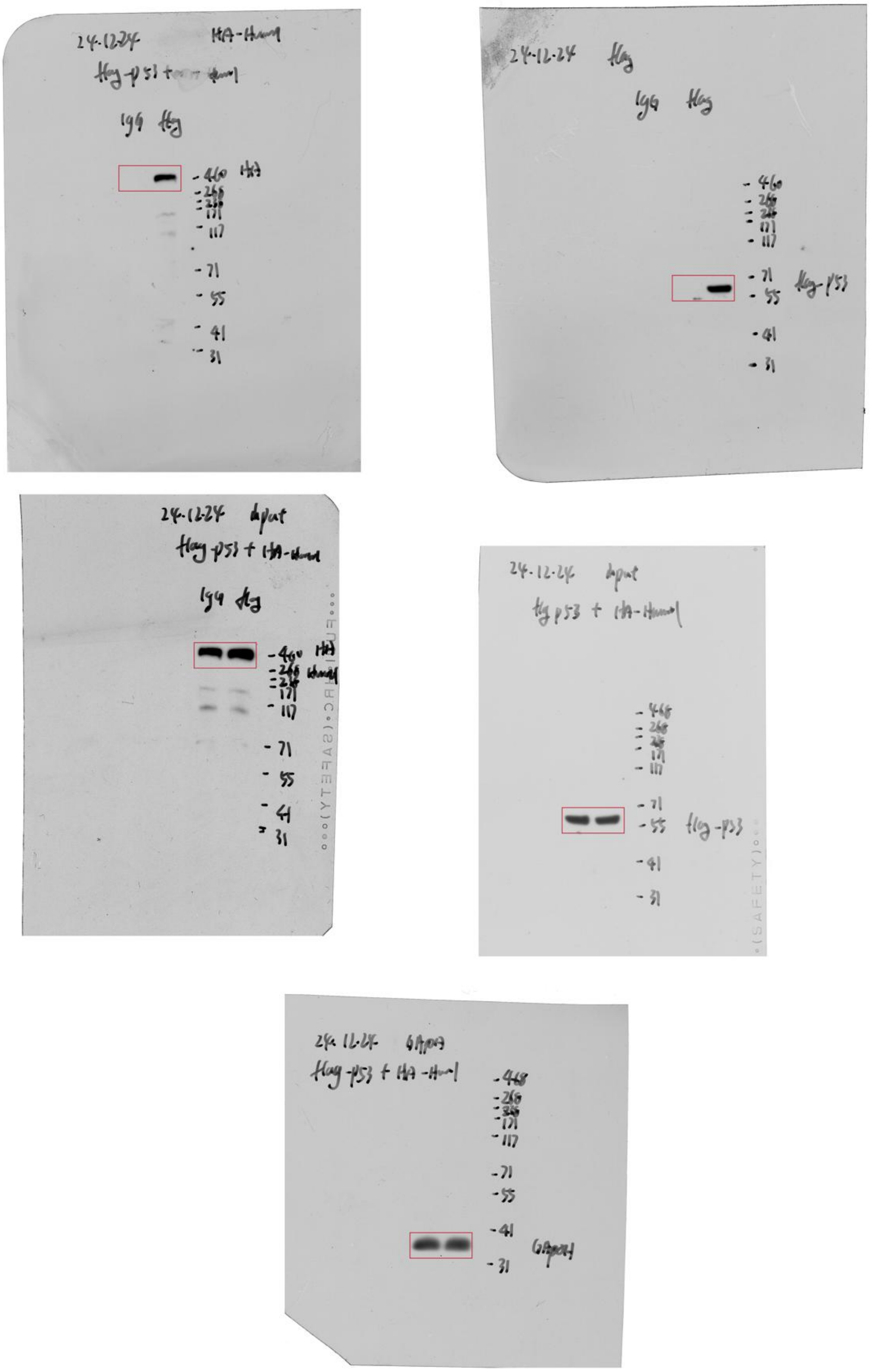

Fig. 4l

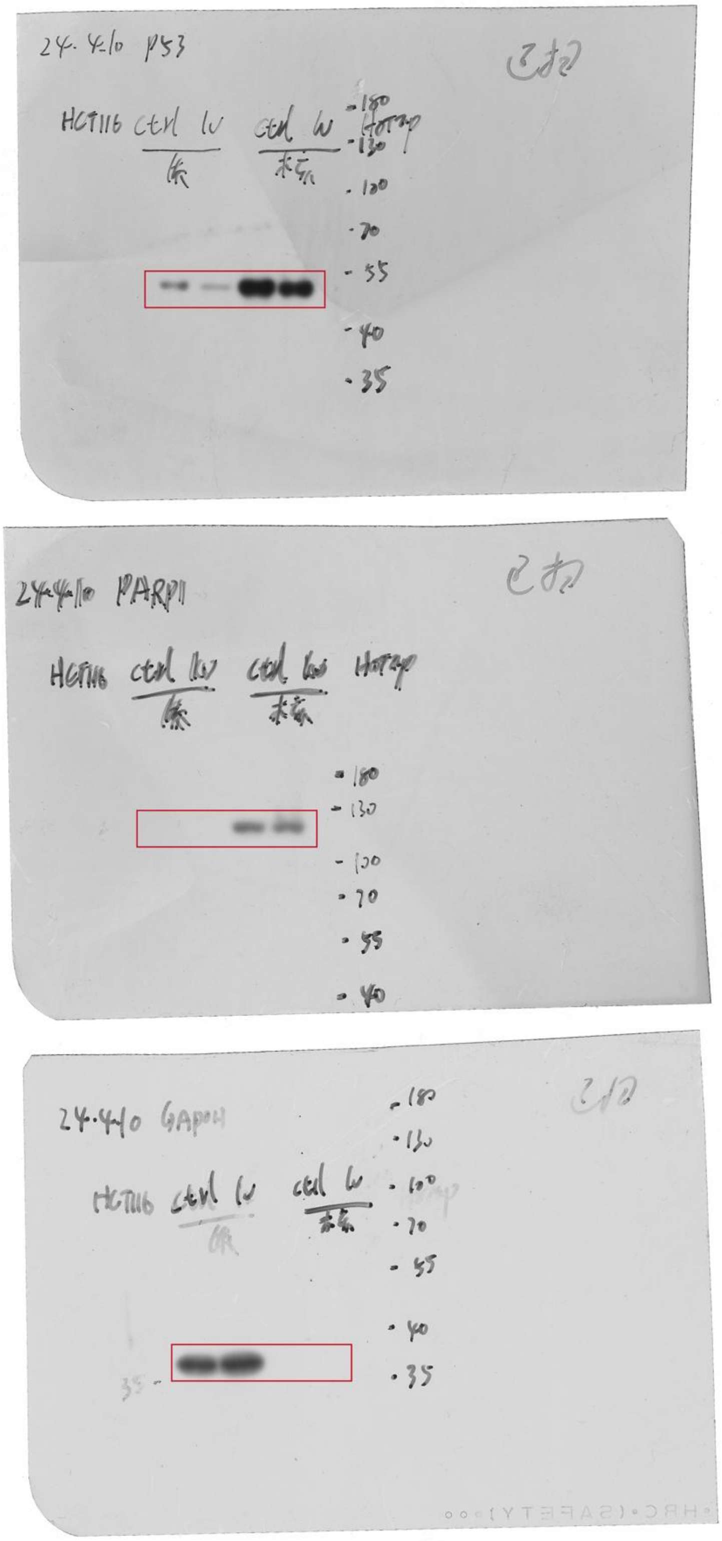
24.4.10 P53
HCT116
180
130
100
70
55
40
35
24.4.10 PARP1
HCT116
180
130
100
70
55
40
24.4.10 GAPDH
HCT116
180
130
100
70
55
40
35
HRC (SAFETY)

Fig. 4J

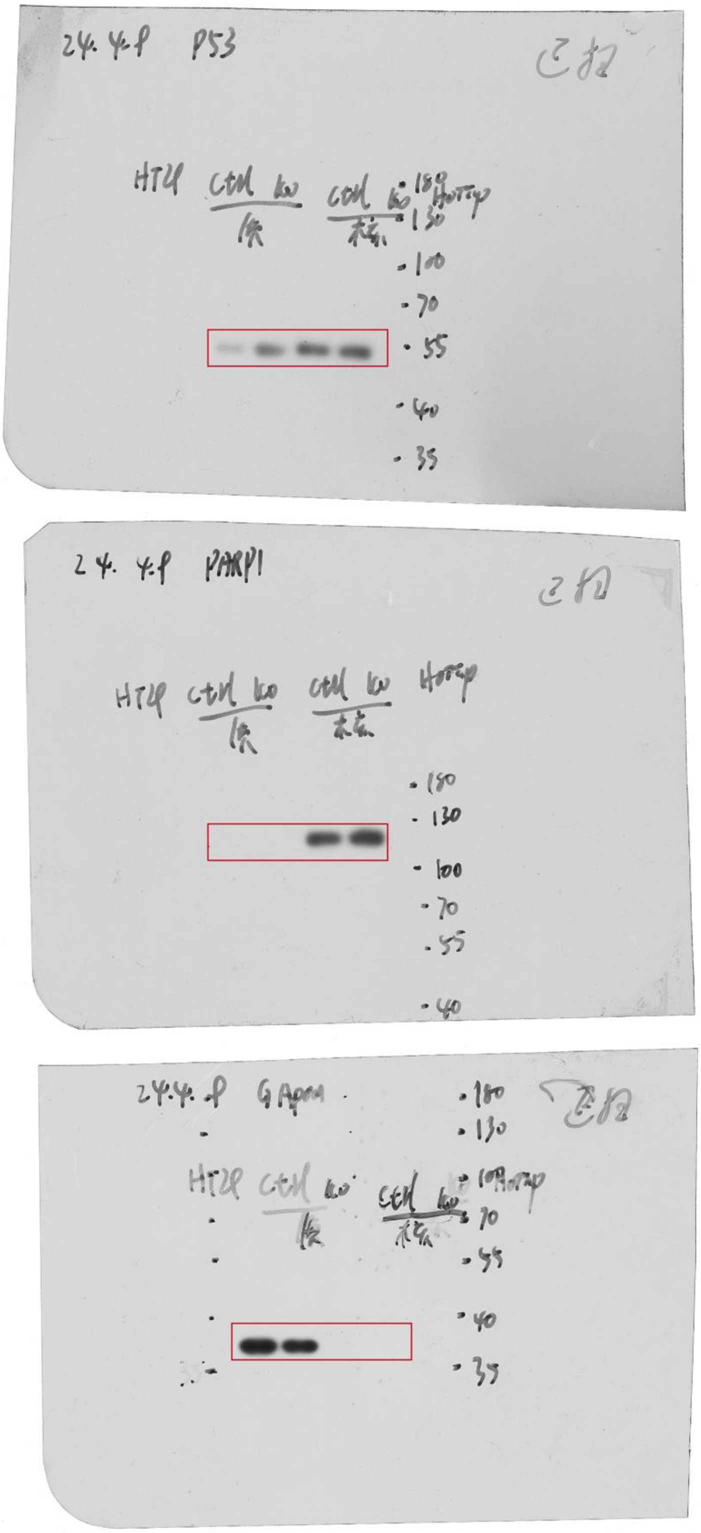
24.4.8 P53
HT29 ctrl ko ctrl ko Hotrp
180
130
100
70
55
40
35
24.4.8 PARP1
HT29 ctrl ko ctrl ko Hotrp
180
130
100
70
55
40
24.4.8 GAPDH
HT29 ctrl ko ctrl ko Hotrp
180
130
100
70
55
40
35

Fig. 4K

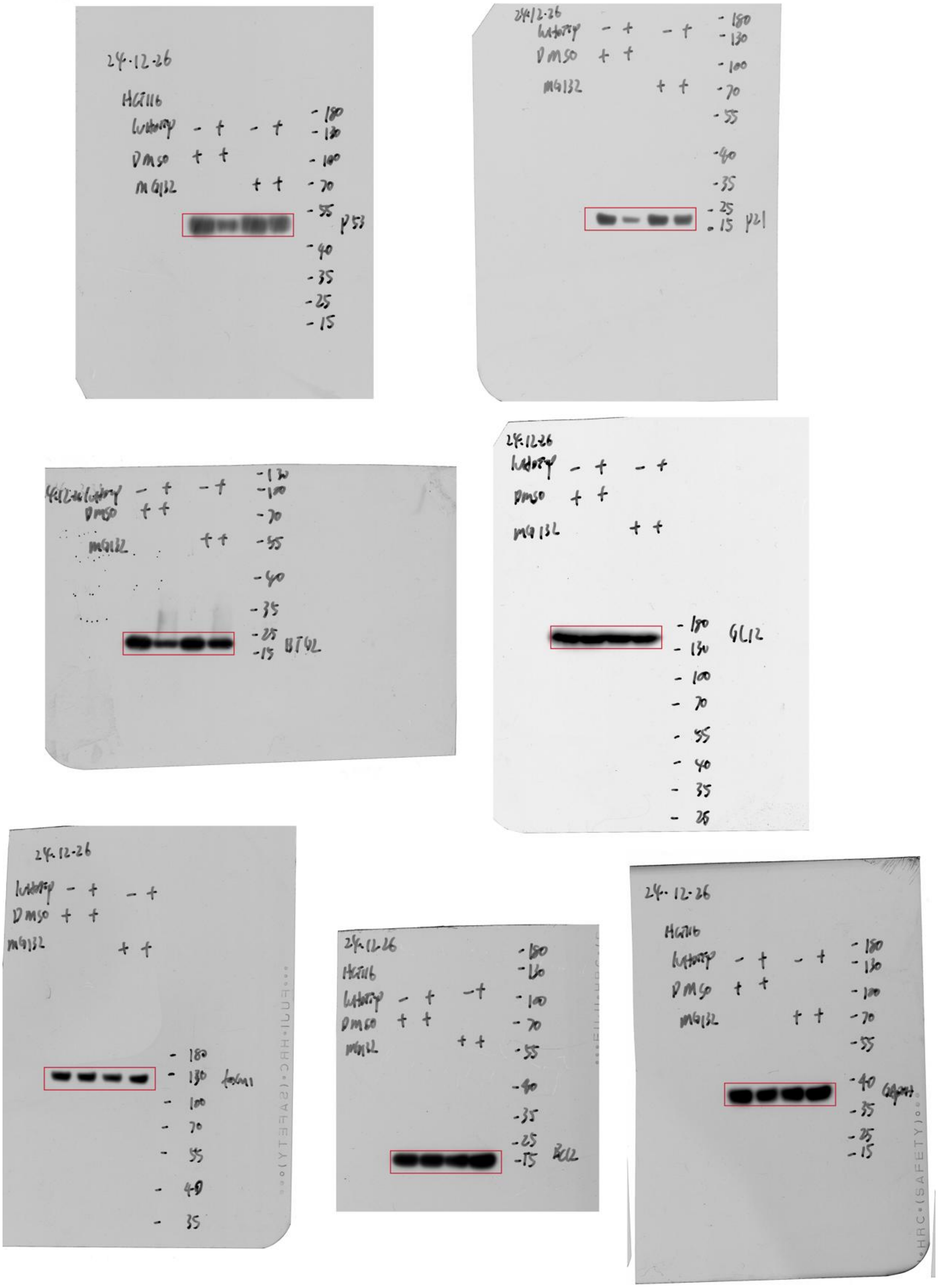

Fig. 4M

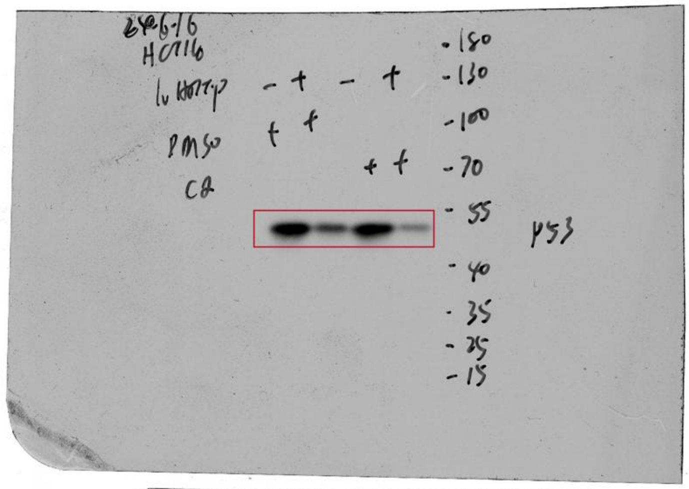
HCT116
DMSO
CQ
p53
180
130
100
70
55
40
35
25
15

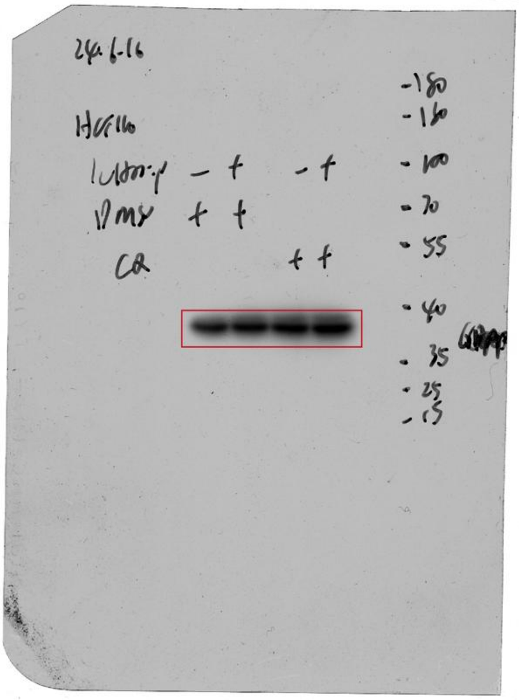
HCT116
CQ
180
130
100
70
55
40
35
25
15

Fig. 4N

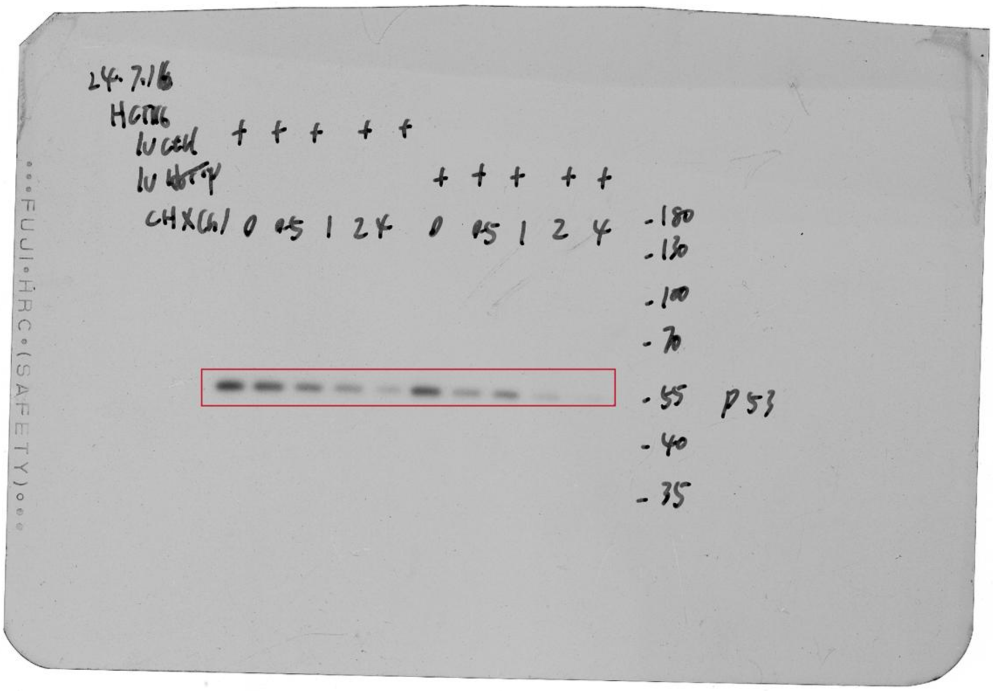

HCT116
CHX(h) 0 0.5 1 2 4 0 0.5 1 2 4
-180
-130
-100
-70
-55
-40
-35
p53
FUJI•HRC•(SAFETY)

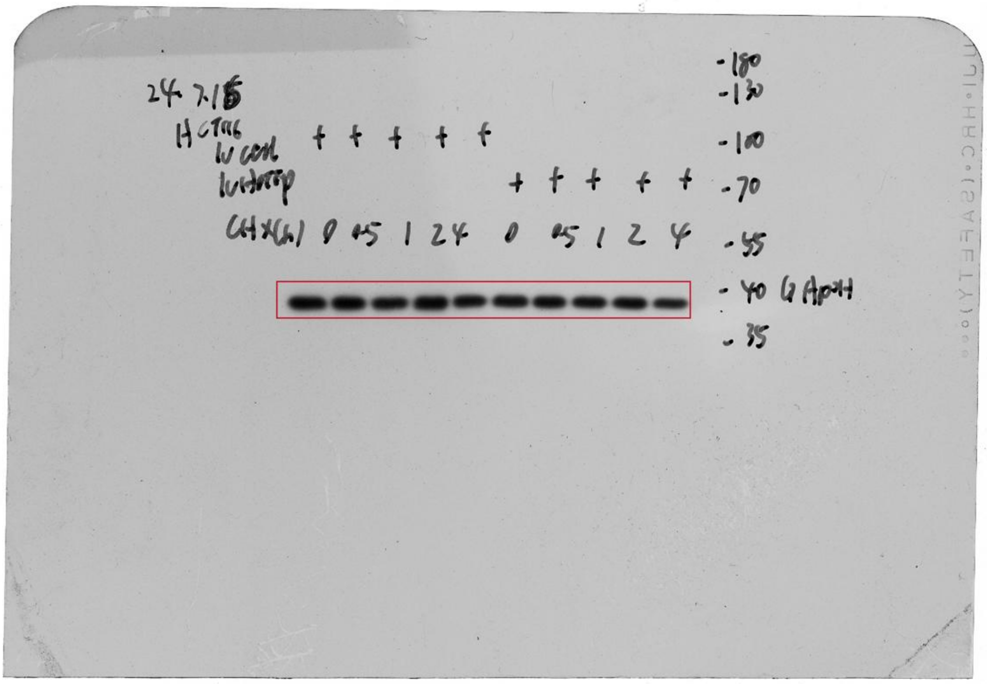

HCT116
CHX(h) 0 0.5 1 2 4 0 0.5 1 2 4
-180
-130
-100
-70
-55
-40
-35
GAPDH

Fig. 4P

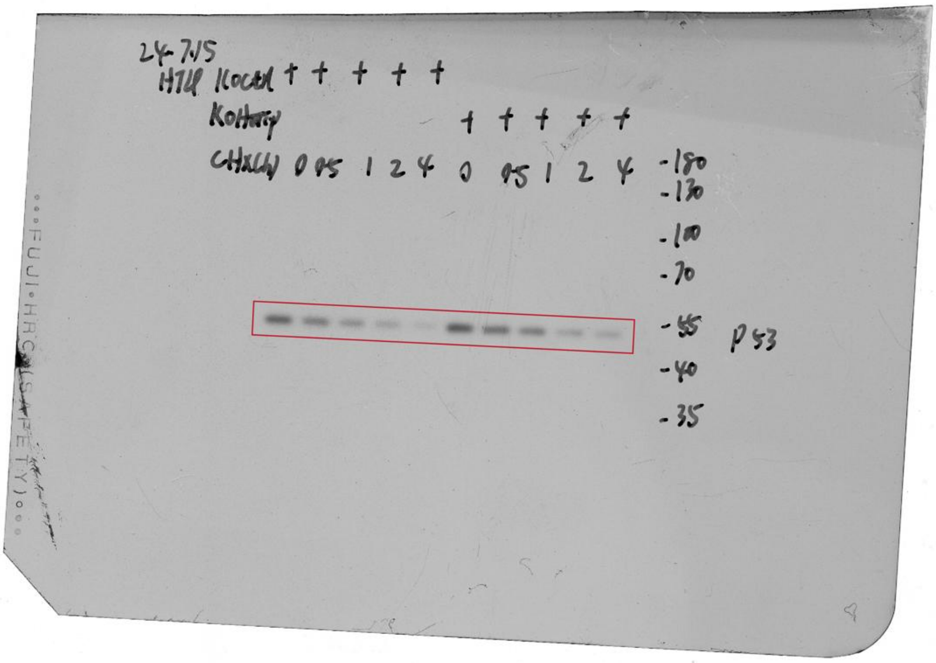

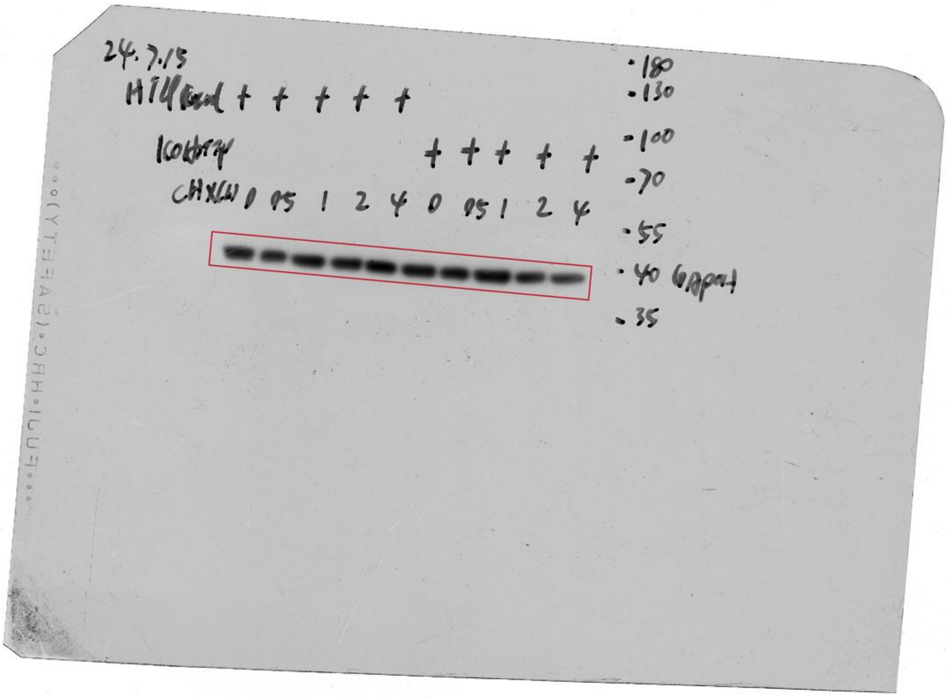

Fig. 4R

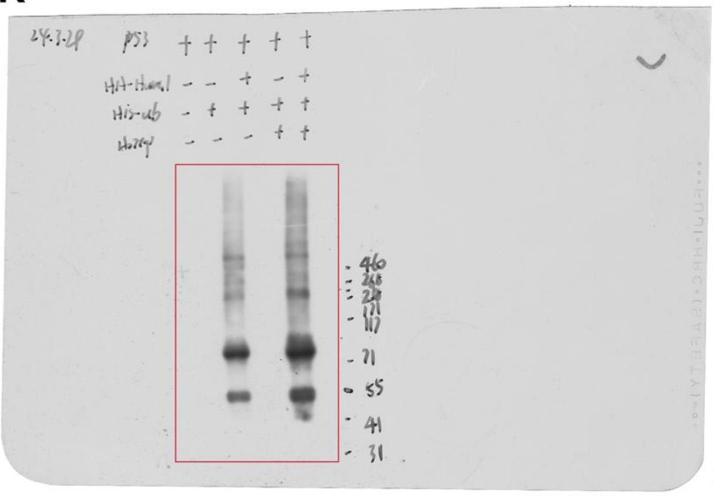

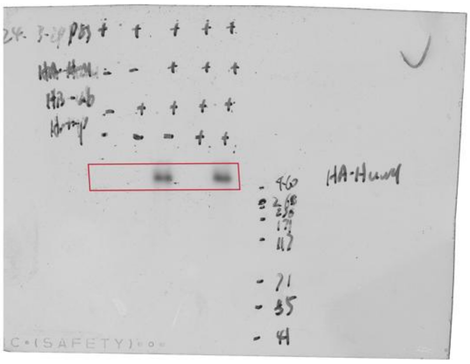

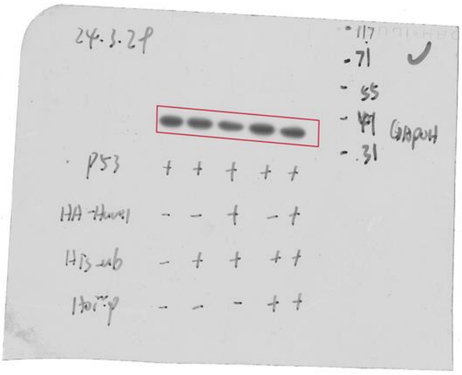

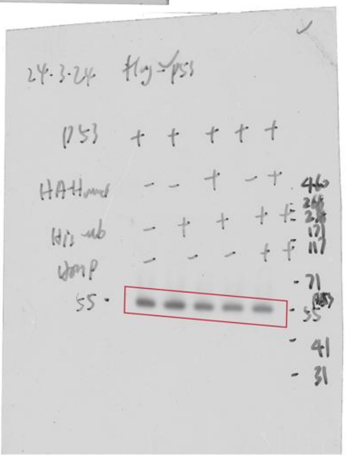

Fig. 5A

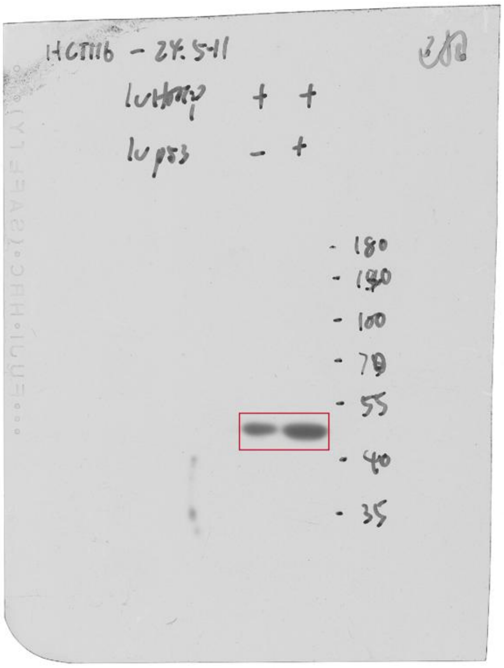

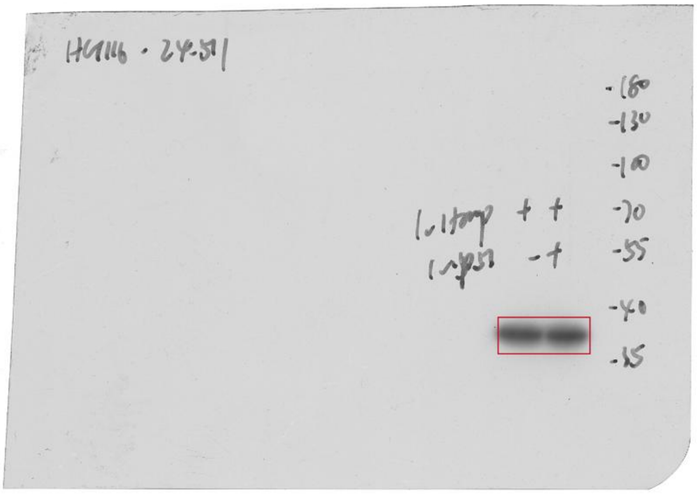

Fig. 5G

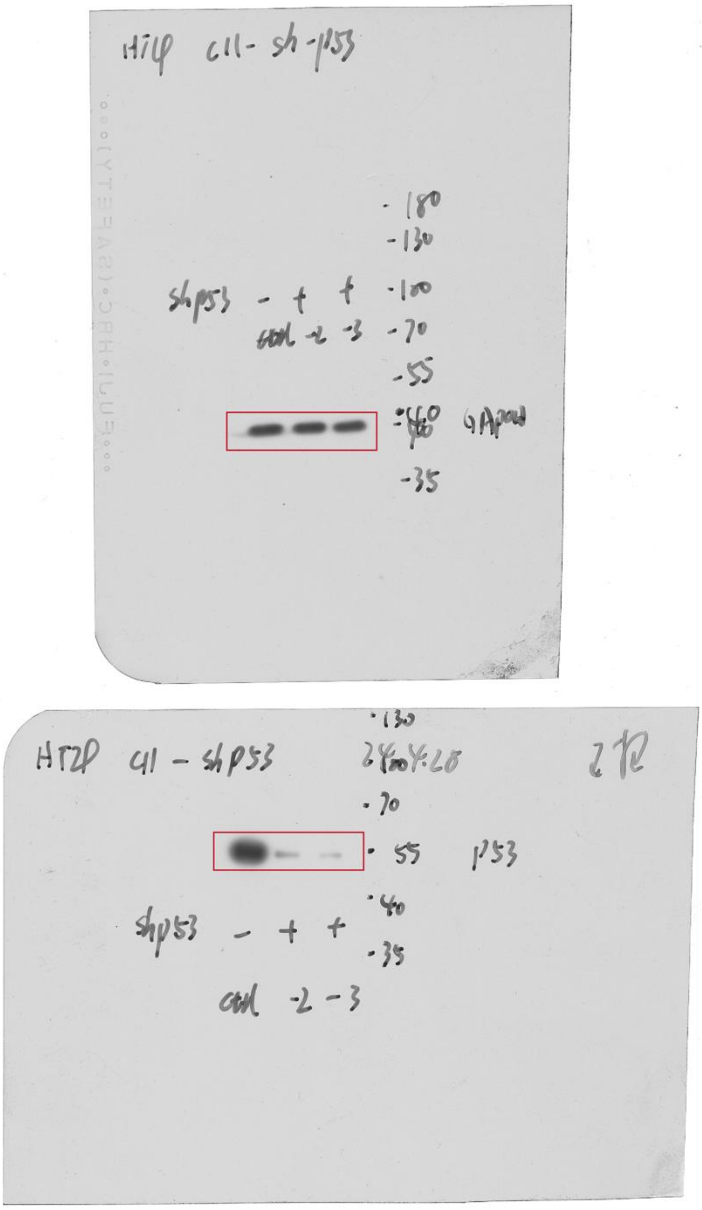
shp53 - + +
ctrl -2 -3
-180
-130
-100
-70
-55
GAPDH
-35
HT29 C11 - shp53
-70
55
p53
shp53 - + +
-35
ctrl -2 -3

Fig. 7D

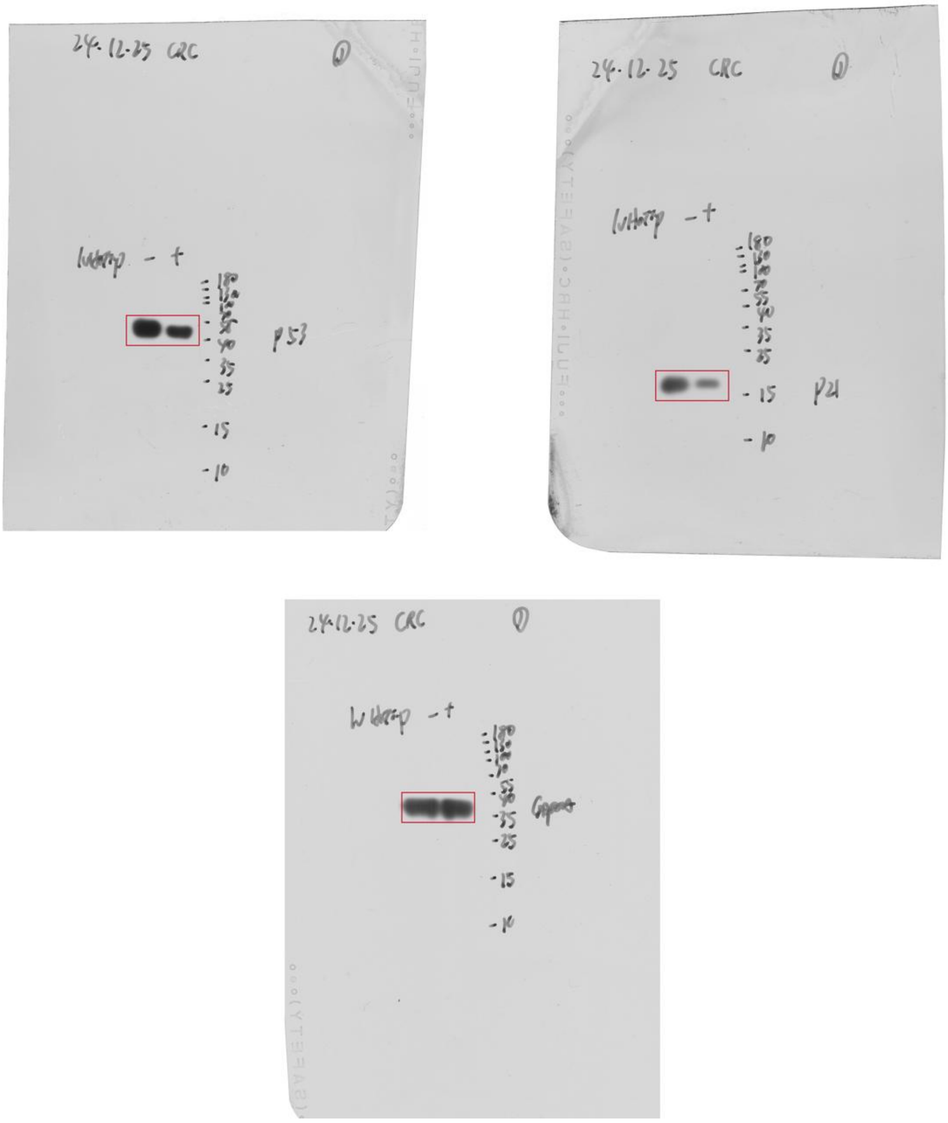

Fig. 7L

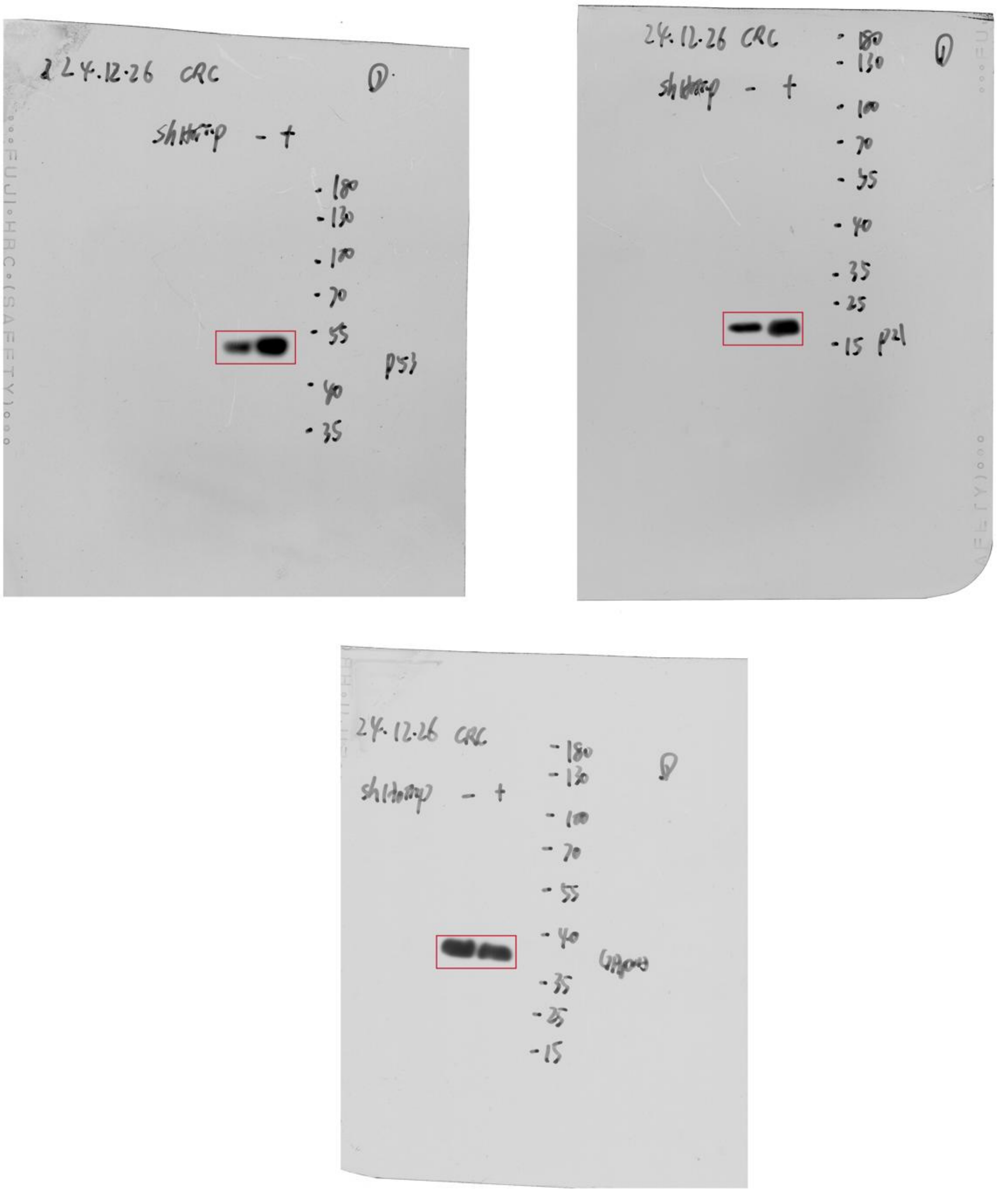

Fig. 8F

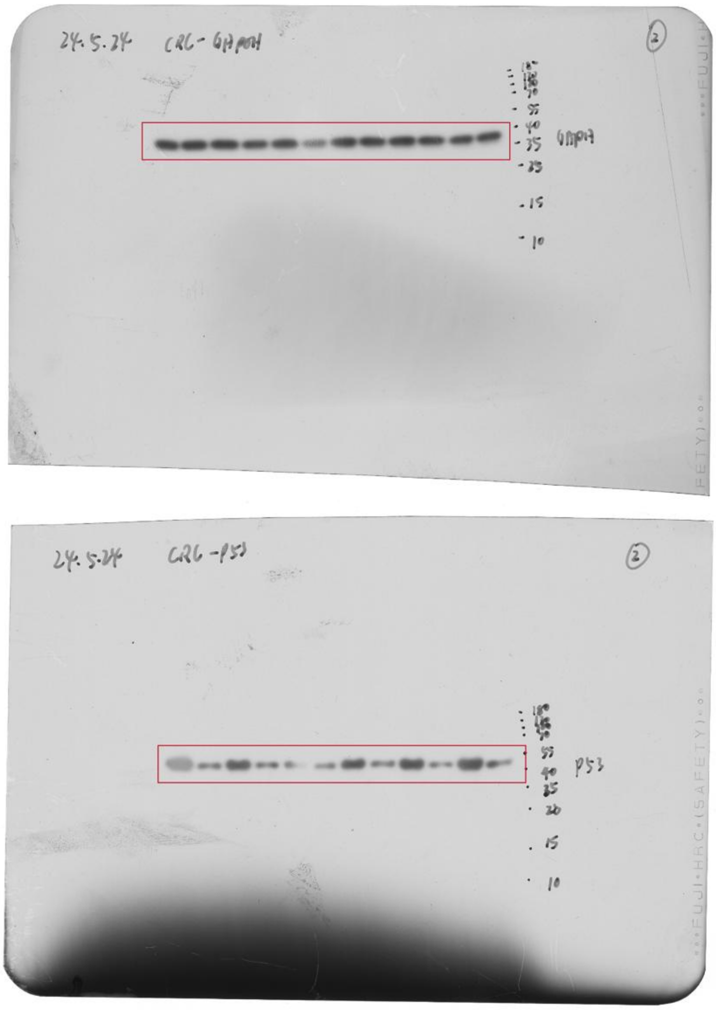

Fig. 8F

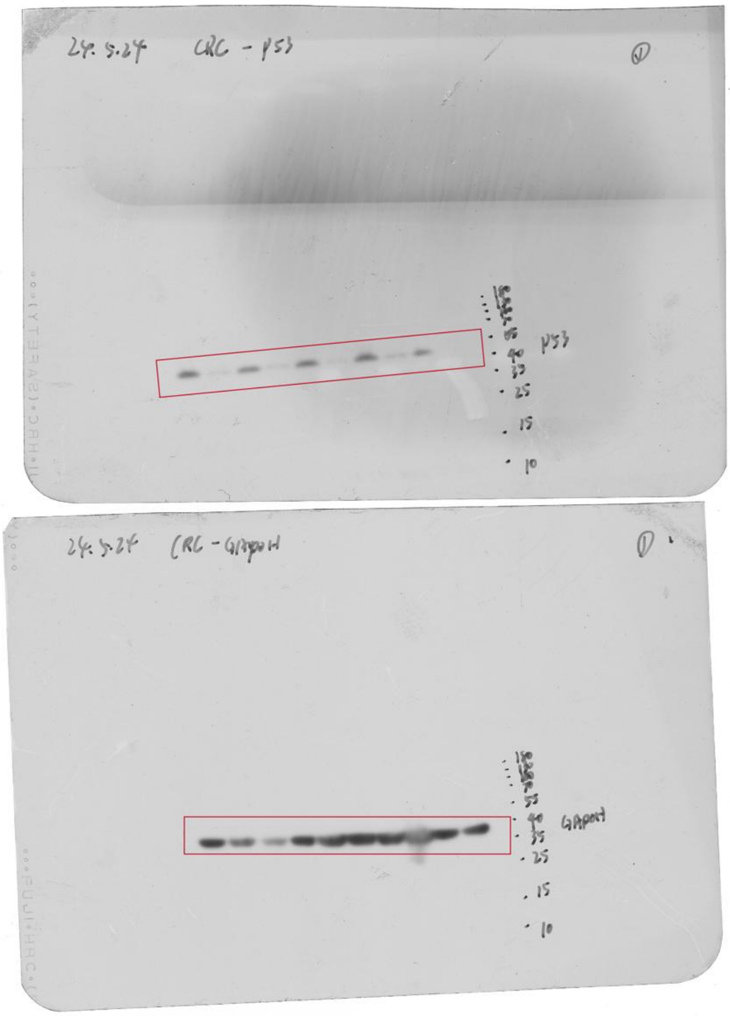

Supplementary Fig. 5F

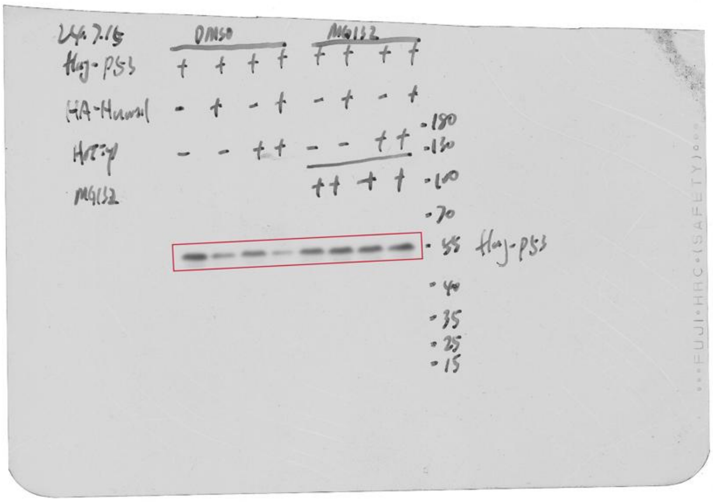

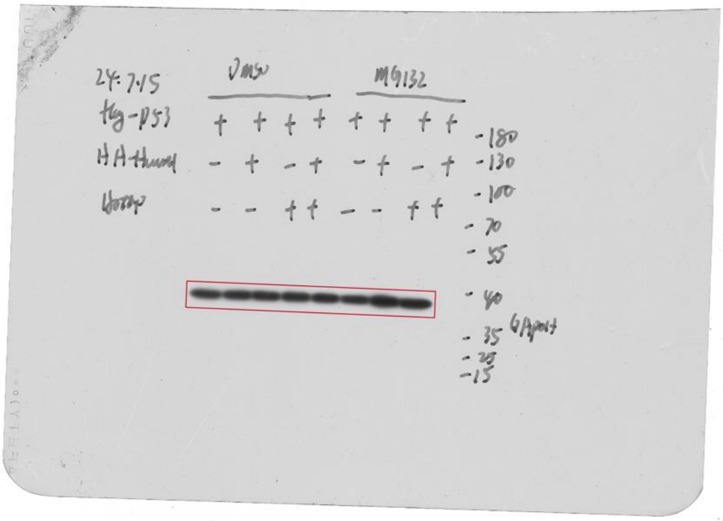

Supplementary Fig. 6E

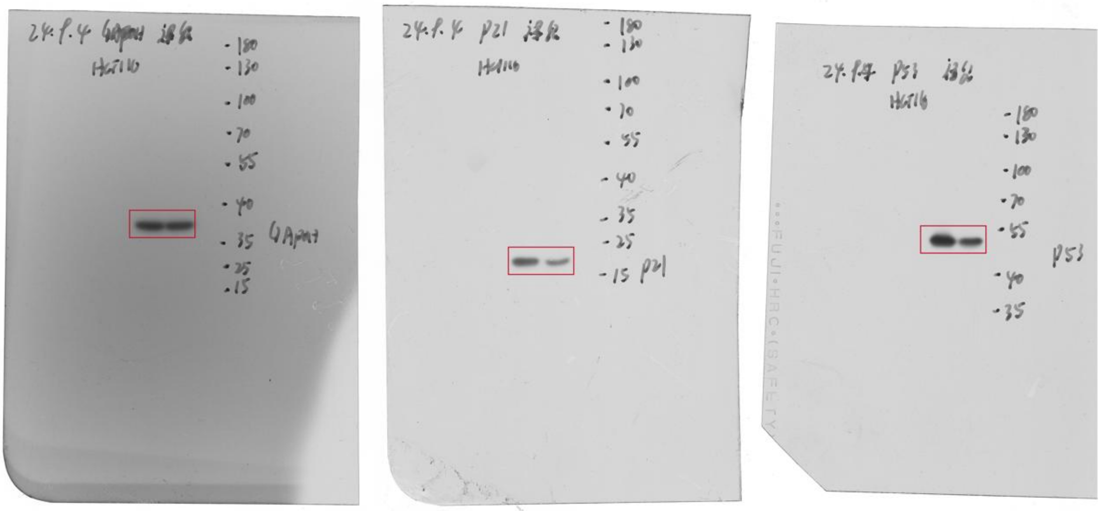

Supplementary Fig.6E

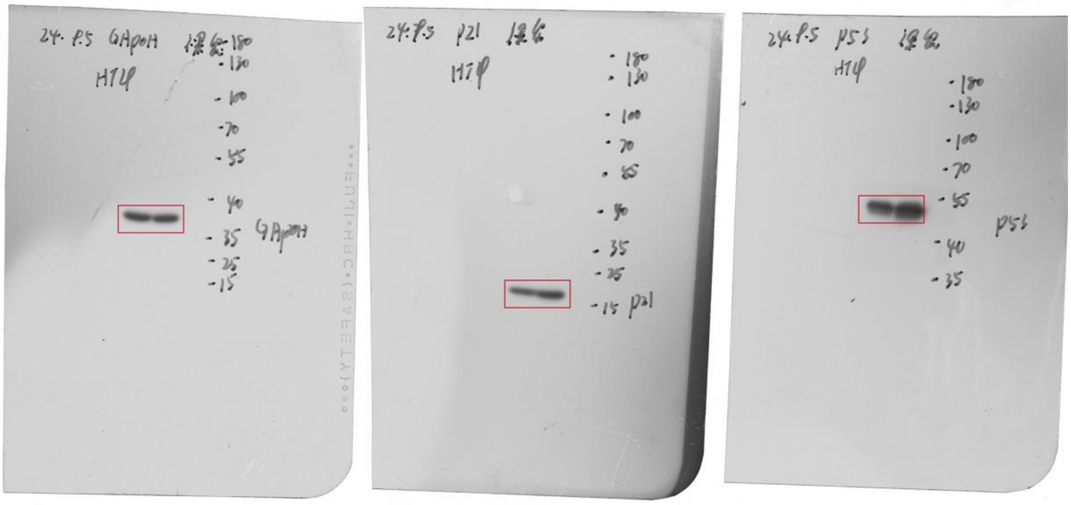

Supplement: Supplementary file 2 — Supplemental Material [file 41419_2025_7817_MOESM2_ESM.pdf]
